# Influence of lack of blinding on the estimation of medication-related harms: a retrospective cohort study of randomized controlled trials

**DOI:** 10.1186/s12916-024-03300-7

**Published:** 2024-03-07

**Authors:** Chang Xu, Fengying Zhang, Suhail A. R. Doi, Luis Furuya-Kanamori, Lifeng Lin, Haitao Chu, Xi Yang, Sheyu Li, Liliane Zorzela, Su Golder, Yoon Loke, Sunita Vohra

**Affiliations:** 1 Proof of Concept Center, Eastern Hepatobiliary Surgery Hospital, Third Affiliated Hospital, Second Military Medical University, Naval Medical University, Shanghai, China; 2 Chinese Evidence-Based Medicine Center, West China Hospital, Sichuan University, Chengdu, China; 3 Department of Population Medicine, College of Medicine, QU Health, Qatar University, Doha, Qatar; 4 UQ Center for Clinical Research, The University of Queensland, Herston, Australia; 5 Department of Epidemiology and Biostatistics, University of Arizona, Tucson, AZ USA; 6 Statistical Research and Innovation, Global Biometrics and Data Management, Pfizer Inc, New York, NY USA; 7 Division of Biostatistics and Health Data Science, University of Minnesota School of Public Health, Minneapolis, MN USA; 8 Department of Endocrinology and Metabolism, MAGIC China Centre, West China Hospital, Sichuan University, Chengdu, China; 9 Department of Pediatrics, Faculty of Medicine & Dentistry, University of Alberta, Edmonton, AB Canada; 10 Department of Health Sciences, University of York, York, UK; 11 Norwich Medical School, University of East Anglia, Norwich, UK; 12 Departments of Pediatrics & Psychiatry, Faculty of Medicine & Dentistry, University of Alberta, Edmonton, AB Canada

**Keywords:** Randomized controlled trials, Blinding, Harms, Adverse effects

## Abstract

**Background:**

Empirical evidence suggests that lack of blinding may be associated with biased estimates of treatment benefit in randomized controlled trials, but the influence on medication-related harms is not well-recognized. We aimed to investigate the association between blinding and clinical trial estimates of medication-related harms.

**Methods:**

We searched PubMed from January 1, 2015, till January 1, 2020, for systematic reviews with meta-analyses of medication-related harms. Eligible meta-analyses must have contained trials both with and without blinding. Potential covariates that may confound effect estimates were addressed by restricting trials within the comparison or by hierarchical analysis of harmonized groups of meta-analyses (therefore harmonizing drug type, control, dosage, and registration status) across eligible meta-analyses. The weighted hierarchical linear regression was then used to estimate the differences in harm estimates (odds ratio, OR) between trials that lacked blinding and those that were blinded. The results were reported as the ratio of OR (ROR) with its 95% confidence interval (CI).

**Results:**

We identified 629 meta-analyses of harms with 10,069 trials. We estimated a weighted average ROR of 0.68 (95% CI: 0.53 to 0.88, *P* < 0.01) among 82 trials in 20 meta-analyses where blinding of participants was lacking. With regard to lack of blinding of healthcare providers or outcomes assessors, the RORs were 0.68 (95% CI: 0.53 to 0.87, *P* < 0.01 from 81 trials in 22 meta-analyses) and 1.00 (95% CI: 0.94 to 1.07, *P* = 0.94 from 858 trials among 155 meta-analyses) respectively. Sensitivity analyses indicate that these findings are applicable to both objective and subjective outcomes.

**Conclusions:**

Lack of blinding of participants and health care providers in randomized controlled trials may underestimate medication-related harms. Adequate blinding in randomized trials, when feasible, may help safeguard against potential bias in estimating the effects of harms.

**Supplementary Information:**

The online version contains supplementary material available at 10.1186/s12916-024-03300-7.

## Background

The randomized controlled trial is the preferred and most rigorous study design in clinical research for assessment of medication efficacy [1]. In a randomized controlled trial, blinding is a vital procedure to mitigate bias. However, blinding may not always be achievable due to practical and/or ethical reasons. In many cases, blinding increases the difficulty of participant recruitment, complexity of implementation (e.g., preparing packaging of the interventions), and total costs of a trial [2]. In addition, blinding is difficult for non-pharmaceutical interventions. Lack of blinding results in knowledge of intervention assignment and may affect adherence and attrition or influence recording of outcomes, resulting in performance bias and measurement bias [3].

Empirical and/or meta-epidemiological studies are valuable sources of evidence that can help us examine the relationship between methodological weaknesses and their potential impact on research findings [4]. For example, empirical studies have demonstrated that a lack of blinding of participants, care providers, or outcome assessors may lead to exaggerated treatment effects [5–13]. However, existing empirical studies have focused mainly on efficacy or effectiveness, while few have addressed related questions on harms, including medication-related harms. This underemphasis on harms perpetuates the gap between evidence generation, evidence synthesis, and informed decision-making. As highlighted in the Cochrane Handbook, harms are considered just as important as effectiveness/efficacy in the evaluation of healthcare interventions [14].

Harm outcomes (especially those that are serious in nature) typically involve lower event rates than benefit outcomes, and the measurement of such harm outcomes can be substantially affected by random error [15, 16]. The occurrence of some obviously identifiable adverse reactions may overcome attempts to maintain blinding, thus increasing the possibility of participants, health care providers, and investigators being able to correctly discern the intervention [17–19]. Moreover, harm outcomes often involve the utilization of composite outcomes, which may result in selective reporting bias [20]. As a result, lack of blinding may have a differential impact on estimates of harm as compared to benefits. The potential impact of lack of blinding remains an important gap in research and clearly needs to be addressed, as it may have important implications for evidence-based practice, policy formulation, and informed decision-making.

In this large-scale meta-epidemiological study, we compared effect estimates of harm from blinded randomized trials as opposed to trials without blinding, which were otherwise comparable with regard to interventions, controls, and key methodological features.

## Methods

### Protocol and reporting

The present study is part of a large research program designed to investigate potential methodological factors that influence reporting of harms in randomized controlled trials. The protocol for this research program has been reported elsewhere [21]. We have formatted and reported our study in accordance with the Preferred Reporting Items for Overviews of Reviews (PRIOR) checklist where applicable, as this tool is the “up-to-date” version of all related guidelines [22].

### Data source

The study is based on our recently constructed large empirical dataset, known as SMART Safety [23, 24]. The foundations of this dataset stem from a PubMed literature search conducted on July 28, 2020, by an information specialist, with the aim of retrieving systematic reviews of medication harms that were published (including online first) between January 1, 2015, and January 1, 2020. [25]. The representativeness of the search has been verified earlier, with sensitivity ranging from 93.85 to 99.30% [21]. The full search strategy is reported in Additional file 1.

### Inclusion criteria

Systematic reviews of medication-related harms with harms as the exclusive outcome and with at least one meta-analysis were considered for eligibility. This means we did not consider systematic reviews that included efficacy/effectiveness outcomes, regardless of whether harms were treated as primary or secondary outcomes. For inclusion in the final analysis, the meta-analyses must have included at least five randomized controlled trials with two-by-two tabular data (comparison group and harm outcome) available for trials both with and without blinding. We defined a systematic review or meta-analysis on the basis of the article title as stipulated by the review authors. We defined harm outcomes as “*any untoward medical occurrence in a patient or subject in clinical practice*,” which include risk, complication, adverse effects, or adverse reaction, based on the PRISMA harms checklist [26].

We recognize that the restriction to a minimum of five studies may lead to a slight loss of the representativeness of the data in the current study. However, we also note that meta-analyses that contain only a few studies are less likely to be able to meet our eligibility requirement that both blinded and unblinded studies be available for harms outcome analysis [27].

Two authors (XQ, CX) independently screened the titles, abstracts (stage 1), and full-texts (stage 2) of the records using Rayyan (https://www.rayyan.ai/). Only those excluded by both authors were excluded during stage 1, and the remaining records were screened again in stage 2, with disagreements resolved through consensus.

### Data collection

Data collection was conducted using independent duplicate extraction (CX, TQ, FZ, XY, RZ, YT, XX, YZ, XZ, LFK, YY, HD); see details in Additional file 1 (Table S1 and Table S2). Three levels of data were collected: systematic review level, meta-analysis level, and trial level. For the systematic review level, the name of the review author, region of the review author, number of trials, and registration information were collected. At the meta-analysis level, we collected information on the outcome of interest. The following items were extracted at the study level: first author name, year of publication, journal, number of participants and number of events in each group (metadata), details of interventions and controls (e.g., type of intervention, dosage, duration), funding source (e.g., academic, industry), registration (Yes, No), average population age status (child, adult), trial centers and regions involved, and bias assessment information. All the study-level items, except for the metadata (i.e., 2 by 2 table data), were taken from the original trials. For the metadata (events, group size of each arm), we first extracted the information from the meta-analyses, either via forest plot or table. In order to avoid potential data extraction errors, we checked all data by referring to the original trials; any errors identified were further recorded and corrected [21].

We used an adaptation of the RoB 2 by selecting applicable components and domains for our assessment, without going through the entire algorithm and signaling process [28]. The parameters of specific interest were as follows: (1) random sequence generation; (2) allocation concealment; (3) blinding of participants; (4) blinding of healthcare providers; and (5) blinding of outcome assessors. To avoid potential confusion, we did not use the recommended “response options” of RoB 2; instead, we used the options of “Yes” or “Probably Yes” as studies that implemented blinding or probably implemented blinding and, similarly, “No” and “Probably No” for those that did not or probably did not implement blinding. The assessment of the risk of bias information was based on what was reported in the original trials and carried out independently in duplicate with any disagreements resolved by discussion (Additional file 1: Table S1 and S2).

We further categorized outcomes from each meta-analysis as objective or subjective. This was done independently by two senior methodologists (LFK, CX), and their decisions were compared by a third author (RZ) in a blinded manner. Further online discussion was employed for disagreements until consensus was achieved. The criteria for the judgment of the type of outcomes were based on the explanatory file of RoB 2 [28].

All data collected were double-checked to minimize errors in data extraction. The details of the contributors to data extraction are recorded in Additional file 1 (Tables S1 and S2).

### Outcomes

We pre-defined the primary outcome in this investigation as the ratio of the harm estimates in trials with and without blinding (participants, healthcare providers, and trial outcome assessors). Based on the RoB assessment, we dichotomized the blinding status of trials as follows: those clearly claiming implementation of blinding (judged as “Yes,” see above) or probably implemented blinding (judged as “Probably Yes”), while the rest were considered to be without blinding (judged as “No,” “Probably No,” and “No information”). No secondary outcomes were defined.

### Control of confounding

We recognize that trials with blinding may not share exactly the same characteristics as trials without blinding. As such, “third factors” or covariates that may have a confounding impact on our comparative evaluation of effect estimates from trials with and without blinding were identified and accounted for. From our review of the relevant literature [9, 29], we identified the following potential covariates that may influence estimates of harms: (1) specific features of the interventions; (2) nature of the controls; (3) variation in dosage of the intervention (mean dose per week); (4) treatment duration; (5) average age of the trial population; (6) source of funding (e.g., academic, industry, not reported); (7) role of funder; (8) number of centers; (9) trial registration; and (10) analytic protocol (e.g., intention-to-treat, per-protocol). We further conducted a causal path analysis via directed acyclic graphs (http://dagitty.net/) to identify which of these covariates may confound the association between blinding status and effect estimates for harm in randomized trials [30].

In order to reduce confounding and additionally assess the direct effect of the absence of blinding, we implemented restriction and stratification of selected important covariates to harmonize the sets of trials being compared. For example, with regard to intervention dose, only trials with the same dose (e.g., 50 mg/daily) could be grouped together in meta-analyses where trials with and without blinding were being compared. Through restriction and stratification of trials on reported values of these important covariates, we were able to conduct an analysis harmonized across groups of trials that shared similar attributes. We believe that this analytic approach (based on comparisons of blinded and unblinded trials within each harmonized group) leads to less confounded estimates of the relative differences between trials. See Additional file 1: Fig. S1 for more details.

Potential confounders were addressed through the covariate-harmonization process between trials in the comparisons of blinding status. Restriction was used to limit trials such that those that were included had similar pharmaceutical formulation, daily dose, and type of control within each meta-analysis. Stratification was also used across trials to create a covariate for harmonized groups by age category (child or adult participants), analytic protocol (e.g., intention-to-treat, ITT), trial registration, and allocation concealment. See Additional file 1: Figs. S2 and S3.

### Statistical analysis

Baseline characteristics were summarized as proportions or median and interquartile ranges (IQR). We first calculated the log odds ratio (OR) of each eligible trial for harm estimates of the intervention compared to control. A weighted hierarchical linear regression was then employed to estimate the ratio of OR (ROR) of trials with and without blinding by treating the trial as level one and the variable for covariate-harmonized groups as level two, with cluster robust standard errors to account for potential within-topic correlation of the groups [31]. When zero events occurred, we applied a continuity correction by adding 0.5 to each cell to estimate the OR within a trial [17].

We conducted sensitivity analysis according to the aforementioned pre-defined categorization, i.e., objective and subjective outcomes. The rationale for this approach was that previous studies have shown that objective outcomes are less susceptible to methodological issues involving blinding [32]. Post hoc sensitivity analysis was conducted by excluding studies with zero events [33]. Since we observed some imbalance of four trial characteristics for blinded versus unblinded trials, additional post hoc sensitivity analyses were employed.

Missing data occurred in 19 variables in the SMART Safety dataset, which ranged from 3.08 to 27.54%, mainly due to insufficient reporting, with a small minority missing due to inability to access full-text versions of trial reports (Additional file 1: Table S3). For the 15 variables we used in this study, the missing proportion ranged from 3.08 to 14.26%, and only two exceeded 10% (treatment duration in intervention and control group). We judged that the proportion of missing data in the remaining trials following the covariate-harmonization process would be small, and we therefore removed trials with missing data with the expectation that there would be little impact on our results [34]. All data analyses were run via Stata/SE 16.0 (Stata Corp LCC, College Station, TX), with two-sided alpha of 0.05 as the significance level. The code for the analysis is presented in Additional file 1.

## Results

**Fig. 1:**
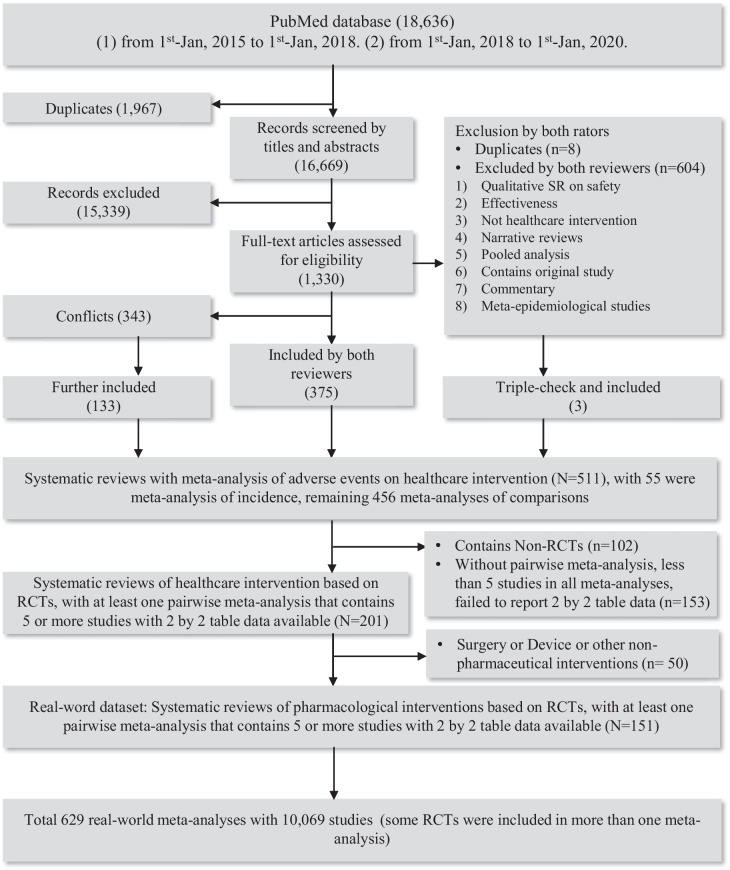
Flow diagram of literature screening

The search identified 18,636 records. After removing 1967 duplicates (searched separately before and after 1 January 2018) and 15,339 obviously out of scope based on titles and abstracts, 1330 records remained to be assessed for eligibility via full-texts. Among these, 151 systematic reviews with 629 meta-analyses involving 10,069 studies were identified as eligible (Fig. 1). The list of included and excluded systematic reviews (with reasons) can be accessed in Additional file 1 (Table S4). Table 1 presents baseline characteristics of our dataset, and Additional file 1: Fig. S4 presents word clouds of the related harm outcomes.

**Table 1:** Basic characteristics of eligible systematic reviews and trials

Basic characteristics	Summary
**Region of corresponding author** (review level)	*N* = 151
Africa	9 (5.96%)
Americas (North and South)	32 (21.19%)
Asia	68 (45.03%)
Europe	40 (26.49%)
Oceania	2 (1.32%)
**Number of trials included** (review level)	16 (IQR: 10 to 26)
1 to 9 (minimum was 5)	36 (23.84%)
10 to 29	83 (54.30%)
30 or more (maximum was 195)	33 (21.85%)
**Effect estimates** (review level)	
Odds ratio (including Peto odds ratio)	47 (31.13%)
Risk ratio	96 (63.58%)
Risk difference	3 (1.99%)
Others (e.g., HR, IRR, or above combinations)	5 (3.31%)
**Protocol** (review level)	
Yes	43 (28.48%)
No	108 (71.52%)
**Registration** (study level)	*N* = 10,069
Yes	7483 (74.32%)
No	2408 (23.91%)
Missing	178 (1.77%)
**Center** (study level)	
Multiple centers	7891 (78.37%)
Single center	350 (3.48%)
Missing	1828 (18.15%)
**Funding** (study level)	
Industry	8440 (83.82%)
Industry and academic	108 (1.07%)
Academic	737 (7.32%)
No funding	3 (0.03%)
Missing	781 (7.76)
**Publication type of study** (study level)	
Article	9848 (97.81%)
Abstract	18 (0.18%)
Registration only (unpublished)	198 (1.97%)
Non-RCT (further removed)	5 (0.05%)
**Accessible of full-text** (study level)	*N* = 9,848
Yes	9598 (97.46%)
No	250 (2.54%)

*HR* hazard ratio, *IRR* incidence risk ratio, *RCT* randomized controlled trial

After removing trials with missing data, 7693 (76.40%) studies from 607 meta-analyses remained for analysis. From the latter, we carried out restriction on trials to harmonize covariates, resulting in 82 trials within 25 covariate-harmonized groups (in 20 meta-analyses) being eligible for analysis of lack of blinding of participants on harm estimates, 81 trials within 26 covariate-harmonized groups (in 22 meta-analyses) being eligible for analysis of lack of blinding of care providers on harm estimates, and 858 trials within 268 covariate-harmonized groups (in 155 meta-analyses) being eligible for analysis of lack of blinding of outcome assessors on harm estimates. Characteristics of included trials within these covariate-harmonized groups are presented in Table 2.

**Table 2:** Trial characteristics of the comparisons

Trial characteristics	Trials blinded for ~	Trials unblinded for ~
**Participants**		
**Funding sources**		
Industry	41 (73.2%)	22 (84.6%)
Academic	12 (21.4%)	3 (11.5%)
Others	3 (5.4%)	1 (3.9%)
**Year of publication**		
~ 2000	0	0
2001 ~ 2010	24 (42.9%)	19 (73.1%)
2011 ~ 2020	32 (57.1%)	7 (26.9%)
**Region**		
Europe and North America	10 (19.6%)	6 (25.0%)
Multiple countries	27 (52.9%)	16 (66.7%)
Others	14 (27.5%)	2 (8.3%)
**Sample size**		
Less than 500	37 (66.1%)	10 (38.5%)
500 and more	19 (33.9%)	16 (61.54%)
**Health care providers**		
**Funding sources**		
Industry	42 (77.8%)	24 (88.9%)
Academic	8 (14.8%)	2 (7.4%)
Others	4 (7.4%)	1 (3.7%)
**Year of publication**		
~ 2000	0	0
2001 ~ 2010	24 (44.4%)	20 (74.1%)
2011 ~ 2020	30 (55.6%)	7 (25.9%)
**Region**		
Europe and North America	5 (20.8%)	6 (12.5%)
Multiple countries	17 (70.8%)	28 (58.3%)
Others	2 (8.3%)	14 (29.2%)
**Sample size**		
Less than 500	11 (40.7%)	34 (63.0%)
500 and more	16 (59.3%)	20 (37.0%)
**Outcome assessors**		
**Funding sources**		
Industry	498 (93.4%)	305 (93.3%)
Academic	28 (5.3%)	6 (1.8%)
Others	7 (1.3%)	16 (4.9%)
**Year of publication**		
~ 2000	2 (0.4%)	1 (0.3%)
2001 ~ 2010	125 (23.5%)	69 (21.1%)
2011 ~ 2020	406 (76.2%)	257 (78.6%)
**Region**		
Europe and North America	114 (21.6%)	47 (15.1%)
Multiple countries	335 (63.6%)	248 (79.5%)
Others	78 (14.8%)	39 (12.5%)
**Sample size**		
Less than 500	411 (77.1%)	214 (65.4%)
500 and more	122 (22.9%)	113 (34.6%)

### Lack of blinding of participants on harm effects

Based on 82 trials within 25 covariate-harmonized groups, our regression analysis showed that for overall harms, the ROR for trials lacking blinding was 0.68 (95% CI: 0.53 to 0.88, *P* < 0.01) compared to trials blinded for participants.

**Fig. 2:**
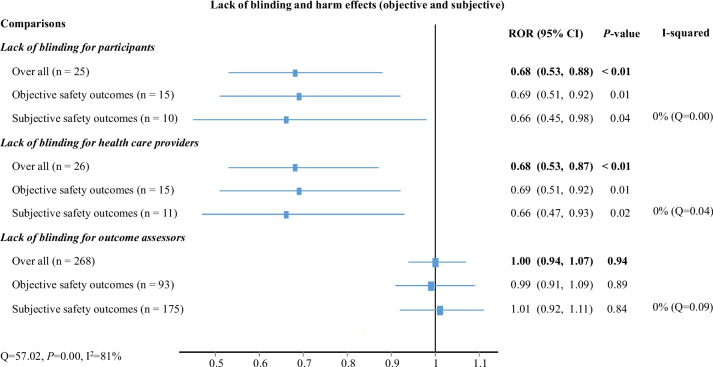
Influence of lack of blinding on harm effects

When stratified by type of outcome, the ROR for trials lacking blinding was 0.69 (95% CI: 0.51 to 0.92, *P* = 0.01, *n* = 51) for objective outcomes and 0.66 (95% CI: 0.45 to 0.98, *P* = 0.04, *n* = 31) for subjective outcomes when compared to trials blinded for participants (Fig. 2).

### Lack of blinding of health care providers on harm effects

Based on 81 trials within the 26 covariate-harmonized groups, our regression analysis showed that, for overall harm, the ROR for trials lacking blinding was 0.68 (95% CI: 0.53 to 0.87, *P* < 0.01) compared to trials blinded for health care providers.

When stratified by type of outcome, the ROR for trials lacking blinding was 0.69 (95% CI: 0.51 to 0.92, *P* = 0.01, *n* = 51) for objective outcomes and 0.66 (95% CI: 0.47 to 0.93, *P* = 0.02, *n* = 30) for subjective outcomes compared to trials blinded for health care providers; see Fig. 2.

### Lack of blinding of trial outcome assessors on harm effects

Based on 858 trials within the 268 covariate-harmonized groups, our regression analysis showed that for overall harm, the ROR for trials lacking blinding was 1.00 (95% CI: 0.94 to 1.07, *P* = 0.94) compared to trials blinded for outcomes assessors.

When stratified by type of outcome, the ROR for trials lacking blinding was 0.99 (95% CI: 0.91 to 1.09, *P* = 0.89, *n* = 340) for objective outcomes and 1.01 (95% CI: 0.92 to 1.11, *P* = 0.84, *n* = 508) for subjective outcomes compared to trials with blinded outcomes assessors; see Fig. 2.

### Sensitivity analyses

Sensitivity analysis by removing studies with zero events showed no substantial changes, with a ROR for lack of participant blinding of 0.64 (95% CI: 0.42, 0.97, *P* = 0.04), ROR for lack of health care provider blinding of 0.68 (95% CI: 0.53 to 0.87, *P* < 0.01), and ROR for lack of outcome assessor blinding of 1.01 (95% CI: 0.94 to 1.08, *P* = 0.84). Additional post hoc sensitivity analyses found the impact of blinding to be consistent under different sub-settings (Table 3).

**Table 3:** Post hoc sensitivity analyses

Sensitivity analyses	Lack of blinding on participants	Lack of blinding on health care providers	Lack of blinding on outcome assessors
**Funding sources**			
Industry funded	0.65 (95% CI: 0.48 to 0.88)	0.65 (95% CI: 0.49 to 0.88)	1.01 (95% CI: 0.94 to 1.08)
**Year of publication**			
2000 ~ 2010	0.73 (95% CI: 0.57 to 0.93)	0.73 (95% CI: 0.57 to 0.93)	0.90 (95% CI: 0.68 to 1.18)
2011 ~ 2020	0.38 (95% CI: 0.22 to 0.67)	0.33 (95% CI: 0.23 to 0.48)	1.03 (95% CI: 0.96 to 1.10)
**Region**			
Europe and North America	0.95 (95% CI: 0.20 to 4.53)	0.75 (95% CI: 0.19 to 2.90)	0.90 (95% CI: 0.72 to 1.13)
Multiple countries	0.65 (95% CI: 0.47 to 0.90)	0.66 (95% CI: 0.48 to 0.90)	0.95 (95% CI: 0.88 to 1.02)
Others	0.22 (95% CI: 0.15 to 0.31)	0.22 (95% CI: 0.15 to 0.30)	0.84 (95% CI: 0.49 to 1.44)
**Sample size**			
Less than 500	0.56 (95% CI: 0.36 to 0.86)	0.50 (95% CI: 0.34 to 0.73)	0.91 (95% CI: 0.80 to 1.03)
500 or more	0.68 (95% CI: 0.51 to 0.91)	0.68 (95% CI: 0.51 to 0.91)	1.05 (95% CI: 0.95 to 1.16)

## Discussion

In this study, we used a large empirical dataset to investigate the influence of blinding on estimates of medication-related harms after addressing known covariates that could have been potential confounders. Our results suggest that lack of blinding of participants and health care providers in randomized controlled trials may substantially influence estimates of medication-related harms, regardless of whether outcomes are objective or subjective. We found that, on average, lack of blinding was associated with underestimation of harm effects by 32%. These findings highlight the importance of blinding in randomized controlled trials for harmful outcomes, just as it is for efficacy outcomes. Nevertheless, blinding of trial assessors may have less or no influence on estimates of harms which are directly recorded by participants and health care personnel without requiring any additional input or adjustment by trial assessors.

There was a substantial difference in our findings from previous empirical investigations. In the study by Savovic in 2012, trials lacking blinding on participants and health care providers showed significantly exaggerated treatment effects (effectiveness/efficacy) in subjective outcomes, but not for objective outcomes [6]. In their further study in 2018, similar results were observed again [35]. The MetaBLIND study found no impact of lack of blinding on both subjective and objective efficacy outcomes [11]. However, in our study, evidence of the significant impact of blinding on both objective and subjective harm outcomes was observed. We postulate that for harm outcomes, lack of blinding on participants and care providers may be associated with performance bias [3], which would result in deviation of intended intervention, regardless of whether the outcome is objective or subjective.

The directed acyclic graphs (see Additional file 1: Fig. S1) may help us to further interpret our findings. There were several causal paths for blinded participants and/or health care providers on harms, namely, (1) the direct path and (2) via the interventions, controls, or dosage to influence harm (“indirect” paths). The current study focused on the direct effect of lack of blinding on the estimation of medication-related harms by restricting intervention, dosage, and control to be identical within meta-analysis, but it is still possible that the “indirect” paths partially explain the underestimation of harms due to lack of blinding. For example, for participants who did not adhere to the intervention or switched to another intervention when they were aware of the intervention they received, the intervention for them would be distorted and could influence assessment of harm effects. Similarly, it is possible that health care providers applied additional interventions to participants if they were aware of treatment assignment.

In the directed acyclic graphs, there is only one path for blinding trial outcome assessors to harm effects, namely, the direct effect. It may be anticipated that measurement of objective outcomes is dependent on outcome assessors, as no subjective judgment might be involved. For subjective outcomes, there was also no difference in harm effects between blinded and unblinded trial assessors. It is possible that blinding for outcome assessors may not have been applied for all outcomes; for example, blinding may have been applied only for efficacy outcomes, not for harm outcomes. In addition, many harm outcomes were patient-reported or reported by heath care providers (e.g., diarrhea) and blinding for other parties involved in trial outcome assessment (e.g., safety monitoring panel) may have played no role in such subjective outcomes. In such a situation, blinding of the safety panel may prevent further bias creeping into the data, but this blinding cannot easily remove bias that has already occurred earlier at source. Considering the differential impact of blinding on harm effects, further research is worthwhile to verify our findings and explore potential mechanism(s).

The findings of the current study have important implications for future evidence synthesis research. Currently, evidence synthesis researchers may not always give detailed consideration towards potential methodological weakness in harms reported in included trials, thus possibly ignoring the potential impact of such weaknesses on the validity of the final result. Based on the evidence of our current study, it would be sensible to carefully consider the potential impact that lack of blinding may have and perhaps effect estimates based on such components of methodological weaknesses should be treated as part of a sensitivity analysis to inform evidence users [36].

### Strengths and limitations

To the best of our knowledge, this is the first study that has investigated the influence of lack of blinding on estimation of harms. Our large-scale dataset ensures a sufficient number of “observations” to achieve a valid estimation of results. Data accuracy in this study has been checked multiple times, and the data collection process was also carefully recorded, thus providing greater safeguards against potential bias due to data errors or non-transparency. In the data analysis, we identified potential confounders and addressed them via harmonization procedures, in an effort to obtain the direct effect of lack of blinding on estimating harms. All of these steps serve to increase the robustness and reliability of our study findings.

Some limitations should be highlighted. First, due to the nature of the observational design of our study, we are unable to determine a causal relationship. Although we employed directed acyclic graphs to detect potential confounders, it is not possible to control for all confounders. Several unmeasured methodological issues could influence our results. For example, dropouts from randomized trials may result in missing data bias for harm effects. There is also a possibility that blinding could be compromised if trial participants or health care providers successfully guessed the study intervention, and this could further influence reporting or recording of harms. In our database, we identified 11 randomized controlled trials that reported the proportion of correct guesses of intervention allocation by participants or health care providers, with a proportion ranging from 10.6 to 85.7% (median: 59.0%) for intervention group and 31.9 to 78.4% for control group (median: 49.6%). Second, we were unable to account for the potential difference on the settings of the trials and the varying definitions of harms in the trials, as well as the biological nature of the harms, which may contribute to some amount of heterogeneity of the results [37–39]. Third, missing data may have had an impact on the results. Even though the missing rate was low for each variable used in the current study, when considered in total, missing data resulted in 23.60% study loss, which could impact the validity of our results. The integrity of such information largely relies on comprehensive reporting of the included trials, which is a parameter that can only be addressed through strict adherence to reporting guidelines. Fourth, poor reporting of harms may impact the representativeness of the current study, as empirical evidence showed that only 43% of published trials reported harms data [40]. The release of the new CONSORT Harms statement [41] is expected to be helpful in promoting harms reporting in future randomized trials.

## Conclusions

In summary, our study demonstrates that lack of blinding of participants and health care providers in randomized controlled trials may lead to underestimates of medication-related harm effects, regardless of whether these were objective or subjective outcomes. However, lack of blinding of trial outcome assessors may not necessarily influence estimates of harm effects. Implementing blinding in randomized trials, when feasible, may help safeguard against potential bias in estimating effects of harms.

### Supplementary Information

**Additional file 1: Fig. S1.** The process of moderator harmonization. **Fig. S2.** The DAG plot for identifying potential effect modifiers (Blind for participants and health care providers). **Fig. S3.** The DAG plot for identifying potential effect modifiers (Blind for outcome assessors). **Fig. S4.** The word cloud of harm outcomes of the SMART Safety dataset.

## Data Availability

Data can be found at https://osf.io/g3mdu/.

## Abbreviations

CI: Confidence interval
CONSROT: Consolidated Standards of Reporting Trials
IQR: Interquartile ranges
ITT: Intention-to-treat
OR: Odds ratio
PRISMA: Preferred Reporting Items for Systematic Reviews and Meta-Analyses
RoB: Risk of bias
ROR: Ratio of odds ratio
