# Supplementary material for: Influence of lack of blinding on the estimation of medication-related harms: a retrospective cohort study of randomized controlled trials
Source: BMC Med. 2024 Mar 7;22:83. doi: 10.1186/s12916-024-03300-7 (PMC10919027; doi:10.1186/s12916-024-03300-7)
Supplement: Supplementary file 1 — Additional file 1: Fig. S1. The process of moderator harmonization. Fig. S2. The DAG plot for identifying potential effect modifiers (Blind for participants and health care providers). Fig. S3. The DAG plot for identifying potential effect modifiers (Blind for outcome assessors). Fig. S4. The word cloud of harm outcomes of the SMART Safety dataset. [file 12916_2024_3300_MOESM1_ESM.docx]

**Protocol**

## Protocol (11 April 2021)

## Empirical investigation of potential bias for the harmful effects in randomized controlled trials

Chang Xu^1, 2^, Liliane Zorzela^3^, Luis Furuya-Kanamori^4^, Lifeng Lin^5^, Jiaxin Zhang^6^, Sunita Vohra^3,7^

1. Department of Population Medicine, College of Medicine, Qatar University, Al Jamiaa Street, P. O. Box 2713, Doha, Qatar;
2. Chinese Evidence-based Medicine Center, West China Hospital, Sichuan University, Chengdu, China;
3. Department of Pediatrics, Faculty of Medicine & Dentistry, University of Alberta, Edmonton, Alberta, Canada
4. UQ Centre for Clinical Research, Faculty of Medicine, University of Queensland, Brisbane, Australia
5. Department of Statistics, Florida State University, Tallahassee, FL, USA
6. Department of Pharmacy, Guizhou Provincial People's Hospital, Guiyang, China
7. Department of Psychiatry, Faculty of Medicine & Dentistry, University of Alberta, Edmonton, Alberta, Canada

## ^🖂^Correspondence to:

Dr. Chang Xu, Department of Population Medicine, College of Medicine, Qatar University, Al Jamiaa Street, P. O. Box 2713, Doha, Qatar; Email: [xuchang2016@runbox.com](mailto:xuchang2016@runbox.com)

## Research questions

Safety is as important as effectiveness in the assessment of health interventions. As highlighted by the latest Cochrane handbook (version 6.2), all systematic reviews of interventions should investigate the adverse effects of the intervention [1]. Rare adverse events pose a substantial challenge for statistical modelling and inference for safety assessment, for example, low statistical power, biased effect estimation [2]. There are many potential factors that cause events to be rare in single trials. These factors result in either random error or systematic error that may bias the estimation of treatment effects. For example, low incidence, small sample size, and limited treatment duration may lead to large random errors [3-6]; while selective non-reporting or industry funding may lead to large systematic errors that bias estimates of treatment effects [7-10].

One example can illustrate the above bias clearly. Suppose the true incidence rates (per month) of a certain adverse event (AE) are 0.0005 and 0.0001 for treatment A and treatment B, respectively. The expected risk ratio (RR) (A vs. B) is therefore 0.0005/0.0001=5. Now let’s suppose a randomized controlled trial (RCT), with the sample sizes 200 vs. 200, the treatment duration time is 1 month, and therefore the expected events in both arms are 200*0.0005*1=0.1, 200*0.0001*1=0.02; then the observed events could be both 0. Thus, the estimated “pseudo” RR is then 1 that is largely biased. However, if we extend the treatment duration time to 12 months, then the expected events are 0.1*12=1.2, 0.02*12=0.024, and therefore the observed events could be 1 and 0, with an estimated RR of 3 much closer to true effect. Now, if researchers do not report the 1 event as they think this may be unfavorable for their new drug, the “adjusted” RR then back to 1 and showed no difference of the risk.

Considering the substantial impact of these factors on the estimate of treatment effects and conclusions of safety assessment, we plan to employ a large-scale empirical investigation to see the extent of the impact and seek potential solutions to adjust the bias by these factors.

**Methods**

***Database***

We will use the database of our recent ongoing project that investigated the preference of systematic review authors in dealing with studies with no events in meta-analyses of adverse events. The database collected systematic reviews of healthcare intervention with safety as exclusive outcome indexed in PubMed from 1 Jan 2015 to 1 Jan 2020.

***Eligibility***

The following systematic reviews will be included:

1. Safety as the exclusive outcome;
2. Includes only clinical trials;
3. Contains meta-analysis which with at least 5 studies;
4. Any drug or biological agent as intervention, with a comparison of any active or non-active drugs drug, biological agent, or placebo;
5. Provided 2 by 2 table data for each study in the table or forest plot;

We plan to limit meta-analyses for at least 5 studies for two reasons. First, the comparison (e.g. for-profit funding vs. not-for-profit funding) will be based on matching (e.g. sample size, treatment duration, incidence), more studies in a meta-analysis mean a higher possibility for successful matching; Second, our simulation suggested that a meta-analysis with less than 5 studies is inconclusive.

We plan to limit intervention to drugs and biological agents since they are more likely to be funded by industry. Our requirement that original 2 by 2 table data for each study be provided is based on the consideration that most of the meta-analyses did not appropriately deal with zero-events studies, leading to further systematic error. Such systematic error will bias our comparison.

## Context

This study will focus on the potential impact of incidence, sample size, treatment duration time, non-reporting bias, and source of funding on harms reporting in trials, with a special focus on treatment duration time and source of funding. This will be conducted by comparing harms reported in trials with, for example, long treatment duration time/industry funding, to those with short treatment duration time/non- industry funding. Considering the potential impact of the other remaining factors (e.g. sample size, non-reporting bias), we plan to use the matching method whenever possible, say, when comparing the effects of industry-funded trials with non-profit institution funded trials, we will match the trials by baseline incidence, sample size, treatment duration, risk of bias. For risk of bias, the ROB 2.0 tool will be used [11]; for non-reporting bias, the Outcome Reporting Bias in Trials (ORBIT) for harm outcomes will be used [12, 13].

Therefore, thus information (i.e. 2 by 2 table, treatment duration time, risk of bias, source of funding) will be extracted for each trial in each eligible systematic review. In addition, baseline information like year of publication, original data for meta-analysis, related topics (e.g. cancer) will also be extracted.

Considering that some of the trials involve two periods, with different aims for each period, we extract the adverse events, treatment duration for each period separately. The information of whether the study or study period was double-blind, single-blind, or open-label will also be recorded.

## Additional analysis

Meta-regression analysis for those eligible meta-analyses with 10 or more studies will be conducted for treatment duration time (or other factors is possible).

## Main outcome(s)

- The proportion of data extraction errors by these systematic reviews.
- The main outcome is the relative odds ratio (ROR) of each matched pair of trials. In addition, the topic-specific pooled ROR based on the inverse variance heterogeneous (IVhet) model will be calculated [14]. Considering that the method for dealing with zero-events may impact the pair-specific ROR and further impact the pooled ROR, we only consider trials with 1:1 design or close to 1: 1 (i.e., ratio ranges from 0.51 to 1.99). Because under 1:1 design, the continuity correction (add 0.5) works well for both single-arm-zero-events and double-arm-zero-events.

## Secondary outcome(s)

- The proportion of single-arm-zero-events studies and double-arm-zero-events studies within a meta-analysis.
- The potential impact of treatment duration time, non-reporting bias, source of funding for the occurrence of zero-events.

The following information will also be of interest for each eligible meta-analysis that reflects what extent the harmful effects were investigated:

1. Whether the meta-analysis investigated the potential impact of different treatment/control on the harmful effects?
2. Whether the meta-analysis investigated the potential impact of treatment duration on the harmful effects?
3. Whether the meta-analysis investigated the potential impact of doses of drug on the harmful effects?
4. Whether the meta-analysis investigated the potential impact of funding sources on the harmful effects?
5. Whether the meta-analysis investigated the potential impact of risk of bias on the harmful effects?
6. Whether the meta-analysis investigated the potential impact of age on the harmful effects?
7. Whether the meta-analysis rank the confidence of the evidence of harmful effects?

## Data extraction (selection and coding)

Data extraction will be done by the lead author and several research assistants (see below the table of the records), and further checked by a research assistant. See “context” section for information to be exacted.

## Analysis of subgroups or subsets

Year of publication of trials, sample size, incidence.

## Contact details for further information

[xuchang2016@runbox.com](mailto:xuchang2016@runbox.com)

## Conflicts of interest

We declare no conflict of interest.

## Funding

This study did not receive any financial support.

Stage of review
Review Ongoing: Literature screen for the current study has not been done (By 11 April 2021).

## Search Strategy (PubMed, conducted on 28^th^-July, 2020)

1. "Systematic Reviews as Topic"[Mesh] OR "Systematic Review" [Publication Type] OR "Meta-Analysis as Topic"[Mesh] OR "Meta-Analysis" [Publication Type] OR "meta-analysis"[Title/Abstract] OR "meta analysis"[Title/Abstract] OR "systematic review"[Title/Abstract]
2. "randomized controlled trials as topic"[MeSH Major Topic] OR "clinical trials as topic"[MeSH Major Topic] OR "controlled clinical trials as topic"[MeSH Major Topic]
3. "randomized controlled trial*"[Title/Abstract] OR "controlled clinical trial*"[Title/Abstract] OR "clinical trial*"[Title/Abstract] OR "controlled trial*"[Title/Abstract] OR "trial*"[Title/Abstract]
4. "safety"[Title/Abstract] OR "harm*"[Title/Abstract] OR safe[Title/Abstract] OR poisoning[Title/Abstract] OR toxicity[Title/Abstract] OR tolerability[Title/Abstract] OR "complication*"[Title/Abstract] OR "adverse event*"[Title/Abstract] OR "adverse outcome*"[Title/Abstract] OR "untoward effect*"[Title/Abstract] OR "side effect*"[Title/Abstract] OR adverse n2 reaction[Title/Abstract]
5. #2 or #3
6. #1 AND #4 AND #5
7. Protocol[Title] OR overview [Title] OR "narrative review" [Title]
8. #6 NOT #7
9. (#8) AND (("2018/01/02"[Date - Publication]: "2020/01/01"[Date - Publication])) AND (humans[Filter]) Filters: Humans
10. (#8) AND (("2015/01/01"[Date - Publication]: "2018/01/01"[Date - Publication])) AND (humans[Filter]) Filters: Humans
11. #9 or # 10

**References**

1. Peryer G, Golder S, Junqueira DR, Vohra S, Loke YK; on behalf of the Cochrane Adverse Effects Methods Group. Chapter 19: Adverse effects. Cochrane Handbook for Systematic Reviews of Interventions version 6.2 (updated February 2021). Cochrane, 2021. Available from [www.training.cochrane.org/handbook](http://www.training.cochrane.org/handbook).
2. Jia P, Lin L, Kwong JSW, Xu C. Many meta-analyses of rare events in the Cochrane Database of Systematic Reviews were underpowered. J Clin Epidemiol. 2021;131: 113-122.
3. Xu C, Li L, Lin L, Chu H, Thabane L, Zou K, Sun X. Exclusion of studies with no events in both arms in meta-analysis impacted the conclusions. J Clin Epidemiol. 2020;123: 91-99.
4. Ju K, Lin L, Chu H, Cheng LL, Xu C. Laplace approximation, penalized quasi-likelihood, and adaptive Gauss-Hermite quadrature for generalized linear mixed models: towards meta-analysis of binary outcome with sparse data. BMC Med Res Methodol. 2020;20(1):152.
5. Xu C, Furuya-Kanamori L, Zorzela L, Lin L, Vohra S. A proposed framework to guide evidence synthesis practice for meta-analysis with zero-events studies. J Clin Epidemiol. 2021; 135:70-78.
6. Zorzela L, Loke YK, Ioannidis JP, Golder S, Santaguida P, Altman DG, et al. PRISMA harms checklist: improving harms reporting in systematic reviews. BMJ. 2016;352: i157.
7. Aylin P, Tanna S, Bottle A, Jarman B. How often are adverse events reported in English hospital statistics? BMJ. 2004; 329(7462):369.
8. Janiaud P, Cristea IA, Ioannidis JPA. Industry-funded versus non-profit-funded critical care research: a meta-epidemiological overview. Intensive Care Med. 2018; 44(10):1613-1627.
9. Ioannidis JP, Lau J. Completeness of safety reporting in randomized trials: an evaluation of 7 medical areas. JAMA. 2001;285(4):437-43.
10. Als-Nielsen B, Chen W, Gluud C, Kjaergard LL. Association of funding and conclusions in randomized drug trials: a reflection of treatment effect or adverse events? JAMA. 2003; 290(7):921-8.
11. Higgins JPT, Savović J, Page MJ, et al. Revised Cochrane risk of bias tool for randomized trials (RoB 2.0), Version 20. 2016 <https://sites>. google.com/site/riskofbiastool/ (assessed at 18-April, 2021)
12. Saini P, Loke YK, Gamble C, Altman DG, Williamson PR, Kirkham JJ. Selective reporting bias of harm outcomes within studies: findings from a cohort of systematic reviews. BMJ. 2014 Nov 21;349:g6501.
13. ORBIT classifications (harm outcomes). http://outcome-reporting-bias.org/HarmOutcomes
14. Doi SA, Barendregt JJ, Khan S, Thalib L, Williams GM. Advances in the meta-analysis of heterogeneous clinical trials I: The inverse variance heterogeneity model. Contemp Clin Trials. 2015 Nov;45(Pt A):130-8.

**Appendix Table S1. Records of data collection.**

| 11-April, 2021 | Draft the protocol |
| --- | --- |
| 14-April, 2021 | Changing “follow-up time” to “treatment time” after consulting experts of clinical trials.  **Reason:** Any treatment has wash-out period, after the period, any adverse events may not be caused by intervention. |
| 17-April, 2021 | Send for collaborators for reviewing, no context changes |
| 22-April, 2021 | Distinguishing the treatment duration and control duration in data extraction form based on extraction training |
| 25-April, 2021 | Adding more information in secondary outcome, say, the seven “Whether…”, as an effort to see how well the harmful effects were investigated |
| 4-May, 2021 | Change inclusion criteria: meta-analysis of RCTs to meta-analysis of clinical trials.  Reason: Some meta-analyses claimed they included phrase II or II trials, and did not mention whether these were RCTs or not. Based on our experience, the majority were RCTs. Therefore, we expand the inclusion criteria from RCTs to clinical trials. |
| 15-May, 2021 | During the data extraction, we noticed many of the systematic reviews failed to report or conduct the risk of bias of each trial. After an online meeting, we decided to assess the risk of bias by our research team. |
| 17-May, 2021 | Two assistants were added to the research, with a background in evidence-based medicine. They take charge of the assessment of risk of bias. Since the assessment of ROB is somewhat subjective, the two assistants are blinded; they were not and will not be informed of the aim of the project and the potential comparisons. And the assessment of risk of bias will be started after the finish of the data extraction (possibly in July). |
| 28-May, 2021 | Change in the analysis of the main outcomes: We primarily plan to limit RCTs with 1:1 design to facilitate the estimation of the ORs in the case of zero-events. But then after our recent simulation study, we found continuity correction works well even when the ratio ranges from 0.51 to 1.99, therefore we “relaxed” the limits to 0.51 to 1.99. |
| 1-June, 2021 | We primarily collected data of the clinical trials from the systematic reviews, while for the sample we finished (which accounted for 1/3 of the total), we found that many of the data were incorrectly recorded by these systematic reviews. Based on an urgent online meeting (CX and TQ), we decided to re-do the data extraction, directly from the original studies. |
| 5-June, 2021 | Online meeting with a pharmacist about the definition of different treatments/controls. |
| 10-June, 2021 | Some clinical trials may have a flexible treatment schedule, for example, they use A drug with 5 mg at week 1, and 10 mg at week 2, and 20 mg at week 3, and then continue 20 mg for the following 11 weeks.  Makes it difficult to extract the dose. After a discussion (CX and TQ), we decided to use the maximum dose in our study. |
| 14-June, 2021 | Add the tools that will be used for ROB assessment. |
| 15-June, 2021 | Add information for extraction: whether the trials involve two periods, and the design (double/single blind, open label) of each period. |
| 26-June, 2021 | Two assistants (WJ, FY) are ready to start the assessment of the ROB. An online meeting is held to discuss the use of ROB 2 (XC, TQ, WJ, FY). Prior training for the two assistants based on 5 trials is prepared and will start tomorrow. The second online meeting will be held after the training for a further discussion of any issues during the preliminary assessment.  **Note:** We finished about 50% of the systematic reviews for the intended data collection, and the included trials of these systematic reviews are ready for the ROB assessment. For the rest 50%, we may finish it 3 weeks later, and as long as we finish, we will prepare the trials included in these reviews for WJ and FY for the ROB assessment. |
| 30-June, 2021 | The two assistants finished the first period training of 2 RCTs; there were many disagreements between them on the judgement of each item. Thus, an online meeting is held discussion the issues of the assessment of each domain, each item, by reading the explanation of the ROB 2. After 1 hour’s discussion, an agreement is achieved of the assessment criteria. And the two assistants will continue to assess the remaining 3 RCTs for training. |
| 2-July, 2021 | The two assistants finished the remaining 3 RCTs, and an online meeting is held to discuss further issues they met during the assessment. There is a high agreement on the judgement of the items this time, and only 3 minor points are involved. The training is now finished, and they are ready to formally start the assessment of the ROB. |
| 15-July, 2021 | During the data extraction of the meta-analysis data from original RCTs, we recorded many pieces of incorrect information from these meta-analyses. We then decided to treat this as an additional project that investigated how many meta-analyses with the data were incorrect. In this additional project, we will add systematic reviews other than drug/biologics treatment in order to increase the representativeness of the dataset. Both the data obtained from the meta-analyses and the data obtained from original RCTs will be recorded. We will first investigate the proportions that systematic reviews failed to extract the correct 2 x 2 table data; we will also record the type of incorrectness; we will further compare the meta-analysis based on correct data to the incorrect one to see how the incorrection impacts the results. Another assistant (Z.XQ) will take charge of the data extraction for systematic reviews other than drug/biologics treatments. The data will then be double-checked by the assistant after she finishes it. |
| 15-July, 2021 | XC and DMH finished the data extraction of SRs assigned to them, and started double-checking the data they extracted. |
| 17-July, 2021 | Until now, XC checked for 571 rows of 2 by 2 table data he extracted from RCTs (with 28 meta-analyses), and found 21 rows were incorrect by himself. The main reason is that RCTs present the same outcome for several times, thus leading to confusion (e.g. study by List et al. Diabetes Care. 2009 Apr;32(4):650-7. Table 2. Urinary tract infection). The estimated proportion of mis-extraction by himself is then 21/571=3.7%. Suggests a high accuracy of the data extraction. However, the mis-extraction also suggests the importance of double-checking process. Therefore, an online meeting (XC, YTQ, and DMH) is then held by XC, and everybody is required to check each cell of the 2 by 2 table very carefully to ensure 0% error. In addition, YTQ and DMH are required to report their own accuracy to XC during their double-checking process. |
| 18-July, 2021 | Until now, XC checked for 696 rows of 2 by 2 table data he extracted from RCTs (with 43 meta-analyses), and found 24 rows were incorrect by himself. The estimated proportion of mis-extraction by himself is then 24/696=3.4%. The accuracy is better for the data extracted more recently (3.7% vs. 2.4% [3/125]). |
| 20-July, 2021 | Until now, XC checked for 850 rows of 2 by 2 table data he extracted from RCTs (with 54 meta-analyses), and found 24 rows (no errors from 697-850) were incorrect by himself. The accuracy is 24/850=2.8%. Again, the accuracy is much better for the data extracted more recently (3.7% vs. 2.4% vs. 0%). |
| 20-July, 2021 | Z.XQ finished the meta-analytic data (2 by 2 table for each included study) extraction from the forest plot/tables of the meta-analyses other than drug/biologics. She will start the 2 by 2 table data extraction from original RCTs thereafter, and she was required to double-check the data she extracted from meta-analysis and report her accuracy to XC. |
| 20-July, 2021 | Report by DHM: From rows 2 to 519 and row 1068 to 1310, there were 5 to 10 errors (she estimated) in the data extracted by DHM. The proportion ranges from 5/760 (0.66%) to 10/760 (1.32%). |
| 21-July, 2021 | Report by XC: From rows 850 to 1073, there were 12 errors in the data extracted by XC, with a proportion of 12/223=5.38%. This is because he put the data of “any infection” into “serious infection” while the data of “serious infection” into “any infection” in Silvia 2016’s review. |
| 22-July, 2021 | Report by XC: From rows 1073 to 1678, there were 4 typo errors in the data extracted by XC, with a proportion of 4/605=0.66%. |
| 22-July, 2021 | XC starts to extract the data of the remaining 25 systematic reviews assigned to him. |
| 27-July, 2021 | DHM starts to extract the data of the remaining 15 systematic reviews assigned to her. |
| 30-July, 2021 | Report by XQ: From rows 1 to 145, there was 1 typo error in the data extracted by her, with a proportion of 1/145=0.69%. |
| 30-July, 2021 | TQ starts double-checking for the data she extracted. |
| 5-Aug, 2021 | XC has finished the data extraction of the remaining 25 systematic reviews with 86 meta-analyses assigned to him. He plans to check the data thereafter. |
| 7-Aug, 2021 | DHM has finished the data extraction of the remaining 14 systematic reviews assigned to her. And she plans to check the data thereafter. |
| 7-Aug, 2021 | Report by TQ: Until now, by checking the data she extracted, the proportions of errors were 4 from rows 1 to 373 (4/373=1.07%), 6 from rows 374 to 799 (6/425=1.41%), 4 from rows 800 to 1151 (4/351=1.14%), 5 from rows 1151 to 2351 (5/1200=0.42%), and 1 from rows 2352 to 3435 (1/1083=0.093%). |
| 7-Aug, 2021 | TQ starts to extract the data of the remaining 16 systematic reviews assigned to her. |
| 23-Aug, 2021 | DHM finished all the SRs assigned to her. |
| 23-Aug, 2021 | Report by DHM: For the remaining 14 SRs with 68 meta-analyses, there were about 1-5 errors by herself based on her double-checking. The proportion of errors ranged from 1/1023=0.08% to 5/1203=0.49%. |
| 26-Aug, 2021 | Report by XC: For 14 SRs of 39 meta-analyses he double-checked until today, there were 4 errors from 647 rows, with a proportion of 4/647=0.62%. There remain 11 SRs to be double-checked. |
| 27-Aug, 2021 | TQ finished the data extraction of the 16 SRs assigned to her, and she will start the double-checking process thereafter. |
| 30-Aug, 2021 | Report by XC: For the remaining 11 SRs with 47 meta-analyses, there were 4 errors among 609 rows, with a proportion of 4/609=0.66%. Two for typo errors, and two (one study) for mistakes in the definition of outcome. |
| 2-Sep, 2021 | Report by TQ: For the remaining 16 SRs with 59 meta-analyses, there were 6 errors by herself among 1325 rows, with a proportion of self-error of 6/1325=0.45%, mainly due to typo errors as well as failing to find the outcomes in the first time. |
| 19-Sep, 2021 | ZXQ finished the data extraction for the 40 SRs based on the original RCTs. |
| 19-Sep, 2021 | Report by ZXQ: From the 40 SRs with 2495 rows, there were 22 errors in total by herself, with a proportion of self-error of 22/2495 = 0.88%. |
| 26-Sep, 2021 | Data cleaning and checking were finished by the principal author, assisted by the three assistants. |
| 7-Oct, 2021 | A fourth-round checking was finished by the principal author, focusing on the information of subgroup analysis, those identified with data extraction errors, type of errors classification, and those without full-text recorded by the four assistants. A few minor typos/confusions were addressed. |
| 16-Oct, 2021 | A fifth-round checking by TQ finished, which mainly focused on the coding of the ID of systematic review, ID of each meta-analysis, information of subgroups, and eligibility of all systematic reviews. TQ found that 9 systematic reviews should not be excluded and need further data extraction. |
| 17-Oct, 2021 | An online meeting by XC and TQ for the 9 additional systematic reviews, and decided to extract the data by one of the assistants that do not know the summarized results. |
| 18-Oct, 2021 | Hanmin started data extraction for the 9 additional systematic reviews. |
| 22-Oct, 2021 | Hanmin finished the data extraction of the addition systematic reviews. And XC started double-checking for the data. |
| 25-Oct, 2021 | XC finished the double-checking of the data. As such, all the data extractions were finished. |
| 26-Aug, 2021  **(ROB and checking)** | One of the assistants who took charge of the ROB assessment (MW) decided to withdraw from the research group, after 2 months of his participating. The assessment of ROB remains one assistant. The project leader started to recruiting new assistant to continue the assessment of ROB. |
| 13-Jan, 2022 | TQ and YY took over the ROB assessment. Considering the large workload, the project leader decided to recruit more assistant, and arrange TQ, FY, and YY as group 1 for ROB assessment, with each take charge of about one-third of the trials. |
| 11-Feb, 2022 | FY finished the ROB assessment of her parts. In the point of quality control, the lead author requires FY to re-check the data for ROB assessment. |
| 10-Mar, 2022 | TQ finished the ROB assessment of her parts. In the point of quality control, the lead author requires TQ to re-check the data for ROB assessment. |
| 11-May, 2022 | FY finished the self-checking of the ROB of her parts. |
| 20-June, 2022 | YY finished the ROB assessment of her parts. And now, group 1 finished all the ROB assessments. In the point of quality control, the lead author requires YY to re-check the data for ROB assessment. |
| 21-Aug, 2022 | YY finished the self-checking of the ROB of her parts. |
| 27-Nov, 2022 | TQ finished the self-checking of the ROB of her parts. |
| 17-Jan, 2023 | YX and RPW participant in the research as ROB assessors for Group 2. A pilot training by 5 randomized trials was assigned to them. |
| 25-Jan, 2023 | YX and RPW finished the training, and started the ROB assessment (blinded to Group 1). |
| 11-Feb, 2023 | RPW withdrawal from the research group. And YX takes charge of all the ROB assessment of Group 2. |
| 12-Mar, 2023 | Two undergraduate students (XX and TY) join in the research team; after a pilot training, they were asked to mark the dose information, namely, marked studies with the same dose within each meta-analysis using consecutive numbers. For example, if two studies had the same dose in a meta-analysis, they will be marked with the same number. This process is designed to facilitate the following matching process, and will be done separately by the two students. |
| 23-Mar, 2023 | The two students finished the marking task for dose information. |
| 24-Mar, 2023 | Under the supervise by YX, the two students cross-check their marked results and discuss any disagreements. |
| 24-Mar, 2023 | The discussion meeting by YX, XX, TY found some studies with dose information were ambiguously extracted which make it difficult to determine the dose. After discussed with project leader XC, we decided to check the dose information, and then re-mark the number.  As we already have the dose information, the checking process will be done by one student (TY), and finally will be checked again by project leader XC. |
| 13-April, 2023 | YX finished the ROB assessment. |
| 13-April, 2023 | ZR compared the ROB information assessed by Group 1 and Group 2, and marked the RCTs with inconsistent information.  YX and ZR discuss the disagreements and re-check the information from related trails. |
| 16-April, 2023 | ZR and YX finished the double-checking of the ROB, and delivered the data to the project leader XC. XC and ZR started to check any missing RCTs, and detected 357 had missing ROB information (in both groups). After carefully checking of the dataset, XC confirmed that the missing information was due to the process of removing duplicates and a failing to record the citation form of some RCTs. |
| 16-April, 2023 | ZR and YX started to assess the ROB of the remaining 357 RCTs, separately. |
| 17-Apri, 2023 | Of the 357 RCTs, ZR and YX obtained full-texts of 318 RCTs.  For the remaining 39, CX re-checked the assess of full texts. For these, 10 were identified as conference abstracts, with 2 of which were duplicates. For the remaining 29, CX was able to obtain the full-texts of 14 RCTs. For the rest 16, the full-texts were failed to obtain through many attempts (e.g., asking for help).  Therefore, 332 RCTs were to be assessed of the ROB, separately, by ZR and YX. Based on previous experience, about 10 to 20 mins for each RCT of the ROB, thus they planned 50 RCTs per day. |
| 20-April, 2023 | Report from ZR: 200 RCTs were finished the ROB assessment.  Report from YX: 200 RCTs were finished the ROB assessment. |
| 23-April, 2023 | ZR and YX finished the ROB assessment.  XC started to check the agreements and disagreements of their information.  The marked disagreements were discussed by ZR and YX, based on re-checking of the related RCTs. |
| 24-April, 2023 | A study (Gen Dent, 2013, 61: 70-6) without full-text was obtained through internet inquiry and was further identified as non-RCT. |
| 24-April, 2023 | ZR and YX finished the double-checking process and reached a consensus on all RCTs of the ROB information. |
| 24-April, 2023 | Report from TY: of the 10,069 lines, by now, he has finished 7,237 of the dose information checking and correction. And for the dose information, from line 1 to line 5386 (from the first to 363 meta-analyses) dose information was well extracted. While after then, the dose information was not well extracted. TY expected he would finish all the checking and correction by the next two days.  Note: This is because the total information was extracted by four authors separately, and the information of the first 363 meta-analyses was extracted by the lead author (XC) and DHM. The rest were extracted by TQ, where the dose information was ambiguous. |
| 25-April, 2023 | Considering the dose information was well extracted for the first 363 meta-analyses, XC checked the marked information of the first 363 meta-analyses by XX and TY for the most update data.  For the 5,386 lines, XC detected 131 lines with errors; the error rate was 2.43% (131/5386). All the detected errors were corrected. |
| 26-April, 2023 | Report from TY and XH: the rest 2832 lines of the dose information were checked and updated.  XC starts to re-check the dose information and the marked of the dose information (dose indicator). |
| 27-April, 2023 | Report from XC: From lines 5387 to 6305, which refers to the 364 to 390 meta-analyses, there were 120 errors in dose indicator, with an error rate of 13.1% (120/919). This is because the original dose information of this part and thereafter (extracted by TQ) lack of sufficient information that led to a wrong judgement on dose indicator (The information has been further checked and added by TY). |
| 28-April, 2023 | Report from XC: From lines 6306 to 7755, which refers to the 391 to 481 meta-analyses, there were 148 errors in the dose indicator, with an error rate of 10.21% (148/1450), with the same reason below recorded. |
| 29-April, 2023 | Report from XC: From line 7756 to line 10069, which refers to the 482 to 629 meta-analyses, there were 287 errors in the dose indicator, with an error rate of 12.40% (287/2314). Tile new, XC finished the triple checking of the dose indicator information.  In total, from line 5387 to line 10069, which refers to the 364 to 629 meta-analyses, there were 555 errors in the dose indicator, with an error rate of 11.85% (555/4683). |

**Appendix Table S2. Contribution of the dataset and quality control**

| **Information** | **Contributors** | **Comparison (Blind)** | **Double checking** | **Triple checking** | **Quadruple checking** |
| --- | --- | --- | --- | --- | --- |
| **Original information extracted from systematic reviews, randomized trials, and registration platforms** | | | | | |
| ***Aggregated data***  (events, group size) | Chang Xu, Tianqi Yu, Xiaoqin Zhou, Hanming Dai | NA  (Self-Checking) | Tianqi Yu | Chang Xu | Xi Yang |
| ***Treatment duration*** (Median) | Chang Xu, Tianqi Yu, Xiaoqin Zhou, Hanming Dai | NA | Chang Xu, Tianqi Yu | NA | NA |
| ***Funding*** | Chang Xu, Tianqi Yu, Xiaoqin Zhou, Hanming Dai | NA | Chang Xu | NA | NA |
| ***Registration*** | Yueyuan You | NA | Fengying Zhang | NA | NA |
| ***Results posting*** | Yueyuan You | NA | Fengying Zhang | NA | NA |
| ***Interventions***  (Two arms) | Chang Xu, Tianqi Yu, Xiaoqin Zhou, Hanming Dai | NA | Xi Yang | Rui Zhang | NA |
| ***Dose*** | Chang Xu, Tianqi Yu, Xiaoqin Zhou, Hanming Dai | NA | Yuan Tian, Xing Xing | Yuan Tian, Xing Xing, Xi Yang | Chang Xu |
| ***ITT/PP*** | Xi Yang | NA | NA | NA | NA |
| ***Age*** | Xi Yang | NA | NA | NA | NA |
| ***Risk of bias*** | **Group 1:** Fengying Zhang, Yueyuan You, Tianqi Yu  **Group 2:** Xi Yang  (Group 1 and Group 2 were independent for assessment) | Rui Zhang | Xi Yang, Rui Zhang | Xi Yang, Rui Zhang | NA |
| ***Center and Region*** | Fengying Zhang, Yi Zhu | NA | Fengying Zhang | NA | NA |
| ***DOI*** | Chang Xu, Tianqi Yu, Xiaoqin Zhou, Hanming Dai | NA | Fengying Zhang | NA | NA |
| **Generated information based on original information** | | | | | |
| ***SRID, MAID*** | Tianqi Yu | NA | Chang Xu | Chang Xu | Chang Xu |
| ***Outcome type*** (objective/composite) | Chang Xu, Luis Furuya-Kanamori  (Blinded) | Rui Zhang | Chang Xu, Luis Furuya-Kanamori | NA | NA |
| ***Error and Error Type*** | Chang Xu, Tianqi Yu | NA | Chang Xu, Tianqi Yu | NA | NA |
| ***Subgroup*** | Chang Xu, Tianqi Yu | NA | Chang Xu, Tianqi Yu | Xi Yang | NA |
| ***Assessable of Full-text*** | Chang Xu | NA | Rui Zhang | NA | NA |
| ***Net Interventions*** | Xi Yang | NA | Rui Zhang | NA | NA |

**All contributors:** Chang Xu, Tianqi Yu, Xi Yang, Fengying Zhang, Hanming Dai, Rui Zhang, Yueyuan You, Yuan Tian, Xing Xing, Xiaoqin Zhou, Luis Furuya-Kanamori, Cuncun Lu, Ke Ju, Yi Zhu

**Appendix Table S3. Missing data information of SMART Safety dataset.**

| **Variables with missing data** | **Missing counts** | **Proportions** |
| --- | --- | --- |
| Events in treatment group (r1) | 774 | 7.69% |
| Events in control group (r2) | 792 | 7.87% |
| Group size in intervention group (n1) | 106 | 1.05% |
| Group size in control group (n2) | 121 | 1.20% |
| Treatment duration in intervention group (t1) | 1,367 | 13.58% |
| Treatment duration in control group (t2) | 1,436 | 14.26% |
| Dose of net intervention | 479 | 4.76% |
| Interventions | 310 | 3.08% |
| Controls | 396 | 3.93% |
| Age (child, adult, eld) | 369 | 3.66% |
| Concealment | 471 | 4.68% |
| Blind for participants | 471 | 4.68% |
| Blind for care provider | 471 | 4.68% |
| Blind for outcome assessor | 471 | 4.68% |
| Access of full-text | 471 | 4.68% |
| Center information | 1,828 | 18.15% |
| Region information | 2,773 | 27.54% |
| Funding | 781 | 7.76% |
| Registry | 179 | 1.78% |

**Systematic reviews eligible for analysis (n = 151)**

1. Abdel-Qadir H, Ethier JL, Lee DS, Thavendiranathan P, Amir E. Cardiovascular toxicity of angiogenesis inhibitors in treatment of malignancy: A systematic review and meta-analysis. Cancer Treat Rev. 2017 Feb; 53:120-127.
2. Abdel-Rahman O, ElHalawani H, Essam-Eldin S. S-1-based regimens and the risk of leucopenic complications; a Meta-analysis with comparison to other fluoropyrimidines and non-fluoropyrimidines. Expert Opin Drug Saf. 2016;15(4):437-48.
3. Abdel-Rahman O, ElHalawani H, Fouad M. Risk of endocrine complications in cancer patients treated with immune check point inhibitors: a meta-analysis. Future Oncol. 2016 Feb;12(3):413-25.
4. Abdel-Rahman O, ElHalawani H, Fouad M. Risk of gastrointestinal complications in cancer patients treated with immune checkpoint inhibitors: a meta-analysis. Immunotherapy. 2015;7(11):1213-27.
5. Abdel-Rahman O, ElHalawani H. Risk of cardiovascular adverse events in patients with solid tumors treated with ramucirumab: A meta analysis and summary of other VEGF targeted agents. Crit Rev Oncol Hematol. 2016 Jun;102:89-100.
6. Abdel-Rahman O, ElHalawani H. Risk of hematological toxicities in patients with solid tumors treated with ramucirumab: a meta-analysis. Future Oncol. 2015;11(21):2949-61.
7. Abdel-Rahman O, Fouad M. Risk of mucocutaneous toxicities in patients with solid tumors treated with lapatinib: a systematic review and meta-analysis. Curr Med Res Opin. 2015 May;31(5):975-86.
8. Abdel-Rahman O, Fouad M. Risk of oral and gastrointestinal mucosal injury in patients with solid tumors treated with everolimus, temsirolimus or ridaforolimus: a comparative systematic review and meta-analysis. Expert Rev Anticancer Ther. 2015;15(7):847-58.
9. Abdel-Rahman O, Helbling D, Schmidt J, Petrausch U, Giryes A, Mehrabi A, Schöb O, Mannhart M, Zidan A, Oweira H. Treatment-associated Fatigue in Cancer Patients Treated with Immune Checkpoint Inhibitors; a Systematic Review and Meta-analysis. Clin Oncol (R Coll Radiol). 2016 Oct;28(10): e127-38.
10. Ammann EM, Haskins CB, Fillman KM, Ritter RL, Gu X, Winiecki SK, Carnahan RM, Torner JC, Fireman BH, Jones MP, Chrischilles EA. Intravenous immune globulin and thromboembolic adverse events: A systematic review and meta-analysis of RCTs. Am J Hematol. 2016 Jun;91(6):594-605.
11. Baxi S, Yang A, Gennarelli RL, Khan N, Wang Z, Boyce L, Korenstein D. Immune-related adverse events for anti-PD-1 and anti-PD-L1 drugs: systematic review and meta-analysis. BMJ. 2018 Mar 14;360:k793.
12. Bilal J, Berlinberg A, Riaz IB, Faridi W, Bhattacharjee S, Ortega G, Murad MH, Wang Z, Prokop LJ, Alhifany AA, Kwoh CK. Risk of Infections and Cancer in Patients With Rheumatologic Diseases Receiving Interleukin Inhibitors: A Systematic Review and Meta-analysis. JAMA Netw Open. 2019 Oct 2;2(10):e1913102.
13. Boltman-Binkowski H. A systematic review: Are herbal and homeopathic remedies used during pregnancy safe? Curationis. 2016 Apr 13;39(1):1514.
14. Bolton M, Hodkinson A, Boda S, Mould A, Panagioti M, Rhodes S, Riste L, van Marwijk H. Serious adverse events reported in placebo randomised controlled trials of oral naltrexone: a systematic review and meta-analysis. BMC Med. 2019 Jan 15;17(1):10.
15. Bonovas S, Minozzi S, Lytras T, González-Lorenzo M, Pecoraro V, Colombo S, Polloni I, Moja L, Cinquini M, Marino V, Goletti D, Matucci A, Tocci G, Milano GM, Scarpa R, Cantini F. Risk of malignancies using anti-TNF agents in rheumatoid arthritis, psoriatic arthritis, and ankylosing spondylitis: a systematic review and meta-analysis. Expert Opin Drug Saf. 2016 Dec;15(sup1):35-54.
16. Botero Aguirre JP, Restrepo Hamid AM. Amphotericin B deoxycholate versus liposomal amphotericin B: effects on kidney function. Cochrane Database Syst Rev. 2015 Nov 23;(11):CD010481.
17. Bundhun PK, Bhurtu A, Pursun M, Soogund MZS, Teeluck AR, Huang WQ. Long-term (2-5 years) adverse clinical outcomes associated with ZES versus SES, PES and EES: A Meta-Analysis. Sci Rep. 2017 Jul 25;7(1):6385.
18. Cai Q, Feng L, Yap KZ. Systematic review and meta-analysis of reported adverse events of long-term intranasal oxytocin treatment for autism spectrum disorder. Psychiatry Clin Neurosci. 2018 Mar;72(3):140-151.
19. Caldeira D, Alves D, Costa J, Ferreira JJ, Pinto FJ. Ibrutinib increases the risk of hypertension and atrial fibrillation: Systematic review and meta-analysis. PLoS One. 2019 Feb 20;14(2):e0211228.
20. Caldeira D, Barra M, Ferreira A, Rocha A, Augusto A, Pinto FJ, Costa J, Ferreira JJ. Systematic review with meta-analysis: the risk of major gastrointestinal bleeding with non-vitamin K antagonist oral anticoagulants. Aliment Pharmacol Ther. 2015 Dec;42(11-12):1239-49.
21. Caldeira D, Barra M, Santos AT, de Abreu D, Costa J, Ferreira JJ. Risk of insomnia with non-vitamin K oral anticoagulants: systematic review and meta-analysis. Sleep Breath. 2015 Sep;19(3):1043-9.
22. Caldeira D, Canastro M, Barra M, Ferreira A, Costa J, Pinto FJ, Ferreira JJ. Risk of Substantial Intraocular Bleeding With Novel Oral Anticoagulants: Systematic Review and Meta-analysis. JAMA Ophthalmol. 2015 Jul;133(7):834-9.
23. Caldeira D, Rodrigues FB, Barra M, Santos AT, de Abreu D, Gonçalves N, Pinto FJ, Ferreira JJ, Costa J. Non-vitamin K antagonist oral anticoagulants and major bleeding-related fatality in patients with atrial fibrillation and venous thromboembolism: a systematic review and meta-analysis. Heart. 2015 Aug;101(15):1204-11.
24. Caldeira D, Rodrigues FB, Duarte MM, Sterrantino C, Barra M, Gonçalves N, Pinto FJ, Ferreira JJ, Costa J. Cardiac Harms of Sofosbuvir: Systematic Review and Meta-Analysis. Drug Saf. 2018 Jan;41(1):77-86.
25. Capogrosso Sansone A, Mantarro S, Tuccori M, Ruggiero E, Montagnani S, Convertino I, Marino A, Fornai M, Antonioli L, Corona T, Garibaldi D, Blandizzi C. Safety Profile of Certolizumab Pegol in Patients with Immune-Mediated Inflammatory Diseases: A Systematic Review and Meta-Analysis. Drug Saf. 2015 Oct;38(10):869-88.
26. Catalá-López F, Corrales I, de la Fuente-Honrubia C, González-Bermejo D, Martín-Serrano G, Montero D, Saint-Gerons DM. Risk of thromboembolism with thrombopoietin receptor agonists in adult patients with thrombocytopenia: Systematic review and meta-analysis of randomized controlled trials. Med Clin (Barc). 2015 Dec 21;145(12):511-9.
27. Cates CJ, Schmidt S, Ferrer M, Sayer B, Waterson S. Inhaled steroids with and without regular salmeterol for asthma: serious adverse events. Cochrane Database Syst Rev. 2018 Dec 3;12(12):CD006922.
28. Chen F, Pu F. Safety of Denosumab Versus Zoledronic Acid in Patients with Bone Metastases: A Meta-Analysis of Randomized Controlled Trials. Oncol Res Treat. 2016;39(7-8):453-9.
29. Chen J, Lu Y, Zheng Y. Incidence and risk of hypertension with bevacizumab in non-small-cell lung cancer patients: a meta-analysis of randomized controlled trials. Drug Des Devel Ther. 2015 Aug 18;9:4751-60.
30. Ciccarese C, Iacovelli R, Bria E, Modena A, Massari F, Brunelli M, Fantinel E, Bimbatti D, Zamboni GA, Artibani W, Tortora G. The incidence and relative risk of pulmonary toxicity in patients treated with anti-PD1/PD-L1 therapy for solid tumors: a meta-analysis of current studies. Immunotherapy. 2017 Jun;9(7):579-587.
31. Coelho PL, da Silva Calestini GL, Alvo FS, de Moura Freitas JM, Castro PM, Konstantyner T. Segurança da vacina papillomavirus humano 6, 11, 16 e 18 (recombinante): revisão sistemática e metanálise [Safety of human papillomavirus 6, 11, 16 and 18 (recombinant): systematic review and meta-analysis]. Rev Paul Pediatr. 2015 Dec;33(4):474-82.
32. Conway R, Low C, Coughlan RJ, O'Donnell MJ, Carey JJ. Leflunomide Use and Risk of Lung Disease in Rheumatoid Arthritis: A Systematic Literature Review and Metaanalysis of Randomized Controlled Trials. J Rheumatol. 2016 May;43(5):855-60.
33. Conway R, Low C, Coughlan RJ, O'Donnell MJ, Carey JJ. Methotrexate use and risk of lung disease in psoriasis, psoriatic arthritis, and inflammatory bowel disease: systematic literature review and meta-analysis of randomised controlled trials. BMJ. 2015 Mar 13;350:h1269.
34. Conway R, Low C, Coughlan RJ, O'Donnell MJ, Carey JJ. Risk of liver injury among methotrexate users: A meta-analysis of randomised controlled trials. Semin Arthritis Rheum. 2015 Oct;45(2):156-62.
35. Costa R, Carneiro BA, Agulnik M, Rademaker AW, Pai SG, Villaflor VM, Cristofanilli M, Sosman JA, Giles FJ. Toxicity profile of approved anti-PD-1 monoclonal antibodies in solid tumors: a systematic review and meta-analysis of randomized clinical trials. Oncotarget. 2017 Jan 31;8(5):8910-8920.
36. Cui R, Chu L, Liu ZQ, Xiao YY, Zhu XL, Chen YJ, Xu Q. Hematologic toxicity assessment in solid tumor patients treated with cetuximab: A pooled analysis of 18 randomized controlled trials. Int J Cancer. 2016 Jun 1;138(11):2771-3.
37. Dahal A, Bellows BK, Sonpavde G, Tantravahi SK, Choueiri TK, Galsky MD, Agarwal N. Incidence of Severe Nephrotoxicity With Cisplatin Based on Renal Function Eligibility Criteria: Indirect Comparison Meta-analysis. Am J Clin Oncol. 2016 Oct;39(5):497-506.
38. Dahal K, Sharma S, Yousuf A, Lee J, Azrin M, Jimenez E, Modi K, Tandon N. A comparison of standard versus low dose heparin on access-related complications after coronary angiography through radial access: A meta-analysis of randomized controlled trials. Cardiovasc Revasc Med. 2018 Jul-Aug;19(5 Pt B):575-579.
39. Dicembrini I, Tomberli B, Nreu B, Baldereschi GI, Fanelli F, Mannucci E, Monami M. Peripheral artery disease and amputations with Sodium-Glucose co-Transporter-2 (SGLT-2) inhibitors: A meta-analysis of randomized controlled trials. Diabetes Res Clin Pract. 2019 Jul;153:138-144.
40. Donnan JR, Grandy CA, Chibrikov E, Marra CA, Aubrey-Bassler K, Johnston K, Swab M, Hache J, Curnew D, Nguyen H, Gamble JM. Comparative safety of the sodium glucose co-transporter 2 (SGLT2) inhibitors: a systematic review and meta-analysis. BMJ Open. 2019 Feb 1;9(1):e022577.
41. Dore MP, Bibbò S, Fresi G, Bassotti G, Pes GM. Side Effects Associated with Probiotic Use in Adult Patients with Inflammatory Bowel Disease: A Systematic Review and Meta-Analysis of Randomized Controlled Trials. Nutrients. 2019 Dec 2;11(12):2913.
42. Elgendy IY, Mahmoud AN, Barakat AF, Elgendy AY, Saad M, Abuzaid A, Wayangankar SA, Bavry AA. Cardiovascular Safety of Dipeptidyl-Peptidase IV Inhibitors: A Meta-Analysis of Placebo-Controlled Randomized Trials. Am J Cardiovasc Drugs. 2017 Apr;17(2):143-155.
43. Farah D, Leme GM, Eliaschewitz FG, Fonseca MCM. A safety and tolerability profile comparison between dipeptidyl peptidase-4 inhibitors and sulfonylureas in diabetic patients: A systematic review and meta-analysis. Diabetes Res Clin Pract. 2019 Mar;149:47-63.
44. Feng X, Tian M, Zhang W, Mei H. Gastrointestinal safety of etoricoxib in osteoarthritis and rheumatoid arthritis: A meta-analysis. PLoS One. 2018 Jan 10;13(1):e0190798.
45. Funakoshi T, Suzuki M, Muss HB. Infection risk in breast cancer patients treated with trastuzumab: a systematic review and meta-analysis. Breast Cancer Res Treat. 2015 Jan;149(2):321-30.
46. Gafter-Gvili A, Gurion R, Raanani P, Shpilberg O, Vidal L. Bendamustine-associated infections-systematic review and meta-analysis of randomized controlled trials. Hematol Oncol. 2017 Dec;35(4):424-431.
47. Geng Z, Yu Y, Hu S, Dong L, Ye C. Tocilizumab and the risk of respiratory adverse events in patients with rheumatoid arthritis: a systematic review and meta-analysis of randomised controlled trials. Clin Exp Rheumatol. 2019 Mar-Apr;37(2):318-323.
48. Ghatalia P, Je Y, Nguyen PL, Trinh QD, Choueiri TK, Sonpavde G. Fatigue with vascular endothelial growth factor receptor tyrosine kinase inhibitors and mammalian target of rapamycin inhibitors in patients with renal cell carcinoma (RCC) and other malignancies: A meta-analysis of randomized clinical trials. Crit Rev Oncol Hematol. 2015 Aug;95(2):251-63.
49. Gillies M, Ranakusuma A, Hoffmann T, Thorning S, McGuire T, Glasziou P, Del Mar C. Common harms from amoxicillin: a systematic review and meta-analysis of randomized placebo-controlled trials for any indication. CMAJ. 2015 Jan 6;187(1):E21-E31.
50. Gu B, Gao W, Chu H, Gao J, Fu Z, Ding H, Lv J, Wu Q. Adverse events risk associated with anti-VEGFR agents in the treatment of advanced nonsmall-cell lung cancer: A meta-analysis. Medicine (Baltimore). 2016 Nov;95(48):e3752.
51. Guo X, Yang Q, Dong J, Liao L, Zhang W, Liu F. Tumour Risk with Once-Weekly Glucagon-Like Peptide-1 Receptor Agonists in Type 2 Diabetes Mellitus Patients: A Systematic Review. Clin Drug Investig. 2016 Jun;36(6):433-41.
52. Gyawali B, Shimokata T, Ando M, Honda K, Ando Y. Risk of serious adverse events and fatal adverse events with sorafenib in patients with solid cancer: a meta-analysis of phase 3 randomized controlled trials†. Ann Oncol. 2017 Feb 1;28(2):246-253.
53. Hansen MP, Scott AM, McCullough A, Thorning S, Aronson JK, Beller EM, Glasziou PP, Hoffmann TC, Clark J, Del Mar CB. Adverse events in people taking macrolide antibiotics versus placebo for any indication. Cochrane Database Syst Rev. 2019 Jan 18;1(1):CD011825.
54. Hao S, Tian W, Gao B, Jiang Y, Zhang X, Zhang S, Guo L, Zhao J, Zhang G, Hu C, Yan J, Luo D. Does dual HER-2 blockade treatment increase the risk of severe toxicities of special interests in breast cancer patients: A meta-analysis of randomized controlled trials. Oncotarget. 2017 Mar 21;8(12):19923-19933.
55. Hill AM, Mitchell N, Hughes S, Pozniak AL. Risks of cardiovascular or central nervous system adverse events and immune reconstitution inflammatory syndrome, for dolutegravir versus other antiretrovirals: meta-analysis of randomized trials. Curr Opin HIV AIDS. 2018 Mar;13(2):102-111.
56. Holmskov M, Storebø OJ, Moreira-Maia CR, Ramstad E, Magnusson FL, Krogh HB, Groth C, Gillies D, Zwi M, Skoog M, Gluud C, Simonsen E. Gastrointestinal adverse events during methylphenidate treatment of children and adolescents with attention deficit hyperactivity disorder: A systematic review with meta-analysis and Trial Sequential Analysis of randomised clinical trials. PLoS One. 2017 Jun 15;12(6):e0178187.
57. Hong D, Zhang G, Zhang X, Liang X. Pulmonary Toxicities of Gefitinib in Patients With Advanced Non-Small-Cell Lung Cancer: A Meta-Analysis of Randomized Controlled Trials. Medicine (Baltimore). 2016 Mar;95(9):e3008.
58. Honvo G, Leclercq V, Geerinck A, Thomas T, Veronese N, Charles A, Rabenda V, Beaudart C, Cooper C, Reginster JY, Bruyère O. Safety of Topical Non-steroidal Anti-Inflammatory Drugs in Osteoarthritis: Outcomes of a Systematic Review and Meta-Analysis. Drugs Aging. 2019 Apr;36(Suppl 1):45-64.
59. Honvo G, Reginster JY, Rabenda V, Geerinck A, Mkinsi O, Charles A, Rizzoli R, Cooper C, Avouac B, Bruyère O. Safety of Symptomatic Slow-Acting Drugs for Osteoarthritis: Outcomes of a Systematic Review and Meta-Analysis. Drugs Aging. 2019 Apr;36(Suppl 1):65-99.
60. Honvo G, Reginster JY, Rannou F, Rygaert X, Geerinck A, Rabenda V, McAlindon T, Charles A, Fuggle N, Cooper C, Curtis E, Arden N, Avouac B, Bruyère O. Safety of Intra-articular Hyaluronic Acid Injections in Osteoarthritis: Outcomes of a Systematic Review and Meta-Analysis. Drugs Aging. 2019 Apr;36(Suppl 1):101-127.
61. Hu Y, Xu W, Cao F. A meta-analysis of randomized controlled trials: combination of ketamine and propofol versus ketamine alone for procedural sedation and analgesia in children. Intern Emerg Med. 2019 Oct;14(7):1159-1165.
62. Hua Q, Zhu Y, Liu H. Severe and fatal adverse events risk associated with rituximab addition to B-cell non-Hodgkin's lymphoma (B-NHL) chemotherapy: a meta-analysis. J Chemother. 2015;27(6):365-70.
63. Huang F, Luo ZC. Adverse drug events associated with 5mg versus 10mg Tofacitinib (Janus kinase inhibitor) twice daily for the treatment of autoimmune diseases: A systematic review and meta-analysis of randomized controlled trials. Clin Rheumatol. 2019 Feb;38(2):523-534.
64. Huang ST, Tian BS, Xiao O, Yang YJ, Zhou SY. Safety of antivascular endothelial growth factor administration in the ocular anterior segment in pterygium and neovascular glaucoma treatment: Systematic review and meta-analysis. Medicine (Baltimore). 2018 Aug;97(34):e11960.
65. Jalili M, Bahreini M, Doosti-Irani A, Masoomi R, Arbab M, Mirfazaelian H. Ketamine-propofol combination (ketofol) vs propofol for procedural sedation and analgesia: systematic review and meta-analysis. Am J Emerg Med. 2016 Mar;34(3):558-69.
66. Janjua S, Schmidt S, Ferrer M, Cates CJ. Inhaled steroids with and without regular formoterol for asthma: serious adverse events. Cochrane Database Syst Rev. 2019 Sep 25;9(9):CD006924.
67. Jia Z, Lu H, Yang X, Jin X, Wu R, Zhao J, Chen L, Qi Z. Adverse Events of Botulinum Toxin Type A in Facial Rejuvenation: A Systematic Review and Meta-Analysis. Aesthetic Plast Surg. 2016 Oct;40(5):769-77.
68. Johansen KGV, Tarp S, Astrup A, Lund H, Pagsberg AK, Christensen R. Harms associated with taking nalmefene for substance use and impulse control disorders: A systematic review and meta-analysis of randomised controlled trials. PLoS One. 2017 Aug 29;12(8):e0183821.
69. Khan AR, Bavishi C, Riaz H, Farid TA, Khan S, Atlas M, Hirsch G, Ikram S, Bolli R. Increased Risk of Adverse Neurocognitive Outcomes With Proprotein Convertase Subtilisin-Kexin Type 9 Inhibitors. Circ Cardiovasc Qual Outcomes. 2017 Jan;10(1):e003153.
70. Khosrow-Khavar F, Filion KB, Al-Qurashi S, Torabi N, Bouganim N, Suissa S, Azoulay L. Cardiotoxicity of aromatase inhibitors and tamoxifen in postmenopausal women with breast cancer: a systematic review and meta-analysis of randomized controlled trials. Ann Oncol. 2017 Mar 1;28(3):487-496.
71. Kim DH, Rogers JR, Fulchino LA, Kim CA, Solomon DH, Kim SC. Bisphosphonates and risk of cardiovascular events: a meta-analysis. PLoS One. 2015 Apr 17;10(4):e0122646.
72. Lao KS, He Y, Wong IC, Besag FM, Chan EW. Tolerability and Safety Profile of Cariprazine in Treating Psychotic Disorders, Bipolar Disorder and Major Depressive Disorder: A Systematic Review with Meta-Analysis of Randomized Controlled Trials. CNS Drugs. 2016 Nov;30(11):1043-1054.
73. Lew S, Chamberlain RS. Risk of Metabolic Complications in Patients with Solid Tumors Treated with mTOR inhibitors: Meta-analysis. Anticancer Res. 2016 Apr;36(4):1711-8.
74. Li H, Wang C, Zhang S, Sun S, Li R, Zou M, Cheng G. Safety Profile of Atorvastatin 80 mg: A Meta-Analysis of 17 Randomized Controlled Trials in 21,910 Participants. Drug Saf. 2016 May;39(5):409-19.
75. Li J, Gu J. Cardiovascular Toxicities with Vascular Endothelial Growth Factor Receptor Tyrosine Kinase Inhibitors in Cancer Patients: A Meta-Analysis of 77 Randomized Controlled Trials. Clin Drug Investig. 2018 Dec;38(12):1109-1123.
76. Li J, Gu J. Rash and Pruritus With PD-1 Inhibitors in Cancer Patients: A Meta-Analysis of Randomized Controlled Trials. J Clin Pharmacol. 2019 Jan;59(1):45-54.
77. Li J, Yan H. Skin toxicity with anti-EGFR monoclonal antibody in cancer patients: a meta-analysis of 65 randomized controlled trials. Cancer Chemother Pharmacol. 2018 Oct;82(4):571-583.
78. Li X, Wan J, Wu Z, Tu J, Hu Y, Wu S, Lou L. Fatal adverse events with molecular targeted agents in the treatment of advanced hepatocellular carcinoma: a meta-analysis of randomized controlled trials. Drug Des Devel Ther. 2018 Sep 18;12:3043-3049.
79. Liang XJ, Shen J. Adverse events risk associated with angiogenesis inhibitors addition to therapy in ovarian cancer: a meta-analysis of randomized controlled trials. Eur Rev Med Pharmacol Sci. 2016 Jun;20(12):2701-9.
80. Liu B, Ding F, Liu Y, et al. Incidence and risk of hypertension associated with vascular endothelial growth factor receptor tyrosine kinase inhibitors in cancer patients: a comprehensive network meta-analysis of 72 randomized controlled trials involving 30013 patients. Oncotarget. 2016;7(41):67661-67673. doi:10.18632/oncotarget.11813
81. Liu W, Ma X, Zhou W. Adverse events of benralizumab in moderate to severe eosinophilic asthma: A meta-analysis. Medicine (Baltimore). 2019 May;98(22):e15868.
82. Liu Y, Qi M, Hou S, Shao L, Zhang J, Li Y, Liu Q. Risk of rash associated with vandetanib treatment in non-small-cell lung cancer patients: A meta-analysis of 9 randomized controlled trials. Medicine (Baltimore). 2017 Oct;96(43):e8345.
83. Liu Y, Zhang X, Chai S, Zhao X, Ji L. Risk of Malignant Neoplasia with Glucagon-Like Peptide-1 Receptor Agonist Treatment in Patients with Type 2 Diabetes: A Meta-Analysis. J Diabetes Res. 2019 Jul 16;2019:1534365.
84. Luo L, Yuan X, Huang W, Ren F, Zhu H, Zheng Y, Tang L. Safety of coadministration of ezetimibe and statins in patients with hypercholesterolaemia: a meta-analysis. Intern Med J. 2015 May;45(5):546-57.
85. Luo W, Wang Z, Tian P, Li W. Safety and tolerability of PD-1/PD-L1 inhibitors in the treatment of non-small cell lung cancer: a meta-analysis of randomized controlled trials. J Cancer Res Clin Oncol. 2018 Oct;144(10):1851-1859.
86. Lv WW, Zhang JJ, Zhou XL, Song Z, Wei CM. Safety of combining vascular endothelial growth factor receptor tyrosine-kinase inhibitors with chemotherapy in patients with advanced non-small-cell lung cancer: A PRISMA-compliant meta-analysis. Medicine (Baltimore). 2019 Jun;98(23):e15806.
87. Ma C, Panaccione NR, Nguyen TM, Guizzetti L, Parker CE, Hussein IM, Vande Casteele N, Khanna R, Dulai PS, Singh S, Feagan BG, Jairath V. Adverse Events and Nocebo Effects in Inflammatory Bowel Disease: A Systematic Review and Meta-Analysis of Randomized Controlled Trials. J Crohns Colitis. 2019 Sep 19;13(9):1201-1216.
88. Ma H, Liu Y, Huang L, Zeng XT, Jin SH, Yue GJ, Tian X, Zhou JG. The Adverse Events of Oxycodone in Cancer-Related Pain: A Systematic Review and Meta-Analysis of Randomized Controlled Trials. Medicine (Baltimore). 2016 Apr;95(15):e3341.
89. Man J, Ritchie G, Links M, Lord S, Lee CK. Treatment-related toxicities of immune checkpoint inhibitors in advanced cancers: A meta-analysis. Asia Pac J Clin Oncol. 2018 Jun;14(3):141-152.
90. Martel S, Bruzzone M, Ceppi M, Maurer C, Ponde NF, Ferreira AR, Viglietti G, Del Mastro L, Prady C, de Azambuja E, Lambertini M. Risk of adverse events with the addition of targeted agents to endocrine therapy in patients with hormone receptor-positive metastatic breast cancer: A systematic review and meta-analysis. Cancer Treat Rev. 2018 Jan;62:123-132.
91. Mincu RI, Mahabadi AA, Michel L, Mrotzek SM, Schadendorf D, Rassaf T, Totzeck M. Cardiovascular Adverse Events Associated With BRAF and MEK Inhibitors: A Systematic Review and Meta-analysis. JAMA Netw Open. 2019 Aug 2;2(8):e198890.
92. Minozzi S, Bonovas S, Lytras T, Pecoraro V, González-Lorenzo M, Bastiampillai AJ, Gabrielli EM, Lonati AC, Moja L, Cinquini M, Marino V, Matucci A, Milano GM, Tocci G, Scarpa R, Goletti D, Cantini F. Risk of infections using anti-TNF agents in rheumatoid arthritis, psoriatic arthritis, and ankylosing spondylitis: a systematic review and meta-analysis. Expert Opin Drug Saf. 2016 Dec;15(sup1):11-34.
93. Miroddi M, Sterrantino C, Simmonds M, Caridi L, Calapai G, Phillips RS, Stewart LA. Systematic review and meta-analysis of the risk of severe and life-threatening thromboembolism in cancer patients receiving anti-EGFR monoclonal antibodies (cetuximab or panitumumab). Int J Cancer. 2016 Nov 15;139(10):2370-80.
94. Miroddi M, Sterrantino C, Simonelli I, Ciminata G, Phillips RS, Calapai G. Risk of grade 3-4 diarrhea and mucositis in colorectal cancer patients receiving anti-EGFR monoclonal antibodies regimens: A meta-analysis of 18 randomized controlled clinical trials. Crit Rev Oncol Hematol. 2015 Nov;96(2):355-71.
95. Misawa F, Kishimoto T, Hagi K, Kane JM, Correll CU. Safety and tolerability of long-acting injectable versus oral antipsychotics: A meta-analysis of randomized controlled studies comparing the same antipsychotics. Schizophr Res. 2016 Oct;176(2-3):220-230.
96. Penninga EI, Graudal N, Ladekarl MB, Jürgens G. Adverse Events Associated with Flumazenil Treatment for the Management of Suspected Benzodiazepine Intoxication--A Systematic Review with Meta-Analyses of Randomised Trials. Basic Clin Pharmacol Toxicol. 2016 Jan;118(1):37-44.
97. Pozzi M, Carnovale C, Peeters GGAM, Gentili M, Antoniazzi S, Radice S, Clementi E, Nobile M. Adverse drug events related to mood and emotion in paediatric patients treated for ADHD: A meta-analysis. J Affect Disord. 2018 Oct 1;238:161-178.
98. Puckrin R, Saltiel MP, Reynier P, Azoulay L, Yu OHY, Filion KB. SGLT-2 inhibitors and the risk of infections: a systematic review and meta-analysis of randomized controlled trials. Acta Diabetol. 2018 May;55(5):503-514.
99. Qi WX, Fu S, Zhang Q, Guo XM. Bevacizumab increases the risk of infections in cancer patients: A systematic review and pooled analysis of 41 randomized controlled trials. Crit Rev Oncol Hematol. 2015 Jun;94(3):323-36.
100. Raccah BH, Perlman A, Danenberg HD, Pollak A, Muszkat M, Matok I. Major Bleeding and Hemorrhagic Stroke With Direct Oral Anticoagulants in Patients With Renal Failure: Systematic Review and Meta-Analysis of Randomized Trials. Chest. 2016;149(6):1516-1524.
101. Rogers SC, Garcia CA, Wu S. Discontinuation of Everolimus Due to Related and Unrelated Adverse Events in Cancer Patients: A Meta-Analysis. Cancer Invest. 2017 Sep 14;35(8):552-561.
102. Roviello G, Generali D. Is the fatigue an adverse event of the second generation of hormonal therapy? Data from a literature-based meta-analysis. Med Oncol. 2018 Jan 31;35(3):29.
103. Rungapiromnan W, Yiu ZZN, Warren RB, Griffiths CEM, Ashcroft DM. Impact of biologic therapies on risk of major adverse cardiovascular events in patients with psoriasis: systematic review and meta-analysis of randomized controlled trials. Br J Dermatol. 2017 Apr;176(4):890-901.
104. Santoni M, Guerra F, Conti A, Lucarelli A, Rinaldi S, Belvederesi L, Capucci A, Berardi R. Incidence and risk of cardiotoxicity in cancer patients treated with targeted therapies. Cancer Treat Rev. 2017 Sep;59:123-131.
105. Sardar P, Udell JA, Chatterjee S, Bansilal S, Mukherjee D, Farkouh ME. Effect of Intensive Versus Standard Blood Glucose Control in Patients With Type 2 Diabetes Mellitus in Different Regions of the World: Systematic Review and Meta-analysis of Randomized Controlled Trials. J Am Heart Assoc. 2015 May 5;4(5):e001577.
106. Shah ED, Farida JP, Siegel CA, Chong K, Melmed GY. Risk for Overall Infection with Anti-TNF and Anti-integrin Agents Used in IBD: A Systematic Review and Meta-analysis. Inflamm Bowel Dis. 2017 Apr;23(4):570-577.
107. Sterling LH, Windle SB, Filion KB, Touma L, Eisenberg MJ. Varenicline and Adverse Cardiovascular Events: A Systematic Review and Meta-Analysis of Randomized Controlled Trials. J Am Heart Assoc. 2016 Feb 22;5(2):e002849.
108. Su Q, Zhang XC, Wang DY, Zhang HR, Zhu C, Hou YL, Liu JL, Gao ZH. The risk of immune-related endocrine disorders associated with anti-PD-1 inhibitors therapy for solid tumors: A systematic review and meta-analysis. Int Immunopharmacol. 2018 Jun;59:328-338.
109. Sun S, Cui Z, Zhou M, Li R, Li H, Zhang S, Ba Y, Cheng G. Proton pump inhibitor monotherapy and the risk of cardiovascular events in patients with gastro-esophageal reflux disease: a meta-analysis. Neurogastroenterol Motil. 2017 Feb;29(2).
110. Sun W, Li J. Skin Toxicities with Epidermal Growth Factor Receptor Tyrosine Kinase Inhibitors in Cancer Patients: A Meta-Analysis of Randomized Controlled Trials. Cancer Invest. 2019;37(6):253-264.
111. Tandan M, Cormican M, Vellinga A. Adverse events of fluoroquinolones vs. other antimicrobials prescribed in primary care: A systematic review and meta-analysis of randomized controlled trials. Int J Antimicrob Agents. 2018 Nov;52(5):529-540.
112. Tang B, Wang J, Luo LL, Li QG, Huang D. Risks of budesonide/formoterol for the treatment of stable COPD: a meta-analysis. Int J Chron Obstruct Pulmon Dis. 2019 Apr 1;14:757-766.
113. Thomas KH, Martin RM, Knipe DW, Higgins JP, Gunnell D. Risk of neuropsychiatric adverse events associated with varenicline: systematic review and meta-analysis. BMJ. 2015 Mar 12;350:h1109.
114. Tong H, Zhu Y, Liu Y. Incidence and risk of fatigue in cancer patients treated with MET inhibitors: A systematic review and meta-analysis. Medicine (Baltimore). 2019 May;98(22):e15522.
115. Tong S, Fan K, Jiang K, Zhai W, Fang B, Wang SH, Wang JJ. Increased risk of severe infections in non-small-cell lung cancer patients treated with pemetrexed: a meta-analysis of randomized controlled trials. Curr Med Res Opin. 2017 Jan;33(1):31-37.
116. Totzeck M, Mincu RI, Rassaf T. Cardiovascular Adverse Events in Patients With Cancer Treated With Bevacizumab: A Meta-Analysis of More Than 20 000 Patients. J Am Heart Assoc. 2017 Aug 10;6(8):e006278.
117. Varma A, Zis P. Nocebo effect in myasthenia gravis: systematic review and meta-analysis of placebo-controlled clinical trials. Acta Neurol Belg. 2019 Jun;119(2):257-264.
118. Varvaki Rados D, Catani Pinto L, Reck Remonti L, Bauermann Leitão C, Gross JL. The Association between Sulfonylurea Use and All-Cause and Cardiovascular Mortality: A Meta-Analysis with Trial Sequential Analysis of Randomized Clinical Trials. PLoS Med. 2016 Apr 12;13(4):e1001992.
119. Vinnakota DN, Kamatham R. Safety profile of phentolamine mesylate as reversal agent of pulpal and soft tissue dental anesthesia: a systematic review and meta-analysis. Quintessence Int. 2019;50(7):568-575.
120. Vukadinović D, Scholz SS, Messerli FH, Weber MA, Williams B, Böhm M, Mahfoud F. Peripheral edema and headache associated with amlodipine treatment: a meta-analysis of randomized, placebo-controlled trials. J Hypertens. 2019 Oct;37(10):2093-2103.
121. Wang J, Wang Z, Zhao Y. Incidence and risk of hypertension with ramucirumab in cancer patients: a meta-analysis of published studies. Clin Drug Investig. 2015 Apr;35(4):221-8.
122. Wang S, He Q, Shuai Z. Risk of serious infections in biological treatment of patients with ankylosing spondylitis and non-radiographic axial spondyloarthritis: a meta-analysis. Clin Rheumatol. 2018 Feb;37(2):439-450.
123. Wang T, Wang F, Zhou J, Tang H, Giovenale S. Adverse effects of incretin-based therapies on major cardiovascular and arrhythmia events: meta-analysis of randomized trials. Diabetes Metab Res Rev. 2016 Nov;32(8):843-857.
124. Wang W, Lie P, Guo M, He J. Risk of hepatotoxicity in cancer patients treated with immune checkpoint inhibitors: A systematic review and meta-analysis of published data. Int J Cancer. 2017 Sep 1;141(5):1018-1028.
125. Wei A, Gu Z, Li J, Liu X, Wu X, Han Y, Pu J. Clinical Adverse Effects of Endothelin Receptor Antagonists: Insights From the Meta-Analysis of 4894 Patients From 24 Randomized Double-Blind Placebo-Controlled Clinical Trials. J Am Heart Assoc. 2016 Oct 26;5(11):e003896.
126. Wei W, Luo Z. Risk of gastrointestinal toxicities with PD-1 inhibitors in cancer patients: A meta-analysis of randomized clinical trials. Medicine (Baltimore). 2017 Dec;96(48):e8931.
127. Wessler JD, Giugliano RP. Risk of thrombocytopenia with glycoprotein IIb/IIIa inhibitors across drugs and patient populations: a meta-analysis of 29 large placebo-controlled randomized trials. Eur Heart J Cardiovasc Pharmacother. 2015 Apr;1(2):97-106.
128. Xia N, Wang H, Nie X. Inhaled Long-Acting β2-Agonists Do Not Increase Fatal Cardiovascular Adverse Events in COPD: A Meta-Analysis. PLoS One. 2015 Sep 17;10(9):e0137904.
129. Xie W, Huang Y, Xiao S, Sun X, Fan Y, Zhang Z. Impact of Janus kinase inhibitors on risk of cardiovascular events in patients with rheumatoid arthritis: systematic review and meta-analysis of randomised controlled trials. Ann Rheum Dis. 2019 Aug;78(8):1048-1054.
130. Xing Y, Chen L, Feng Y, Zhou Y, Zhai Y, Lu J. Meta-analysis of the safety of voriconazole in definitive, empirical, and prophylactic therapies for invasive fungal infections. BMC Infect Dis. 2017 Dec 28;17(1):798.
131. Xu H, Tan P, Zheng X, Huang Y, Lin T, Wei Q, Ai J, Yang L. Immune-related adverse events following administration of anti-cytotoxic T-lymphocyte-associated protein-4 drugs: a comprehensive systematic review and meta-analysis. Drug Des Devel Ther. 2019 Jul 4;13:2215-2234.
132. Xu M, Nie Y, Yang Y, Lu YT, Su Q. Risk of Neurological Toxicities Following the Use of Different Immune Checkpoint Inhibitor Regimens in Solid Tumors: A Systematic Review and Meta-analysis. Neurologist. 2019 May;24(3):75-83.
133. Xue XJ, He RL, Li WX, Xin JW, Ye QY, Chen XC, Pan XD. [Safety of atypical antipsychotics in the treatment of behavioral and psychological symptoms in dementia: a meta-analysis]. Zhonghua Yi Xue Za Zhi. 2018 Jul 3;98(25):2030-2036. Chinese.
134. Yamada A, Wang J, Komaki Y, Komaki F, Micic D, Sakuraba A. Systematic review with meta-analysis: risk of new onset IBD with the use of anti-interleukin-17 agents. Aliment Pharmacol Ther. 2019 Aug;50(4):373-385.
135. Yang W, Li S, Yang Q. Risk of dermatologic and mucosal adverse events associated with PD-1/PD-L1 inhibitors in cancer patients: A meta-analysis of randomized controlled trials. Medicine (Baltimore). 2019 May;98(20):e15731.
136. Yang Y, Liu YH, Sun X, Yu MW, Yang L, Cheng PY, Yang GW, Wang XM. Risk of peripheral edema in cancer patients treated with MEK inhibitors: a systematic review and meta-analysis of clinical trials. Curr Med Res Opin. 2017 Sep;33(9):1663-1675.
137. Zhang B, Wu Q, Zhou YL, Guo X, Ge J, Fu J. Immune-related adverse events from combination immunotherapy in cancer patients: A comprehensive meta-analysis of randomized controlled trials. Int Immunopharmacol. 2018 Oct;63:292-298.
138. Zhang H, Huang Z, Zou X, Liu T. Bevacizumab and wound-healing complications: a systematic review and meta-analysis of randomized controlled trials. Oncotarget. 2016 Dec 13;7(50):82473-82481.
139. Zhang S, Liang F, Li W, Wang Q. Risk of treatment-related mortality in cancer patients treated with ipilimumab: A systematic review and meta-analysis. Eur J Cancer. 2017 Sep;83:71-79.
140. Zhang S, Liang F, Zhu J, Chen Q. Risk of Pneumonitis Associated with Programmed Cell Death 1 Inhibitors in Cancer Patients: A Meta-analysis. Mol Cancer Ther. 2017 Aug;16(8):1588-1595.
141. Zhang X, Ran Y, Shao Y, Wang K, Zhu Y. Incidence and risk of severe infections associated with aflibercept in cancer patients: a systematic review and meta-analysis. Br J Clin Pharmacol. 2016 Jan;81(1):33-40.
142. Zhang X, Ran Y, Wang K, Zhu Y, Li J. Incidence and risk of hepatic toxicities with PD-1 inhibitors in cancer patients: a meta-analysis. Drug Des Devel Ther. 2016 Sep 28;10:3153-3161.
143. Zhao B, Zhao H, Zhao J. Risk of fatal adverse events in cancer patients treated with sunitinib. Crit Rev Oncol Hematol. 2019 May;137:115-122.
144. Zhao YT, Li PY, Zhang JQ, Wang L, Yi Z. Angiotensin II Receptor Blockers and Cancer Risk: A Meta-Analysis of Randomized Controlled Trials. Medicine (Baltimore). 2016 May;95(18):e3600.
145. Zhou JX, Feng LJ, Zhang X. Risk of severe hematologic toxicities in cancer patients treated with PARP inhibitors: a meta-analysis of randomized controlled trials. Drug Des Devel Ther. 2017 Oct 13;11:3009-3017.
146. Zhou Y, Lu H, Yang M, Xu C. Adverse drug events associated with ibrutinib for the treatment of elderly patients with chronic lymphocytic leukemia: A systematic review and meta-analysis of randomized trials. Medicine (Baltimore). 2019 Aug;98(33):e16915.
147. Zhu J, Wu J, Li G, Li J, Lin Y, He Z, Su C, Zhao W, Wu Q, Chen Z, Qiu K. Meta-analysis of randomized controlled trials for the incidence and risk of fatal adverse events in cancer patients treated with ipilimumab. Expert Opin Drug Saf. 2017 Apr;16(4):423-428.
148. Zhu J, Zhao W, Liang D, Li G, Qiu K, Wu J, Li J. Risk of fatigue in cancer patients receiving anti-EGFR monoclonal antibodies: results from a systematic review and meta-analysis of randomized controlled trial. Int J Clin Oncol. 2018 Apr;23(2):389-399.
149. Zhu LN, Chen D, Chen T, Xu D, Chen SH, Liu L. The adverse event profile of brivaracetam: A meta-analysis of randomized controlled trials. Seizure. 2017 Feb; 45:7-16.
150. Zhu X, Tian X, Yu C, Hong J, Fang J, Chen H. Increased risk of hemorrhage in metastatic colorectal cancer patients treated with bevacizumab: An updated meta-analysis of 12 randomized controlled trials. Medicine (Baltimore). 2016 Aug;95(34): e4232.
151. Zhu X, Wu S. Increased Risk of Hypertension with Enzalutamide in Prostate Cancer: A Meta-Analysis. Cancer Invest. 2019;37(9):478-488.

**Appendix Table S4. List of excluded reviews (with reasons).**

| **Exclusion lists** | **Reasons for exclusion** |
| --- | --- |
| 1. Aires FT, Dedivitis RA, Petrarolha SM, Bernardo WM, Cernea CR, Brandão LG. Early oral feeding after total laryngectomy: A systematic review. Head Neck. 2015;37(10):1532-1535. doi:10.1002/hed.23755 | NRSIs included, less than 5 studies |
| 1. Alfageh BH, Wang Z, Mongkhon P, et al. Safety and Tolerability of Antipsychotic Medication in Individuals with Autism Spectrum Disorder: A Systematic Review and Meta-Analysis. Paediatr Drugs. 2019;21(3):153-167. doi:10.1007/s40272-019-00333-x | NRSIs included |
| 1. Aljebab F, Choonara I, Conroy S. Systematic review of the toxicity of short-course oral corticosteroids in children. Arch Dis Child. 2016;101(4):365-370. doi:10.1136/archdischild-2015-309522 | NRSIs included |
| 1. Aljebab F, Choonara I, Conroy S. Systematic Review of the Toxicity of Long-Course Oral Corticosteroids in Children. PLoS One. 2017;12(1):e0170259. Published 2017 Jan 26. doi:10.1371/journal.pone.0170259 | NRSIs included |
| 1. Almpani K, Papageorgiou SN, Papadopoulos MA. Autotransplantation of teeth in humans: a systematic review and meta-analysis. Clin Oral Investig. 2015;19(6):1157-1179. doi:10.1007/s00784-015-1473-9 | NRSIs included |
| 1. Ando T, Briasoulis A, Holmes AA, Takagi H, Slovut DP. Percutaneous versus surgical cut-down access in transfemoral transcatheter aortic valve replacement: A meta-analysis. J Card Surg. 2016;31(12):710-717. doi:10.1111/jocs.12842 | NRSIs included |
| 1. Almufleh A, Ramirez FD, So D, Le May M, Chong AY, Torabi N, Hibbert B. H2 Receptor Antagonists versus Proton Pump Inhibitors in Patients on Dual Antiplatelet Therapy for Coronary Artery Disease: A Systematic Review. Cardiology. 2018;140(2):115-123. | NRSIs included |
| 1. Azoulay L, Suissa S. Sulfonylureas and the Risks of Cardiovascular Events and Death: A Methodological Meta-Regression Analysis of the Observational Studies. Diabetes Care. 2017;40(5):706-714. doi:10.2337/dc16-1943 | NRSIs included |
| 1. Bai Y, Chen H, Yang Y, et al. Safety of antithrombotic drugs in patients with atrial fibrillation and non-end-stage chronic kidney disease: Meta-analysis and systematic review. Thromb Res. 2016;137:46-52. doi:10.1016/j.thromres.2015.11.020 | NRSIs included |
| 1. Balasubramanian I, Fleming C, Mohan HM, Schmidt K, Haglind E, Winter DC. Out-Patient Management of Mild or Uncomplicated Diverticulitis: A Systematic Review. Dig Surg. 2017;34(2):151-160. doi:10.1159/000450865 | NRSIs included |
| 1. Balk EM, Earley A, Avendano EA, Raman G. Long-Term Health Outcomes in Women With Silicone Gel Breast Implants: A Systematic Review. Ann Intern Med. 2016;164(3):164-175. doi:10.7326/M15-1169 | NRSIs included |
| 1. Belum VR, Benhuri B, Postow MA, Hellmann MD, Lesokhin AM, Segal NH, Motzer RJ, Wu S, Busam KJ, Wolchok JD, Lacouture ME. Characterisation and management of dermatologic adverse events to agents targeting the PD-1 receptor. Eur J Cancer. 2016 Jun;60:12-25. | NRSIs included |
| 1. Beger HG, Siech M, Poch B, Mayer B, Schoenberg MH. Limited surgery for benign tumours of the pancreas: a systematic review. World J Surg. 2015;39(6):1557-1566. doi:10.1007/s00268-015-2976-x | NRSIs included |
| 1. Belum VR, Benhuri B, Postow MA, et al. Characterisation and management of dermatologic adverse events to agents targeting the PD-1 receptor. Eur J Cancer. 2016;60:12-25. doi:10.1016/j.ejca.2016.02.010 | NRSIs included |
| 1. Berstock JR, Blom AW, Beswick AD. A systematic review and meta-analysis of complications following the posterior and lateral surgical approaches to total hip arthroplasty. Ann R Coll Surg Engl. 2015;97(1):11-16. doi:10.1308/003588414X13946184904008 | NRSIs included |
| 1. Boleto G, Kanagaratnam L, Dramé M, Salmon JH. Safety of combination therapy with two bDMARDs in patients with rheumatoid arthritis: A systematic review and meta-analysis. Semin Arthritis Rheum. 2019;49(1):35-42. doi:10.1016/j.semarthrit.2018.12.003 | NRSIs included |
| 1. Caldeira D, Ferreira JJ, Pinto FJ, Costa J. Safety of non-vitamin K antagonist oral anticoagulants - coronary risks. Expert Opin Drug Saf. 2016;15(6):731-740. doi:10.1517/14740338.2016.1164689 | NRSIs included |
| 1. Castagné B, Viprey M, Martin J, Schott AM, Cucherat M, Soubrier M. Cardiovascular safety of tocilizumab: A systematic review and network meta-analysis. PLoS One. 2019;14(8):e0220178. Published 2019 Aug 1. doi:10.1371/journal.pone.0220178 | NRSIs included |
| 1. Ceresoli M, Tamini N, Gianotti L, Braga M, Nespoli L. Are endoscopic loop ties safe even in complicated acute appendicitis? A systematic review and meta-analysis. Int J Surg. 2019;68:40-47. doi:10.1016/j.ijsu.2019.06.011 | NRSIs included |
| 1. Cheng D, Gao H, Li W. Long-term risk of rosiglitazone on cardiovascular events - a systematic review and meta-analysis. Endokrynol Pol. 2018;69(4):381-394. doi:10.5603/EP.a2018.0036 | NRSIs included |
| 1. Chi CC, Wang SH, Wojnarowska F, Kirtschig G, Davies E, Bennett C. Safety of topical corticosteroids in pregnancy. Cochrane Database Syst Rev. 2015;(10):CD007346. Published 2015 Oct 26. doi:10.1002/14651858.CD007346.pub3 | NRSIs included |
| 1. Ching C, Eslick GD, Poulton AS. Evaluation of Methylphenidate Safety and Maximum-Dose Titration Rationale in Attention-Deficit/Hyperactivity Disorder: A Meta-analysis. JAMA Pediatr. 2019;173(7):630-639. doi:10.1001/jamapediatrics.2019.0905 | NRSIs included |
| 1. Craveiro NS, Silva Lopes B, Tomás L, et al. L-TRUST: Long-term risk of cancer in patients under statins therapy. A systematic review and meta-analysis. Pharmacoepidemiol Drug Saf. 2019;28(11):1431-1439. doi:10.1002/pds.4895 | NRSIs included |
| 1. Dai J, Belum VR, Wu S, Sibaud V, Lacouture ME. Pigmentary changes in patients treated with targeted anticancer agents: A systematic review and meta-analysis. J Am Acad Dermatol. 2017;77(5):902-910.e2. doi:10.1016/j.jaad.2017.06.044 | No 2 by 2 table data |
| 1. de Filette J, Andreescu CE, Cools F, Bravenboer B, Velkeniers B. A Systematic Review and Meta-Analysis of Endocrine-Related Adverse Events Associated with Immune Checkpoint Inhibitors. Horm Metab Res. 2019;51(3):145-156. doi:10.1055/a-0843-3366 | NRSIs included |
| 1. de La Forest Divonne M, Gottenberg JE, Salliot C. Safety of biologic DMARDs in RA patients in real life: A systematic literature review and meta-analyses of biologic registers. Joint Bone Spine. 2017;84(2):133-140. doi:10.1016/j.jbspin.2016.02.028 | NRSIs included |
| 1. de Resende JA Júnior, Cavalini LT, Crispi CP, de Freitas Fonseca M. Risk of urinary retention after nerve-sparing surgery for deep infiltrating endometriosis: A systematic review and meta-analysis. Neurourol Urodyn. 2017;36(1):57-61. doi:10.1002/nau.22915 | Less than 5 studies |
| 1. Delanoy N, Pécuchet N, Fabre E, et al. Bleomycin-Induced Pneumonitis in the Treatment of Ovarian Sex Cord-Stromal Tumors: A Systematic Review and Meta-analysis. Int J Gynecol Cancer. 2015;25(9):1593-1598. doi:10.1097/IGC.0000000000000530 | NRSIs included |
| 1. Desai RJ, Thaler KJ, Mahlknecht P, et al. Comparative Risk of Harm Associated With the Use of Targeted Immunomodulators: A Systematic Review. Arthritis Care Res (Hoboken). 2016;68(8):1078-1088. doi:10.1002/acr.22815 | NRSIs included |
| 1. Di Franco R, Borzillo V, Ravo V, et al. Rectal/urinary toxicity after hypofractionated vs. conventional radiotherapy in high risk prostate cancer: systematic review and meta analysis. Eur Rev Med Pharmacol Sci. 2017;21(16):3563-3575. | NRSIs included |
| 1. Di Franco R, Borzillo V, Ravo V, et al. Rectal/urinary toxicity after hypofractionated vs conventional radiotherapy in low/intermediate risk localized prostate cancer: systematic review and meta analysis. Oncotarget. 2017;8(10):17383-17395. doi:10.18632/oncotarget.14798 | NRSIs included, less than 5 studies |
| 1. Diener C, Horneff G. Comparison of adverse events of biologicals for treatment of juvenile idiopathic arthritis: a systematic review. Expert Opin Drug Saf. 2019;18(8):719-732. doi:10.1080/14740338.2019.1632288 | NRSIs included |
| 1. Ding J, Han J, Jing Z, Jiang Y. Is it dangerous to treat acute ischemic stroke by thrombolytic therapy in patients with comorbid intracranial aneurysms?. Am J Emerg Med. 2016;34(3):636-642. doi:10.1016/j.ajem.2015.12.025 | NRSIs included |
| 1. Doleman B, Moppett IK. Is early hip fracture surgery safe for patients on clopidogrel? Systematic review, meta-analysis and meta-regression. Injury. 2015;46(6):954-962. doi:10.1016/j.injury.2015.03.024 | NRSIs included |
| 1. Dong J, Meng X, Li S, et al. Risk of Adverse Vascular Events in Patients with Malignant Glioma Treated with Bevacizumab Plus Irinotecan: A Systematic Review and Meta-Analysis. World Neurosurg. 2019;130:e236-e243. doi:10.1016/j.wneu.2019.06.043 | NRSIs included |
| 1. Duarte GS, Nunes-Ferreira A, Rodrigues FB, et al. Morphine in acute coronary syndrome: systematic review and meta-analysis. BMJ Open. 2019;9(3):e025232. Published 2019 Mar 15. doi:10.1136/bmjopen-2018-025232 | NRSIs included |
| 1. Eke AC, Shukr GH, Chaalan TT, Nashif SK, Eleje GU. Intra-abdominal saline irrigation at cesarean section: a systematic review and meta-analysis. J Matern Fetal Neonatal Med. 2016;29(10):1588-1594. doi:10.3109/14767058.2015.1055723 | Less than 5 studies |
| 1. Esaiassen E, Fjalstad JW, Juvet LK, van den Anker JN, Klingenberg C. Antibiotic exposure in neonates and early adverse outcomes: a systematic review and meta-analysis. J Antimicrob Chemother. 2017;72(7):1858-1870. doi:10.1093/jac/dkx088 | NRSIs included |
| 1. Fan H, Li L, Wijlaars L, Gilbert RE. Associations between use of macrolide antibiotics during pregnancy and adverse child outcomes: A systematic review and meta-analysis. PLoS One. 2019;14(2):e0212212. Published 2019 Feb 19. doi:10.1371/journal.pone.0212212 | NRSIs included |
| 1. Geminiani A, Tsigarida A, Chochlidakis K, Papaspyridakos PV, Feng C, Ercoli C. A meta-analysis of complications during sinus augmentation procedure. Quintessence Int. 2017;48(3):231-240. doi:10.3290/j.qi.a37644 | NRSIs included |
| 1. Geng HZ, Nasier D, Liu B, Gao H, Xu YK. Meta-analysis of elective surgical complications related to defunctioning loop ileostomy compared with loop colostomy after low anterior resection for rectal carcinoma. Ann R Coll Surg Engl. 2015;97(7):494-501. doi:10.1308/003588415X14181254789240 | NRSIs included |
| 1. Ghayoumi P, Kandemir U, Morshed S. Evidence based update: open versus closed reduction. Injury. 2015;46(3):467-473. doi:10.1016/j.injury.2014.10.011 | NRSIs included |
| 1. Gorelik E, Masarwa R, Perlman A, et al. Fluoroquinolones and Cardiovascular Risk: A Systematic Review, Meta-analysis and Network Meta-analysis. Drug Saf. 2019;42(4):529-538. doi:10.1007/s40264-018-0751-2 | NRSIs included |
| 1. Gunter BR, Butler KA, Wallace RL, Smith SM, Harirforoosh S. Non-steroidal anti-inflammatory drug-induced cardiovascular adverse events: a meta-analysis. J Clin Pharm Ther. 2017;42(1):27-38. doi:10.1111/jcpt.12484 | NRSIs included |
| 1. Hougen I, Collister D, Bourrier M, et al. Safety of Intravenous Iron in Dialysis: A Systematic Review and Meta-Analysis. Clin J Am Soc Nephrol. 2018;13(3):457-467. doi:10.2215/CJN.05390517 | NRSIs included |
| 1. Hovaguimian F, Köppel S, Spahn DR. Safety of Anticoagulation Interruption in Patients Undergoing Surgery or Invasive Procedures: A Systematic Review and Meta-analyses of Randomized Controlled Trials and Non-randomized Studies. World J Surg. 2017;41(10):2444-2456. doi:10.1007/s00268-017-4072-x | NRSIs included, less than 5 studies |
| 1. Inokuchi M, Otsuki S, Fujimori Y, Sato Y, Nakagawa M, Kojima K. Systematic review of anastomotic complications of esophagojejunostomy after laparoscopic total gastrectomy. World J Gastroenterol. 2015;21(32):9656-9665. doi:10.3748/wjg.v21.i32.9656 | NRSIs included |
| 1. Inokuchi M, Sugita H, Otsuki S, Sato Y, Nakagawa M, Kojima K. Laparoscopic distal gastrectomy reduced surgical site infection as compared with open distal gastrectomy for gastric cancer in a meta-analysis of both randomized controlled and case-controlled studies. Int J Surg. 2015;15:61-67. doi:10.1016/j.ijsu.2015.01.030 | NRSIs included |
| 1. Jesson J, Dahourou DL, Renaud F, Penazzato M, Leroy V. Adverse events associated with abacavir use in HIV-infected children and adolescents: a systematic review and meta-analysis. Lancet HIV. 2016;3(2):e64-e75. doi:10.1016/S2352-3018(15)00225-8 | NRSIs included |
| 1. Jiang Z, Xiao H, Zhang H, Liu S, Meng J. Comparison of adverse events between cluster and conventional immunotherapy for allergic rhinitis patients with or without asthma: A systematic review and meta-analysis. Am J Otolaryngol. 2019;40(6):102269. doi:10.1016/j.amjoto.2019.07.013 | NRSIs included |
| 1. Johnstone MS. Vertical Rectus Abdominis Myocutaneous Versus Alternative Flaps for Perineal Repair After Abdominoperineal Excision of the Rectum in the Era of Laparoscopic Surgery. Ann Plast Surg. 2017;79(1):101-106. doi:10.1097/SAP.0000000000001137 | NRSIs included |
| 1. Kaneoka A, Pisegna JM, Saito H, et al. A systematic review and meta-analysis of pneumonia associated with thin liquid vs. thickened liquid intake in patients who aspirate. Clin Rehabil. 2017;31(8):1116-1125. doi:10.1177/0269215516677739 | NRSIs included |
| 1. Keir A, Pal S, Trivella M, et al. Adverse effects of red blood cell transfusions in neonates: a systematic review and meta-analysis. Transfusion. 2016;56(11):2773-2780. doi:10.1111/trf.13785 | NRSIs included |
| 1. Khoja L, Day D, Wei-Wu Chen T, Siu LL, Hansen AR. Tumour- and class-specific patterns of immune-related adverse events of immune checkpoint inhibitors: a systematic review. Ann Oncol. 2017;28(10):2377-2385. doi:10.1093/annonc/mdx286 | No 2 by 2 table data |
| 1. Kovacs SD, van Eijk AM, Sevene E, et al. The Safety of Artemisinin Derivatives for the Treatment of Malaria in the 2nd or 3rd Trimester of Pregnancy: A Systematic Review and Meta-Analysis. PLoS One. 2016;11(11):e0164963. Published 2016 Nov 8. doi:10.1371/journal.pone.0164963 | NRSIs included |
| 1. Leung HW, Chan AL. Trastuzumab-induced cardiotoxicity in elderly women with HER-2-positive breast cancer: a meta-analysis of real-world data. Expert Opin Drug Saf. 2015;14(11):1661-1671. doi:10.1517/14740338.2015.1089231 | NRSIs included |
| 1. Li-Kim-Moy J, Yin JK, Rashid H, et al. Systematic review of fever, febrile convulsions and serious adverse events following administration of inactivated trivalent influenza vaccines in children [published correction appears in Euro Surveill. 2015;20(25). pii: 21164]. Euro Surveill. 2015;20(24):21159. Published 2015 Jun 18. doi:10.2807/1560-7917.es2015.20.24.21159 | NRSIs included |
| 1. Lin YS, Cheng SW, Wang YH, Chen KH, Fang CJ, Chen C. Systematic review with meta-analysis: risk of post-operative complications associated with pre-operative exposure to anti-tumour necrosis factor agents for Crohn's disease. Aliment Pharmacol Ther. 2019;49(8):966-977. doi:10.1111/apt.15184 | NRSIs included |
| 1. Liu P, Lin H, Chen Y, Wu YS, Tang M, Liu C. Comparison of Metal and Plastic Stents for Preoperative Biliary Drainage in Resectable and Borderline Resectable Periampullary Cancer: A Meta-Analysis and System Review. J Laparoendosc Adv Surg Tech A. 2018;28(9):1074-1082. doi:10.1089/lap.2018.0029 | NRSIs included |
| 1. Liu YX, Zhang Y, Huang JF, Wang L. Meta-analysis comparing the safety of laparoscopic and open surgical approaches for suspected adnexal mass during the second trimester. Int J Gynaecol Obstet. 2017;136(3):272-279. doi:10.1002/ijgo.12069 | NRSIs included |
| 1. Lucchetta RC, Leonart LP, Becker J, Pontarolo R, Fernandez-Llimós F, Wiens A. Safety outcomes of disease-modifying therapies for relapsing-remitting multiple sclerosis: A network meta-analysis. Mult Scler Relat Disord. 2019;35:7-15. doi:10.1016/j.msard.2019.06.036 | NRSIs included |
| 1. Lussana F, Cattaneo M, Rambaldi A, Squizzato A. Ruxolitinib-associated infections: A systematic review and meta-analysis. Am J Hematol. 2018;93(3):339-347. doi:10.1002/ajh.24976 | NRSIs included |
| 1. Lv S, Wang J, Xu Y. Safety of insulin analogs during pregnancy: a meta-analysis. Arch Gynecol Obstet. 2015;292(4):749-756. doi:10.1007/s00404-015-3692-3 | NRSIs included |
| 1. Lv Z, Li Y, Wu Y, Qu Y. Surgical complications of primary rhegmatogenous retinal detachment: a meta-analysis. PLoS One. 2015;10(3):e0116493. Published 2015 Mar 3. doi:10.1371/journal.pone.0116493 | NRSIs included |
| 1. Maniotis C, Andreou C, Karalis I, Koutouzi G, Agelaki M, Koutouzis M. A systematic review on the safety of Prostar XL versus ProGlide after TAVR and EVAR. Cardiovasc Revasc Med. 2017;18(2):145-150. doi:10.1016/j.carrev.2016.11.004 | NRSIs included |
| 1. Mattishent K, Thavarajah M, Sinha A, et al. Safety of 80% vs 30-35% fraction of inspired oxygen in patients undergoing surgery: a systematic review and meta-analysis. Br J Anaesth. 2019;122(3):311-324. doi:10.1016/j.bja.2018.11.026 | NRSIs included |
| 1. Menne J, Dumann E, Haller H, Schmidt BMW. Acute kidney injury and adverse renal events in patients receiving SGLT2-inhibitors: A systematic review and meta-analysis. PLoS Med. 2019;16(12):e1002983. Published 2019 Dec 9. doi:10.1371/journal.pmed.1002983 | NRSIs included |
| 1. Mikačić I, Bosnar D. Intravitreal Bevacizumab and Cardiovascular Risk in Patients with Age-Related Macular Degeneration: Systematic Review and Meta-Analysis of Randomized Controlled Trials and Observational Studies. Drug Saf. 2016;39(6):517-541. doi:10.1007/s40264-016-0408-y | NRSIs included |
| 1. Morris BJ, Krieger JN. Does Circumcision Increase Meatal Stenosis Risk?-A Systematic Review and Meta-analysis. Urology. 2017;110:16-26. doi:10.1016/j.urology.2017.07.027 | NRSIs included |
| 1. Mozaffari S, Abdolghaffari AH, Nikfar S, Abdollahi M. Pregnancy outcomes in women with inflammatory bowel disease following exposure to thiopurines and antitumor necrosis factor drugs: a systematic review with meta-analysis. Hum Exp Toxicol. 2015;34(5):445-459. doi:10.1177/0960327114550882 | NRSIs included |
| 1. Nagy A, Mátrai P, Hegyi P, et al. The effects of TNF-alpha inhibitor therapy on the incidence of infection in JIA children: a meta-analysis. Pediatr Rheumatol Online J. 2019;17(1):4. Published 2019 Jan 18. doi:10.1186/s12969-019-0305-x | NRSIs included |
| 1. Nicolas P, Maia MF, Bassat Q, et al. Safety of oral ivermectin during pregnancy: a systematic review and meta-analysis. Lancet Glob Health. 2020;8(1):e92-e100. doi:10.1016/S2214-109X(19)30453-X | NRSIs included |
| 1. Ouyang AJ, Lv YN, Zhong HL, et al. Meta-analysis of digoxin use and risk of mortality in patients with atrial fibrillation. Am J Cardiol. 2015;115(7):901-906. doi:10.1016/j.amjcard.2015.01.013 | NRSIs included |
| 1. Pecorelli N, Greco M, Amodeo S, Braga M. Small bowel obstruction and incisional hernia after laparoscopic and open colorectal surgery: a meta-analysis of comparative trials. Surg Endosc. 2017;31(1):85-99. doi:10.1007/s00464-016-4995-6 | NRSIs included, no 2 by 2 table data |
| 1. Peng C, Ling Y, Ma C, et al. Safety Outcomes of NOTES Cholecystectomy Versus Laparoscopic Cholecystectomy: A Systematic Review and Meta-Analysis. Surg Laparosc Endosc Percutan Tech. 2016;26(5):347-353. doi:10.1097/SLE.0000000000000284 | NRSIs included |
| 1. Pillai RN, Behera M, Owonikoko TK, et al. Comparison of the toxicity profile of PD-1 versus PD-L1 inhibitors in non-small cell lung cancer: A systematic analysis of the literature. Cancer. 2018;124(2):271-277. doi:10.1002/cncr.31043 | NRSIs included |
| 1. Quagliato LA, Cosci F, Shader RI, Silberman EK, Starcevic V, Balon R, Dubovsky SL, Salzman C, Krystal JH, Weintraub SJ, Freire RC, Nardi AE; International Task Force on Benzodiazepines. Selective serotonin reuptake inhibitors and benzodiazepines in panic disorder: A meta-analysis of common side effects in acute treatment. J Psychopharmacol. 2019 Nov;33(11):1340-1351. | NRSIs included |
| 1. Roberto G, Raschi E, Piccinni C, et al. Adverse cardiovascular events associated with triptans and ergotamines for treatment of migraine: systematic review of observational studies. Cephalalgia. 2015;35(2):118-131. doi:10.1177/0333102414550416 | NRSIs included |
| 1. Salim SA, Cheungpasitporn W, Elmaraezy A, et al. Infectious complications and mortality associated with the use of IV iron therapy: a systematic review and meta-analysis. Int Urol Nephrol. 2019;51(10):1855-1865. doi:10.1007/s11255-019-02273-4 | NRSIs included, no 2 by 2 table data |
| 1. Sandberg F, Viktorsdóttir MB, Salö M, Stenström P, Arnbjörnsson E. Comparison of major complications in children after laparoscopy-assisted gastrostomy and percutaneous endoscopic gastrostomy placement: a meta-analysis. Pediatr Surg Int. 2018;34(12):1321-1327. doi:10.1007/s00383-018-4358-6 | NRSIs included |
| 1. Shah K, Chaker Z, Busu T, et al. Meta-Analysis Comparing Renal Outcomes after Transcatheter versus Surgical Aortic Valve Replacement. J Interv Cardiol. 2019;2019:3537256. Published 2019 Apr 24. doi:10.1155/2019/3537256 | NRSIs included |
| 1. Shahi V, Brinjikji W, Murad MH, Asirvatham SJ, Kallmes DF. Safety of Uninterrupted Warfarin Therapy in Patients Undergoing Cardiovascular Endovascular Procedures: A Systematic Review and Meta-Analysis. Radiology. 2016;278(2):383-394. doi:10.1148/radiol.2015142531 | NRSIs included, no 2 by 2 table data |
| 1. Shen Y, Jia Y, Zhou J, Ji J, Xun P. Bayesian Network Meta-Analysis for Assessing Adverse Effects of Anti-hepatitis B Drugs. Clin Drug Investig. 2019;39(9):835-846. doi:10.1007/s40261-019-00802-8 | NRSIs included |
| 1. Sonalkar S, Kapp N. Intrauterine device insertion in the postpartum period: a systematic review. Eur J Contracept Reprod Health Care. 2015;20(1):4-18. doi:10.3109/13625187.2014.971454 | NRSIs included |
| 1. Soo CG, Della Torre PK, Yolland TJ, Shatwell MA. Clopidogrel and hip fractures, is it safe? A systematic review and meta-analysis. BMC Musculoskelet Disord. 2016;17:136. Published 2016 Mar 22. doi:10.1186/s12891-016-0988-9 | NRSIs included |
| 1. Sui JD, Wang Y, Wan Y, Wu YZ. Risk of hematologic toxicities with programmed cell death-1 inhibitors in cancer patients: a meta-analysis of current studies. Drug Des Devel Ther. 2018;12:1645-1657. Published 2018 Jun 8. doi:10.2147/DDDT.S167077 | NRSIs included |
| 1. Switzer NJ, Dykstra MA, Gill RS, et al. Endoscopic versus open component separation: systematic review and meta-analysis. Surg Endosc. 2015;29(4):787-795. doi:10.1007/s00464-014-3741-1 | NRSIs included |
| 1. Tian R, Yan H, Zhang F, et al. Incidence and relative risk of hemorrhagic events associated with ramucirumab in cancer patients: a systematic review and meta-analysis. Oncotarget. 2016;7(40):66182-66191. doi:10.18632/oncotarget.11097 | NRSIs included |
| 1. Tricco AC, Soobiah C, Blondal E, et al. Comparative safety of serotonin (5-HT3) receptor antagonists in patients undergoing surgery: a systematic review and network meta-analysis. BMC Med. 2015;13:142. Published 2015 Jun 18. doi:10.1186/s12916-015-0379-3 | NRSIs included |
| 1. Vaos G, Dimopoulou A, Gkioka E, Zavras N. Immediate surgery or conservative treatment for complicated acute appendicitis in children? A meta-analysis. J Pediatr Surg. 2019;54(7):1365-1371. doi:10.1016/j.jpedsurg.2018.07.017 | NRSIs included |
| 1. Vavken J, Mameghani A, Vavken P, Schaeren S. Complications and cancer rates in spine fusion with recombinant human bone morphogenetic protein-2 (rhBMP-2). Eur Spine J. 2016;25(12):3979-3989. doi:10.1007/s00586-015-3870-9 | NRSIs included |
| 1. Veroniki AA, Rios P, Cogo E, et al. Comparative safety of antiepileptic drugs for neurological development in children exposed during pregnancy and breast feeding: a systematic review and network meta-analysis. BMJ Open. 2017;7(7):e017248. Published 2017 Jul 20. doi:10.1136/bmjopen-2017-017248 | NRSIs included |
| 1. Wang FB, Pu YW, Zhong FY, Lv XD, Yang ZX, Xing CG. Laparoscopic permanent sigmoid stoma creation through the extraperitoneal route versus transperitoneal route. A meta-analysis of stoma-related complications. Saudi Med J. 2015;36(2):159-163. doi:10.15537/smj.2015.2.10203 | NRSIs included |
| 1. Whitlock EP, Burda BU, Williams SB, Guirguis-Blake JM, Evans CV. Bleeding Risks With Aspirin Use for Primary Prevention in Adults: A Systematic Review for the U.S. Preventive Services Task Force. Ann Intern Med. 2016;164(12):826-835. doi:10.7326/M15-2112 | NRSIs included |
| 1. Winberg H, Arnbjörnsson E, Anderberg M, Stenström P. Postoperative outcomes in distal hypospadias: a meta-analysis of the Mathieu and tubularized incised plate repair methods for development of urethrocutaneous fistula and urethral stricture. Pediatr Surg Int. 2019;35(11):1301-1308. doi:10.1007/s00383-019-04523-z | NRSIs included |
| 1. Wu Z, Zhang H, Jin W, et al. The Effect of Renin-Angiotensin-Aldosterone System Blockade Medications on Contrast-Induced Nephropathy in Patients Undergoing Coronary Angiography: A Meta-Analysis. PLoS One. 2015;10(6):e0129747. Published 2015 Jun 17. doi:10.1371/journal.pone.0129747 | NRSIs included |
| 1. Xia Y, Zhao J, Cao DS. Safety of Lipoabdominoplasty Versus Abdominoplasty: A Systematic Review and Meta-analysis. Aesthetic Plast Surg. 2019;43(1):167-174. doi:10.1007/s00266-018-1270-3 | NRSIs included |
| 1. Xu N, Yu M, Liu X, Sun C, Chen Z, Liu Z. A systematic review of complications in thoracic spine surgery for ossification of the posterior longitudinal ligament. Eur Spine J. 2017;26(7):1803-1809. doi:10.1007/s00586-015-4097-5 | NRSIs included |
| 1. Yang C, Yi Q, Zhang L, Cui H, Mao J. Safety of aripiprazole for tics in children and adolescents: A systematic review and meta-analysis. Medicine (Baltimore). 2019;98(22):e15816. doi:10.1097/MD.0000000000015816 | NRSIs included |
| 1. Yoon BH, Ha YC, Lee YK, Koo KH. Postoperative Deep Infection After Cemented Versus Cementless Total Hip Arthroplasty: A Meta-Analysis. J Arthroplasty. 2015;30(10):1823-1827. doi:10.1016/j.arth.2015.04.041 | NRSIs included |
| 1. You CH, Lin CK, Chen PH, et al. Clarithromycin use and the risk of mortality and cardiovascular events: A systematic review and meta-analysis. PLoS One. 2019;14(12):e0226637. Published 2019 Dec 27. doi:10.1371/journal.pone.0226637 | NRSIs included |
| 1. Zabaleta-Del-Olmo E, Vlacho B, Jodar-Fernández L, et al. Safety of the reuse of needles for subcutaneous insulin injection: A systematic review and meta-analysis. Int J Nurs Stud. 2016;60:121-132. doi:10.1016/j.ijnurstu.2016.04.010 | NRSIs included |
| 1. Zhang Y, Ma L. Effect of preoperative angiotensin-converting enzyme inhibitor on the outcome of coronary artery bypass graft surgery. Eur J Cardiothorac Surg. 2015;47(5):788-795. doi:10.1093/ejcts/ezu298 | NRSIs included |
| 1. Zhao X, Wu X, Dong J, Liu Y, Zheng L, Zhang L. A Meta-analysis of Postoperative Complications of Tissue Expander/Implant Breast Reconstruction Using Acellular Dermal Matrix. Aesthetic Plast Surg. 2015;39(6):892-901. doi:10.1007/s00266-015-0555-z | NRSIs included |
| 1. Zhu Q, Hu H, Weng DS, et al. Pooled safety analyses of ALK-TKI inhibitor in ALK-positive NSCLC. BMC Cancer. 2017;17(1):412. Published 2017 Jun 12. doi:10.1186/s12885-017-3405-3 | NRSIs included, no 2 by 2 table data |
| 1. Zhu J, Liao R, Su C, Liang D, Wu J, Qiu K, Li J. Toxicity profile characteristics of novel androgen-deprivation therapy agents in patients with prostate cancer: a meta-analysis. Expert Rev Anticancer Ther. 2018 Feb;18(2):193-198. | NRSIs included |
| 1. Abdel-Rahman O, Fouad M. A network meta-analysis of the risk of immune-related renal toxicity in cancer patients treated with immune checkpoint inhibitors. Immunotherapy. 2016;8(5):665-674. doi:10.2217/imt-2015-0020 | NMA, without pairwise MA |
| 1. Abdel-Rahman O, Oweira H, Petrausch U, et al. Immune-related ocular toxicities in solid tumor patients treated with immune checkpoint inhibitors: a systematic review. Expert Rev Anticancer Ther. 2017;17(4):387-394. doi:10.1080/14737140.2017.1296765 | Less than 5 studies |
| 1. Ahmed AM, Moahammed AT, Mattar OM, et al. Surgical treatment of diverticulitis and its complications: A systematic review and meta-analysis of randomized control trials. Surgeon. 2018;16(6):372-383. doi:10.1016/j.surge.2018.03.011 | Less than 5 studies |
| 1. Alfayez OM, Al Yami MS, Alshibani M, et al. Network meta-analysis of nine large cardiovascular outcome trials of new antidiabetic drugs. Prim Care Diabetes. 2019;13(3):204-211. doi:10.1016/j.pcd.2019.01.003 | NMA, without pairwise MA |
| 1. Alfayez OM, Almutairi AR, Aldosari A, Al Yami MS. Update on Cardiovascular Safety of Incretin-Based Therapy in Adults With Type 2 Diabetes Mellitus: A Meta-Analysis of Cardiovascular Outcome Trials. Can J Diabetes. 2019;43(7):538-545.e2. doi:10.1016/j.jcjd.2019.04.003 | No reference for included studies |
| 1. Alves C, Ribeiro I, Penedones A, Mendes D, Batel Marques F. Risk of Ophthalmic Adverse Effects in Patients Treated with MEK Inhibitors: A Systematic Review and Meta-Analysis. Ophthalmic Res. 2017;57(1):60-69. doi:10.1159/000446845 | No 2 by 2 table data |
| 1. Andersen SE, Christensen M. Hypoglycaemia when adding sulphonylurea to metformin: a systematic review and network meta-analysis. Br J Clin Pharmacol. 2016;82(5):1291-1302. doi:10.1111/bcp.13059 | NMA, without pairwise MA |
| 1. Ando T, Ashraf S, Villablanca PA, et al. Meta-Analysis Comparing the Incidence of Infective Endocarditis Following Transcatheter Aortic Valve Implantation Versus Surgical Aortic Valve Replacement. Am J Cardiol. 2019;123(5):827-832. doi:10.1016/j.amjcard.2018.11.031 | Less than 5 studies |
| 1. Andreou I, Briasoulis A, Pappas C, Ikonomidis I, Alexopoulos D. Ticagrelor Versus Clopidogrel as Part of Dual or Triple Antithrombotic Therapy: a Systematic Review and Meta-Analysis. Cardiovasc Drugs Ther. 2018;32(3):287-294. doi:10.1007/s10557-018-6795-9 | Less than 5 studies |
| 1. Avery RL, Gordon GM. Systemic Safety of Prolonged Monthly Anti-Vascular Endothelial Growth Factor Therapy for Diabetic Macular Edema: A Systematic Review and Meta-analysis. JAMA Ophthalmol. 2016;134(1):21-29. doi:10.1001/jamaophthalmol.2015.4070 | Less than 5 studies |
| 1. Baay M, Bollaerts K, Verstraeten T. A systematic review and meta-analysis on the safety of newly adjuvanted vaccines among older adults. Vaccine. 2018;36(29):4207-4214. doi:10.1016/j.vaccine.2018.06.004 | NRSIs included |
| 1. Bakheet MF, Pearce LA, Hart RG. Effect of addition of clopidogrel to aspirin on subdural hematoma: meta-analysis of randomized clinical trials. Int J Stroke. 2015;10(4):501-505. doi:10.1111/ijs.12419 | Less than 5 studies |
| 1. Bannuru RR, Osani M, Vaysbrot EE, McAlindon TE. Comparative safety profile of hyaluronic acid products for knee osteoarthritis: a systematic review and network meta-analysis. Osteoarthritis Cartilage. 2016;24(12):2022-2041. doi:10.1016/j.joca.2016.07.010 | NMA, without pairwise MA |
| 1. Bell S, Rennie T, Marwick CA, Davey P. Effects of peri-operative nonsteroidal anti-inflammatory drugs on post-operative kidney function for adults with normal kidney function. Cochrane Database Syst Rev. 2018;11(11):CD011274. Published 2018 Nov 29. doi:10.1002/14651858.CD011274.pub2 | Continuous outcomes |
| 1. Belum VR, Serna-Tamayo C, Wu S, Lacouture ME. Incidence and risk of hand-foot skin reaction with cabozantinib, a novel multikinase inhibitor: a meta-analysis. Clin Exp Dermatol. 2016;41(1):8-15. doi:10.1111/ced.12694 | No 2 by 2 table data |
| 1. Bertrand A, Kostine M, Barnetche T, Truchetet ME, Schaeverbeke T. Immune related adverse events associated with anti-CTLA-4 antibodies: systematic review and meta-analysis. BMC Med. 2015;13:211. Published 2015 Sep 4. doi:10.1186/s12916-015-0455-8 | Less than 5 studies |
| 1. Bonovas S, Fiorino G, Allocca M, et al. Biologic Therapies and Risk of Infection and Malignancy in Patients With Inflammatory Bowel Disease: A Systematic Review and Network Meta-analysis. Clin Gastroenterol Hepatol. 2016;14(10):1385-1397.e10. doi:10.1016/j.cgh.2016.04.039 | NMA, without pairwise MA |
| 1. Bonovas S, Nikolopoulos GK, Lytras T, Fiorino G, Peyrin-Biroulet L, Danese S. Comparative safety of systemic and low-bioavailability steroids in inflammatory bowel disease: Systematic review and network meta-analysis. Br J Clin Pharmacol. 2018;84(2):239-251. doi:10.1111/bcp.13456 | NMA, without pairwise MA |
| 1. Brotis AG, Tasiou A, Paterakis K, Tzerefos C, Fountas KN. Complications Associated with Surgery for Thoracic Disc Herniation: A Systematic Review and Network Meta-Analysis. World Neurosurg. 2019;132:334-342. doi:10.1016/j.wneu.2019.08.202 | NMA, without pairwise MA |
| 1. Buller M, Schulz S, Kasdan M, Wilhelmi BJ. The Incidence of Complex Regional Pain Syndrome in Simultaneous Surgical Treatment of Carpal Tunnel Syndrome and Dupuytren Contracture. Hand (N Y). 2018;13(4):391-394. doi:10.1177/1558944717718345 | Less than 5 studies |
| 1. Caldeira D, Gonçalves N, Pinto FJ, Costa J, Ferreira JJ. Risk of renal failure with the non-vitamin K antagonist oral anticoagulants: systematic review and meta-analysis. Pharmacoepidemiol Drug Saf. 2015;24(7):757-764. doi:10.1002/pds.3791 | No 2 by 2 table data |
| 1. Cao M, Li F, Wang Y, Zhang J. Treatment-related serious adverse events and fatal adverse events with regorafenib in cancer patients: a meta-analysis of phase 3 randomized controlled trials. Invest New Drugs. 2017;35(6):834-838. doi:10.1007/s10637-017-0512-6 | Less than 5 studies |
| 1. Cavero-Redondo I, Álvarez-Bueno C, Pozuelo-Carrascosa DP, Díez-Fernández A, Notario-Pacheco B. Risk of extrapyramidal side effects comparing continuous vs. bolus intravenous metoclopramide administration: a systematic review and meta-analysis of randomised controlled trials. J Clin Nurs. 2015;24(23-24):3638-3646. doi:10.1111/jocn.12984 | No 2 by 2 table data |
| 1. Chang CH, Tseng PT, Chen NY, et al. Safety and tolerability of prescription omega-3 fatty acids: A systematic review and meta-analysis of randomized controlled trials. Prostaglandins Leukot Essent Fatty Acids. 2018;129:1-12. doi:10.1016/j.plefa.2018.01.001 | No 2 by 2 table data |
| 1. Chierrito de Oliveira D, Guerrero de Sousa P, Borges Dos Reis C, et al. Safety of Treatments for ADHD in Adults: Pairwise and Network Meta-Analyses. J Atten Disord. 2019;23(2):111-120. doi:10.1177/1087054717696773 | NMA, without pairwise MA |
| 1. Chou CY, Chang YT, Yang JL, et al. Effect of Long-term Incretin-Based Therapies on Ischemic Heart Diseases in Patients with Type 2 Diabetes Mellitus: A Network Meta-analysis. Sci Rep. 2017;7(1):15795. Published 2017 Nov 17. doi:10.1038/s41598-017-16101-1 | NMA, without pairwise MA |
| 1. Corona G, Tirabassi G, Santi D, et al. Sexual dysfunction in subjects treated with inhibitors of 5α-reductase for benign prostatic hyperplasia: a comprehensive review and meta-analysis. Andrology. 2017;5(4):671-678. doi:10.1111/andr.12353 | Less than 5 studies |
| 1. Costa APF, Cobucci RNO, da Silva JM, da Costa Lima PH, Giraldo PC, Gonçalves AK. Safety of Human Papillomavirus 9-Valent Vaccine: A Meta-Analysis of Randomized Trials. J Immunol Res. 2017;2017:3736201. doi:10.1155/2017/3736201 | Less than 5 studies |
| 1. Cramer H, Ward L, Saper R, Fishbein D, Dobos G, Lauche R. The Safety of Yoga: A Systematic Review and Meta-Analysis of Randomized Controlled Trials. Am J Epidemiol. 2015;182(4):281-293. doi:10.1093/aje/kwv071 | No 2 by 2 table data |
| 1. Curtis E, Fuggle N, Shaw S, et al. Safety of Cyclooxygenase-2 Inhibitors in Osteoarthritis: Outcomes of a Systematic Review and Meta-Analysis. Drugs Aging. 2019;36(Suppl 1):25-44. doi:10.1007/s40266-019-00664-x | No 2 by 2 table data |
| 1. de Carvalho ALR, Vital RB, de Lira CCS, et al. Laryngeal Mask Airway Versus Other Airway Devices for Anesthesia in Children With an Upper Respiratory Tract Infection: A Systematic Review and Meta-analysis of Respiratory Complications [published correction appears in Anesth Analg. 2019 Feb;128(2):e37]. Anesth Analg. 2018;127(4):941-950. doi:10.1213/ANE.0000000000003674 | Less than 5 studies |
| 1. Demiri M, Antunes T, Fletcher D, Martinez V. Perioperative adverse events attributed to α2-adrenoceptor agonists in patients not at risk of cardiovascular events: systematic review and meta-analysis. Br J Anaesth. 2019;123(6):795-807. doi:10.1016/j.bja.2019.07.029 | No 2 by 2 table data |
| 1. Ding PN, Lord SJ, Gebski V, et al. Risk of Treatment-Related Toxicities from EGFR Tyrosine Kinase Inhibitors: A Meta-analysis of Clinical Trials of Gefitinib, Erlotinib, and Afatinib in Advanced EGFR-Mutated Non-Small Cell Lung Cancer. J Thorac Oncol. 2017;12(4):633-643. doi:10.1016/j.jtho.2016.11.2236 | No reference for included studies |
| 1. Ding SX, Chen T, Wang T, Liu CY, Lu WL, Fu R. The Risk of Clonal Evolution of Granulocyte Colony-Stimulating Factor for Acquired Aplastic Anemia: A Systematic Review and Meta-Analysis. Acta Haematol. 2018;140(3):141-145. doi:10.1159/000491816 | Less than 5 studies |
| 1. Dinges HC, Otto S, Stay DK, et al. Side Effect Rates of Opioids in Equianalgesic Doses via Intravenous Patient-Controlled Analgesia: A Systematic Review and Network Meta-analysis. Anesth Analg. 2019;129(4):1153-1162. doi:10.1213/ANE.0000000000003887 | NMA, without pairwise MA |
| 1. Donegan S, Dixon P, Hemming K, Tudur-Smith C, Marson A. A systematic review of placebo-controlled trials of topiramate: How useful is a multiple-indications review for evaluating the adverse events of an antiepileptic drug?. Epilepsia. 2015;56(12):1910-1920. doi:10.1111/epi.13209 | No 2 by 2 table data |
| 1. Dorjee P, Long ZW. A mixed treatment comparison of toxicity of gemcitabine combined with different targeted drugs in the treatment of advanced or metastatic pancreatic cancer. Cancer Biol Ther. 2018;19(6):497-506. doi:10.1080/15384047.2018.1433503 | NMA, without pairwise MA |
| 1. Du CR, Ying HM, Kong FF, Zhai RP, Hu CS. Concurrent chemoradiotherapy was associated with a higher severe late toxicity rate in nasopharyngeal carcinoma patients compared with radiotherapy alone: a meta-analysis based on randomized controlled trials. Radiat Oncol. 2015;10:70. Published 2015 Mar 26. doi:10.1186/s13014-015-0377-9 | No 2 by 2 table data |
| 1. Duarte GS, Rodrigues FB, Ferreira JJ, Costa J. Adverse events with botulinum toxin treatment in cervical dystonia: How much should we blame placebo?. Parkinsonism Relat Disord. 2018;56:16-19. doi:10.1016/j.parkreldis.2018.06.017 | No 2 by 2 table data |
| 1. Egunsola O, Choonara I, Sammons HM. Safety of lamotrigine in paediatrics: a systematic review. BMJ Open. 2015;5(6):e007711. Published 2015 Jun 12. doi:10.1136/bmjopen-2015-007711 | Less than 5 studies |
| 1. Elwood PC, Morgan G, Galante J, et al. Systematic Review and Meta-Analysis of Randomised Trials to Ascertain Fatal Gastrointestinal Bleeding Events Attributable to Preventive Low-Dose Aspirin: No Evidence of Increased Risk. PLoS One. 2016;11(11):e0166166. Published 2016 Nov 15. doi:10.1371/journal.pone.0166166 | No 2 by 2 table data |
| 1. Fabisiak A, Włodarczyk M, Fabisiak N, Storr M, Fichna J. Gastrointestinal Adverse Events of Cannabinoid 1 Receptor Inverse Agonists suggest their Potential Use in Irritable Bowel Syndrome with Constipation: A Systematic Review and Meta-Analysis. J Gastrointestin Liver Dis. 2019;28(4):473-481. Published 2019 Dec 9. doi:10.15403/jgld-265 | No 2 by 2 table data |
| 1. Favilla V, Russo GI, Privitera S, et al. Impact of combination therapy 5-alpha reductase inhibitors (5-ARI) plus alpha-blockers (AB) on erectile dysfunction and decrease of libido in patients with LUTS/BPH: a systematic review with meta-analysis. Aging Male. 2016;19(3):175-181. doi:10.1080/13685538.2016.1195361 | Less than 5 studies |
| 1. Ferreira VL, Assis Jarek NA, Tonin FS, Borba HH, Wiens A, Pontarolo R. Safety of interferon-free therapies for chronic hepatitis C: a network meta-analysis. J Clin Pharm Ther. 2016;41(5):478-485. doi:10.1111/jcpt.12426 | NMA, without pairwise MA |
| 1. Fogacci F, Banach M, Mikhailidis DP, et al. Safety of red yeast rice supplementation: A systematic review and meta-analysis of randomized controlled trials. Pharmacol Res. 2019;143:1-16. doi:10.1016/j.phrs.2019.02.028 | No 2 by 2 table data |
| 1. Ford N, Shubber Z, Pozniak A, et al. Comparative Safety and Neuropsychiatric Adverse Events Associated With Efavirenz Use in First-Line Antiretroviral Therapy: A Systematic Review and Meta-Analysis of Randomized Trials. J Acquir Immune Defic Syndr. 2015;69(4):422-429. doi:10.1097/QAI.0000000000000606 | No 2 by 2 table data |
| 1. Fuggle N, Curtis E, Shaw S, et al. Safety of Opioids in Osteoarthritis: Outcomes of a Systematic Review and Meta-Analysis. Drugs Aging. 2019;36(Suppl 1):129-143. doi:10.1007/s40266-019-00666-9 | No 2 by 2 table data |
| 1. Furuya-Kanamori L, Doi SA, Onitilo A, Akhtar S. Is there truly an increase in risk of cardiovascular and hematological adverse events with vascular endothelial growth factor receptor tyrosine kinase inhibitors?. Expert Opin Drug Saf. 2020;19(2):223-228. doi:10.1080/14740338.2020.1691167 | No 2 by 2 table data |
| 1. Gaffar R, Habib B, Filion KB, Reynier P, Eisenberg MJ. Optimal Timing of Complete Revascularization in Acute Coronary Syndrome: A Systematic Review and Meta-Analysis. J Am Heart Assoc. 2017;6(4):e005381. Published 2017 Apr 10. doi:10.1161/JAHA.116.005381 | Less than 5 studies |
| 1. Galling B, Roldán A, Rietschel L, et al. Safety and tolerability of antipsychotic co-treatment in patients with schizophrenia: results from a systematic review and meta-analysis of randomized controlled trials. Expert Opin Drug Saf. 2016;15(5):591-612. doi:10.1517/14740338.2016.1165668 | No 2 by 2 table data |
| 1. Gao C, Wang J, Li Y, et al. Incidence and risk of hematologic toxicities with hypomethylating agents in the treatment of myelodysplastic syndromes and acute myeloid leukopenia: A systematic review and meta-analysis. Medicine (Baltimore). 2018;97(34):e11860. doi:10.1097/MD.0000000000011860 | Wrong references for included studies, less than 5 studies |
| 1. Ghumman SS, Weinerman J, Khan A, et al. Contrast induced-acute kidney injury following peripheral angiography with carbon dioxide versus iodinated contrast media: A meta-analysis and systematic review of current literature. Catheter Cardiovasc Interv. 2017;90(3):437-448. doi:10.1002/ccd.27051 | No 2 by 2 table data |
| 1. Giacoppo D, Colleran R, Cassese S, et al. Percutaneous Coronary Intervention vs Coronary Artery Bypass Grafting in Patients With Left Main Coronary Artery Stenosis: A Systematic Review and Meta-analysis. JAMA Cardiol. 2017;2(10):1079-1088. doi:10.1001/jamacardio.2017.2895 | No 2 by 2 table data |
| 1. Giugliano D, Maiorino MI, Longo M, Bellastella G, Chiodini P, Esposito K. Type 2 diabetes and risk of heart failure: a systematic review and meta-analysis from cardiovascular outcome trials. Endocrine. 2019;65(1):15-24. doi:10.1007/s12020-019-01931-y | No 2 by 2 table data |
| 1. Gong W, Li A, Ai H, Shi H, Wang X, Nie S. Safety of early discharge after primary angioplasty in low-risk patients with ST-segment elevation myocardial infarction: A meta-analysis of randomised controlled trials. Eur J Prev Cardiol. 2018;25(8):807-815. doi:10.1177/2047487318763823 | Less than 5 studies |
| 1. Guo S, Chen L, Cheng S, Xu H. Comparative cardiovascular safety of selective serotonin reuptake inhibitors (SSRIs) among Chinese senile depression patients: A network meta-analysis of randomized controlled trials. Medicine (Baltimore). 2019;98(22):e15786. doi:10.1097/MD.0000000000015786 | NMA, without pairwise MA |
| 1. Hou LQ, Jiang GX, Chen YF, et al. The Comparative Safety of TNF Inhibitors in Ankylosing Spondylitis-a Meta-Analysis Update of 14 Randomized Controlled Trials. Clin Rev Allergy Immunol. 2018;54(2):234-243. doi:10.1007/s12016-017-8623-6 | No 2 by 2 table data |
| 1. Huang Y, Fan H, Li N, Du J. Risk of immune-related pneumonitis for PD1/PD-L1 inhibitors: Systematic review and network meta-analysis. Cancer Med. 2019;8(5):2664-2674. doi:10.1002/cam4.2104 | NMA, without pairwise MA |
| 1. Huang F, Luo ZC. Risk of Adverse Drug Events Observed with Baricitinib 2 mg Versus Baricitinib 4 mg Once Daily for the Treatment of Rheumatoid Arthritis: A Systematic Review and Meta-Analysis of Randomized Controlled Trials. BioDrugs. 2018 Oct;32(5):415-423. | Less than 5 studies |
| 1. Isomura T, Suzuki S, Origasa H, et al. Liver-related safety assessment of green tea extracts in humans: a systematic review of randomized controlled trials [published correction appears in Eur J Clin Nutr. 2016 Nov;70(11):1340]. Eur J Clin Nutr. 2016;70(11):1221-1229. doi:10.1038/ejcn.2016.78 | Less than 5 studies |
| 1. Karyotaki E, Kemmeren L, Riper H, et al. Is self-guided internet-based cognitive behavioural therapy (iCBT) harmful? An individual participant data meta-analysis. Psychol Med. 2018;48(15):2456-2466. doi:10.1017/S0033291718000648 | No 2 by 2 table data |
| 1. Kassem L, Shohdy KS, Lasheen S, Abdel-Rahman O, Bachelot T. Hematological adverse effects in breast cancer patients treated with cyclin-dependent kinase 4 and 6 inhibitors: a systematic review and meta-analysis. Breast Cancer. 2018;25(1):17-27. doi:10.1007/s12282-017-0818-4 | Less than 5 studies |
| 1. Katsanos AH, Schellinger PD, Köhrmann M, et al. Fatal oral anticoagulant-related intracranial hemorrhage: a systematic review and meta-analysis. Eur J Neurol. 2018;25(10):1299-1302. doi:10.1111/ene.13742 | Less than 5 studies |
| 1. Kirby A, Hobson RP, Burke D, Cleveland V, Ford G, West RM. Appendicectomy for suspected uncomplicated appendicitis is associated with fewer complications than conservative antibiotic management: a meta-analysis of post-intervention complications. J Infect. 2015;70(2):105-110. doi:10.1016/j.jinf.2014.08.009 | Less than 5 studies |
| 1. Kötter T, da Costa BR, Fässler M, et al. Metamizole-associated adverse events: a systematic review and meta-analysis. PLoS One. 2015;10(4):e0122918. Published 2015 Apr 13. doi:10.1371/journal.pone.0122918 | No 2 by 2 table data |
| 1. la Chapelle CF, Swank HA, Wessels ME, Mol BW, Rubinstein SM, Jansen FW. Trocar types in laparoscopy. Cochrane Database Syst Rev. 2015;(12):CD009814. Published 2015 Dec 16. doi:10.1002/14651858.CD009814.pub2 | Less than 5 studies |
| 1. Lasheen S, Shohdy KS, Kassem L, Abdel-Rahman O. Fatigue, alopecia and stomatitis among patients with breast cancer receiving cyclin-dependent kinase 4 and 6 inhibitors: a systematic review and meta-analysis. Expert Rev Anticancer Ther. 2017;17(9):851-856. doi:10.1080/14737140.2017.1355242 | Less than 5 studies |
| 1. Li BD, Bi ZY, Liu JF, et al. Adverse effects produced by different drugs used in the treatment of Parkinson's disease: A mixed treatment comparison. CNS Neurosci Ther. 2017;23(10):827-842. doi:10.1111/cns.12727 | NMA, without pairwise MA |
| 1. Li C, Cheng W, Guo J, Guan W. Relationship of inhaled long-acting bronchodilators with cardiovascular outcomes among patients with stable COPD: a meta-analysis and systematic review of 43 randomized trials. Int J Chron Obstruct Pulmon Dis. 2019;14:799-808. Published 2019 Apr 11. doi:10.2147/COPD.S198288 | No 2 by 2 table data |
| 1. Li D, Yang JY, Wang T, Shen S, Tang H. Risks of diabetic foot syndrome and amputation associated with sodium glucose co-transporter 2 inhibitors: A Meta-analysis of Randomized Controlled Trials. Diabetes Metab. 2018;44(5):410-414. doi:10.1016/j.diabet.2018.02.001 | Less than 5 studies |
| 1. Li L, Li Y, Xu X, et al. Safety evaluation on low-molecular-weight hydroxyethyl starch for volume expansion therapy in pediatric patients: a meta-analysis of randomized controlled trials. Crit Care. 2015;19(1):79. Published 2015 Mar 10. doi:10.1186/s13054-015-0815-y | Less than 5 studies |
| 1. Li X, Huang R, Xu Z. Risk of Adverse Vascular Events in Newly Diagnosed Glioblastoma Multiforme Patients Treated with Bevacizumab: a Systematic Review and Meta-Analysis. Sci Rep. 2015;5:14698. Published 2015 Oct 1. doi:10.1038/srep14698 | Less than 5 studies |
| 1. Ma W, Xu M, Liu Y, et al. Safety profile of combined therapy inhibiting EFGR and VEGF pathways in patients with advanced non-small-cell lung cancer: A meta-analysis of 15 phase II/III randomized trials. Int J Cancer. 2015;137(2):409-419. doi:10.1002/ijc.29377 | No 2 by 2 table data |
| 1. Mannucci E, Monami M. Cardiovascular Safety of Incretin-Based Therapies in Type 2 Diabetes: Systematic Review of Integrated Analyses and Randomized Controlled Trials. Adv Ther. 2017;34(1):1-40. doi:10.1007/s12325-016-0432-4 | No 2 by 2 table data |
| 1. Manohar S, Kompotiatis P, Thongprayoon C, Cheungpasitporn W, Herrmann J, Herrmann SM. Programmed cell death protein 1 inhibitor treatment is associated with acute kidney injury and hypocalcemia: meta-analysis. Nephrol Dial Transplant. 2019;34(1):108-117. doi:10.1093/ndt/gfy105 | No 2 by 2 table data |
| 1. Massey PR, Okman JS, Wilkerson J, Cowen EW. Tyrosine kinase inhibitors directed against the vascular endothelial growth factor receptor (VEGFR) have distinct cutaneous toxicity profiles: a meta-analysis and review of the literature. Support Care Cancer. 2015;23(6):1827-1835. doi:10.1007/s00520-014-2520-9 | No 2 by 2 table data |
| 1. Mazaud C, Fardet L. Relative risk of and determinants for adverse events of methotrexate prescribed at a low dose: a systematic review and meta-analysis of randomized placebo-controlled trials. Br J Dermatol. 2017;177(4):978-986. doi:10.1111/bjd.15377 | No 2 by 2 table data |
| 1. Meister R, von Wolff A, Mohr H, et al. Comparative Safety of Pharmacologic Treatments for Persistent Depressive Disorder: A Systematic Review and Network Meta-Analysis. PLoS One. 2016;11(5):e0153380. Published 2016 May 17. doi:10.1371/journal.pone.0153380 | NMA, without pairwise MA |
| 1. Moćko P, Kawalec P, Pilc A. Safety Profile of Biologic Drugs in the Therapy of Ulcerative Colitis: A Systematic Review and Network Meta-Analysis. Pharmacotherapy. 2016;36(8):870-879. doi:10.1002/phar.1785 | NMA, without pairwise MA |
| 1. Moćko P, Kawalec P, Pilc A. Safety profile of biologic drugs in the therapy of Crohn disease: A systematic review and network meta-analysis. Pharmacol Rep. 2016;68(6):1237-1243. doi:10.1016/j.pharep.2016.07.013 | NMA, without pairwise MA |
| 1. Moćko P, Kawalec P, Pilc A. Safety Profile of Biologic Drugs in the Treatment of Inflammatory Bowel Diseases: A Systematic Review and Network Meta-analysis of Randomized Controlled Trials. Clin Drug Investig. 2017;37(1):25-37. doi:10.1007/s40261-016-0459-y | NMA, without pairwise MA |
| 1. Monami M, Nreu B, Scatena A, et al. Safety issues with glucagon-like peptide-1 receptor agonists (pancreatitis, pancreatic cancer and cholelithiasis): Data from randomized controlled trials. Diabetes Obes Metab. 2017;19(9):1233-1241. doi:10.1111/dom.12926 | No 2 by 2 table data |
| 1. Monami M, Nreu B, Zannoni S, Lualdi C, Mannucci E. Effects of SGLT-2 inhibitors on diabetic ketoacidosis: A meta-analysis of randomised controlled trials. Diabetes Res Clin Pract. 2017;130:53-60. doi:10.1016/j.diabres.2017.04.017 | No 2 by 2 table data |
| 1. Moreira R.B., de Biasi M., Francini E., Nuzzo P.V., de Velasco G., Maluf F.C., Fay A.P., Bellmunt J., Choueiri T.K., Schutz F.A. Differential side effects profile in patients with mCRPC treated with abiraterone or enzalutamide: A meta-analysis of randomized controlled trials. Oncotarget. 2017;8:84572–84578. doi: 10.18632/oncotarget.20028. | Less than 5 studies |
| 1. Neto AS, Hemmes SN, Barbas CS, et al. Association between driving pressure and development of postoperative pulmonary complications in patients undergoing mechanical ventilation for general anaesthesia: a meta-analysis of individual patient data [published correction appears in Lancet Respir Med. 2016 Jun;4(6):e34]. Lancet Respir Med. 2016;4(4):272-280. doi:10.1016/S2213-2600(16)00057-6 | No 2 by 2 table data |
| 1. Nishijima TF, Shachar SS, Nyrop KA, Muss HB. Safety and Tolerability of PD-1/PD-L1 Inhibitors Compared with Chemotherapy in Patients with Advanced Cancer: A Meta-Analysis. Oncologist. 2017;22(4):470-479. doi:10.1634/theoncologist.2016-0419 | No 2 by 2 table data |
| 1. Pan XB, Huang ST, Jiang YM, Ma JL, Zhu XD. Secondary malignancies after partial versus whole breast irradiation: a systematic review and meta-analysis. Oncotarget. 2016;7(44):71951-71959. doi:10.18632/oncotarget.12442 | Less than 5 studies |
| 1. Pan Y, Hu C, Chen PH, et al. Association of oral endothelin receptor antagonists with risks of cardiovascular events and mortality: meta-analysis of randomized controlled trials. Eur J Clin Pharmacol. 2017;73(3):267-278. doi:10.1007/s00228-016-2171-5 | No 2 by 2 table data |
| 1. Pavlova V, Filipova E, Uzunova K, Kalinov K, Vekov T. Pioglitazone Therapy and Fractures: Systematic Review and Meta- Analysis. Endocr Metab Immune Disord Drug Targets. 2018;18(5):502-507. doi:10.2174/1871530318666180423121833 | No 2 by 2 table data |
| 1. Peng L, Bu Z, Ye X, Zhou Y, Zhao Q. Incidence and risk of peripheral neuropathy with nab-paclitaxel in patients with cancer: a meta-analysis. Eur J Cancer Care (Engl). 2017;26(5):10.1111/ecc.12407. doi:10.1111/ecc.12407 | No 2 by 2 table data |
| 1. Peng L, Ye X, Zhou Y, Zhang J, Zhao Q. Meta-analysis of incidence and risk of peripheral neuropathy associated with intravenous bortezomib. Support Care Cancer. 2015;23(9):2813-2824. doi:10.1007/s00520-015-2648-2 | No 2 by 2 table data |
| 1. Peng L, Zhou Y, Ye X, Zhao Q. Treatment-related fatigue with everolimus and temsirolimus in patients with cancer-a meta-analysis of clinical trials. Tumour Biol. 2015;36(2):643-654. doi:10.1007/s13277-014-2669-3 | No 2 by 2 table data |
| 1. Petrelli F, Ardito R, Ghidini A, et al. Different Toxicity of Cetuximab and Panitumumab in Metastatic Colorectal Cancer Treatment: A Systematic Review and Meta-Analysis. Oncology. 2018;94(4):191-199. doi:10.1159/000486338 | No 2 by 2 table data |
| 1. Qi WX, Fu S, Zhang Q, Guo XM. Incidence and risk of hypertension associated with ramucirumab in cancer patients: A systematic review and meta-analysis. J Cancer Res Ther. 2016;12(2):775-781. doi:10.4103/0973-1482.148700 | No 2 by 2 table data |
| 1. Qu CP, Sun GX, Yang SQ, Tian J, Si JG, Wang YF. Toxicities of different first-line chemotherapy regimens in the treatment of advanced ovarian cancer: A network meta-analysis. Medicine (Baltimore). 2017;96(2):e5797. doi:10.1097/MD.0000000000005797 | NMA, without pairwise MA |
| 1. Rahouma M, Karim NA, Baudo M, et al. Cardiotoxicity with immune system targeting drugs: a meta-analysis of anti-PD/PD-L1 immunotherapy randomized clinical trials. Immunotherapy. 2019;11(8):725-735. doi:10.2217/imt-2018-0118 | No 2 by 2 table data |
| 1. Rogliani P, Matera MG, Ora J, Cazzola M, Calzetta L. The impact of dual bronchodilation on cardiovascular serious adverse events and mortality in COPD: a quantitative synthesis [published correction appears in Int J Chron Obstruct Pulmon Dis. 2018 Oct 31;13:3597]. Int J Chron Obstruct Pulmon Dis. 2017;12:3469-3485. Published 2017 Dec 5. doi:10.2147/COPD.S146338 | NMA, without pairwise MA |
| 1. Roviello G, Sigala S, Danesi R, et al. Incidence and relative risk of adverse events of special interest in patients with castration resistant prostate cancer treated with CYP-17 inhibitors: A meta-analysis of published trials. Crit Rev Oncol Hematol. 2016;101:12-20. doi:10.1016/j.critrevonc.2016.02.013 | Less than 5 studies |
| 1. Ruzieh M, Moroi MK, Aboujamous NM, et al. Meta-Analysis Comparing the Relative Risk of Adverse Events for Amiodarone Versus Placebo. Am J Cardiol. 2019;124(12):1889-1893. doi:10.1016/j.amjcard.2019.09.008 | No 2 by 2 table data |
| 1. Salas PAO, Parra CO, Florez CEP, Goez LM, Velez-van-Meerbeke A, Rodriguez JH. Safety liver profile of teriflunomide versus interferon β in multiple sclerosis: Systematic review and indirect comparison meta-analysis. Mult Scler Relat Disord. 2018;26:192-200. doi:10.1016/j.msard.2018.09.014 | No 2 by 2 table data |
| 1. Schneider-Thoma J, Efthimiou O, Bighelli I, et al. Second-generation antipsychotic drugs and short-term somatic serious adverse events: a systematic review and meta-analysis. Lancet Psychiatry. 2019;6(9):753-765. doi:10.1016/S2215-0366(19)30223-8 | No 2 by 2 table data |
| 1. Schneider-Thoma J, Efthimiou O, Huhn M, et al. Second-generation antipsychotic drugs and short-term mortality: a systematic review and meta-analysis of placebo-controlled randomised controlled trials. Lancet Psychiatry. 2018;5(8):653-663. doi:10.1016/S2215-0366(18)30177-9 | No 2 by 2 table data |
| 1. Schulman AR, Popov V, Thompson CC. Randomized sham-controlled trials in endoscopy: a systematic review and meta-analysis of adverse events. Gastrointest Endosc. 2017;86(6):972-985.e3. doi:10.1016/j.gie.2017.07.046 | No 2 by 2 table data |
| 1. Shang H, Zhang Z, Feng A, et al. The overall safety evaluation of programmed cell death/programmed cell death ligand 1 (PD-1/PD-L1) treatment for lung cancer patients: An updated systematic review and meta-analysis. Medicine (Baltimore). 2019;98(30):e16439. doi:10.1097/MD.0000000000016439 | Less than 5 studies |
| 1. Shen Z, Kong D. Meta-analysis of the adverse events associated with extended-release versus standard immediate-release pramipexole in Parkinson disease. Medicine (Baltimore). 2018;97(34):e11316. doi:10.1097/MD.0000000000011316 | Less than 5 studies |
| 1. Shi XH, Zhou X, Zhang YM, Lei ZY, Liu T, Fan DL. Complications from Nasolabial Fold Injection of Calcium Hydroxylapatite for Facial Soft-Tissue Augmentation: A Systematic Review and Meta-Analysis. Aesthet Surg J. 2016;36(6):712-717. doi:10.1093/asj/sjv206 | Less than 5 studies |
| 1. Siemieniuk RA, Foroutan F, Mirza R, et al. Antiretroviral therapy for pregnant women living with HIV or hepatitis B: a systematic review and meta-analysis. BMJ Open. 2017;7(9):e019022. Published 2017 Sep 11. doi:10.1136/bmjopen-2017-019022 | Less than 5 studies |
| 1. Singh JA, Cameron C, Noorbaloochi S, et al. Risk of serious infection in biological treatment of patients with rheumatoid arthritis: a systematic review and meta-analysis. Lancet. 2015;386(9990):258-265. doi:10.1016/S0140-6736(14)61704-9 | NMA, without pairwise MA |
| 1. Singh JA, Hossain A, Kotb A, Wells G. Risk of serious infections with immunosuppressive drugs and glucocorticoids for lupus nephritis: a systematic review and network meta-analysis. BMC Med. 2016;14(1):137. Published 2016 Sep 13. doi:10.1186/s12916-016-0673-8 | NMA, without pairwise MA |
| 1. Sirker A, Kwok CS, Kotronias R, et al. Influence of access site choice for cardiac catheterization on risk of adverse neurological events: A systematic review and meta-analysis. Am Heart J. 2016;181:107-119. doi:10.1016/j.ahj.2016.06.027 | Less than 5 studies |
| 1. Sobieraj DM, Martinez BK, Hernandez AV, et al. Adverse Effects of Pharmacologic Treatments of Major Depression in Older Adults. J Am Geriatr Soc. 2019;67(8):1571-1581. doi:10.1111/jgs.15966 | No 2 by 2 table data |
| 1. Stassijns J, Bollaerts K, Baay M, Verstraeten T. A systematic review and meta-analysis on the safety of newly adjuvanted vaccines among children. Vaccine. 2016;34(6):714-722. doi:10.1016/j.vaccine.2015.12.024 | NRSIs included |
| 1. Storgaard H, Cold F, Gluud LL, Vilsbøll T, Knop FK. Glucagon-like peptide-1 receptor agonists and risk of acute pancreatitis in patients with type 2 diabetes. Diabetes Obes Metab. 2017;19(6):906-908. doi:10.1111/dom.12885 | Less than 5 studies |
| 1. Stub T, Musial F, Kristoffersen AA, Alræk T, Liu J. Adverse effects of homeopathy, what do we know? A systematic review and meta-analysis of randomized controlled trials. Complement Ther Med. 2016;26:146-163. doi:10.1016/j.ctim.2016.03.013 | No 2 by 2 table data |
| 1. Tanboğa İH, Topçu S, Aksakal E, et al. The Risk of Atrial Fibrillation With Ivabradine Treatment: A Meta-analysis With Trial Sequential Analysis of More Than 40000 Patients. Clin Cardiol. 2016;39(10):615-620. doi:10.1002/clc.22578 | Less than 5 studies |
| 1. Tang H, Fang Z, Wang T, Cui W, Zhai S, Song Y. Meta-Analysis of Effects of Sodium-Glucose Cotransporter 2 Inhibitors on Cardiovascular Outcomes and All-Cause Mortality Among Patients With Type 2 Diabetes Mellitus. Am J Cardiol. 2016;118(11):1774-1780. doi:10.1016/j.amjcard.2016.08.061 | NMA, without pairwise MA |
| 1. Tang H, Li D, Zhang J, et al. Sodium-glucose co-transporter-2 inhibitors and risk of adverse renal outcomes among patients with type 2 diabetes: A network and cumulative meta-analysis of randomized controlled trials. Diabetes Obes Metab. 2017;19(8):1106-1115. doi:10.1111/dom.12917 | NMA, without pairwise MA |
| 1. Tang HL, Li DD, Zhang JJ, et al. Lack of evidence for a harmful effect of sodium-glucose co-transporter 2 (SGLT2) inhibitors on fracture risk among type 2 diabetes patients: a network and cumulative meta-analysis of randomized controlled trials. Diabetes Obes Metab. 2016;18(12):1199-1206. doi:10.1111/dom.12742 | NMA, without pairwise MA |
| 1. Tarp S, Eric Furst D, Boers M, et al. Risk of serious adverse effects of biological and targeted drugs in patients with rheumatoid arthritis: a systematic review meta-analysis. Rheumatology (Oxford). 2017;56(3):417-425. doi:10.1093/rheumatology/kew442 | NMA, without pairwise MA |
| 1. Thakker D, Nair S, Pagada A, Jamdade V, Malik A. Statin use and the risk of developing diabetes: a network meta-analysis. Pharmacoepidemiol Drug Saf. 2016;25(10):1131-1149. doi:10.1002/pds.4020 | NMA, without pairwise MA |
| 1. Thomopoulos C, Parati G, Zanchetti A. Effects of blood-pressure-lowering treatment in hypertension: 9. Discontinuations for adverse events attributed to different classes of antihypertensive drugs: meta-analyses of randomized trials. J Hypertens. 2016;34(10):1921-1932. doi:10.1097/HJH.0000000000001052 | No 2 by 2 table data |
| 1. Tolkien Z, Stecher L, Mander AP, Pereira DI, Powell JJ. Ferrous sulfate supplementation causes significant gastrointestinal side-effects in adults: a systematic review and meta-analysis. PLoS One. 2015;10(2):e0117383. Published 2015 Feb 20. doi:10.1371/journal.pone.0117383 | No 2 by 2 table data |
| 1. Touma L, Filion KB, Atallah R, Eberg M, Eisenberg MJ. A meta-analysis of randomized controlled trials of the risk of bleeding with apixaban versus vitamin K antagonists. Am J Cardiol. 2015;115(4):533-541. doi:10.1016/j.amjcard.2014.11.039 | Less than 5 studies |
| 1. Verrotti A, Prezioso G, Di Sabatino F, Franco V, Chiarelli F, Zaccara G. The adverse event profile of levetiracetam: A meta-analysis on children and adults. Seizure. 2015;31:49-55. doi:10.1016/j.seizure.2015.07.004 | No 2 by 2 table data |
| 1. Vouri SM, Kebodeaux CD, Stranges PM, Teshome BF. Adverse events and treatment discontinuations of antimuscarinics for the treatment of overactive bladder in older adults: A systematic review and meta-analysis. Arch Gerontol Geriatr. 2017;69:77-96. doi:10.1016/j.archger.2016.11.006 | No 2 by 2 table data |
| 1. Wang C, Wang F, Min X, et al. Toxicities of chemoradiotherapy and radiotherapy in nasopharyngeal carcinoma: an updated meta-analysis. J Int Med Res. 2019;47(7):2832-2847. doi:10.1177/0300060519858031 | No 2 by 2 table data |
| 1. Wang XF, Huang WF, Nie J, Zhou Y, Tan DW, Jiang JH. Toxicity of chemotherapy regimens in advanced and metastatic pancreatic cancer therapy: A network meta-analysis. J Cell Biochem. 2018;119(7):5082-5103. doi:10.1002/jcb.26266 | NMA, without pairwise MA |
| 1. Wang Z, Yang X, Wang J, et al. Risk of serious adverse event and fatal adverse event with molecular target anticancer drugs in cancer patients: A meta-analysis. J Cancer Res Ther. 2019;15(7):1435-1449. doi:10.4103/jcrt.JCRT_577_18 | No reference for included studies |
| 1. Wu S, Chai S, Yang J, et al. Gastrointestinal Adverse Events of Dipeptidyl Peptidase 4 Inhibitors in Type 2 Diabetes: A Systematic Review and Network Meta-analysis. Clin Ther. 2017;39(9):1780-1789.e33. doi:10.1016/j.clinthera.2017.07.036 | NMA, without pairwise MA |
| 1. Wu S, Cipriani A, Yang Z, et al. The cardiovascular effect of incretin-based therapies among type 2 diabetes: a systematic review and network meta-analysis. Expert Opin Drug Saf. 2018;17(3):243-249. doi:10.1080/14740338.2018.1424826 | NMA, without pairwise MA |
| 1. Wu Y, Mu Y, Yin L, Wang Z, Liu W, Wan H. Complications in the Management of Acute Achilles Tendon Rupture: A Systematic Review and Network Meta-analysis of 2060 Patients. Am J Sports Med. 2019;47(9):2251-2260. doi:10.1177/0363546518824601 | NMA, without pairwise MA |
| 1. Xu C, Chen YP, Du XJ, et al. Comparative safety of immune checkpoint inhibitors in cancer: systematic review and network meta-analysis. BMJ. 2018;363:k4226. Published 2018 Nov 8. doi:10.1136/bmj.k4226 | NMA, without pairwise MA |
| 1. Xu R, Lian Y, Li WX. Airway Complications during and after General Anesthesia: A Comparison, Systematic Review and Meta-Analysis of Using Flexible Laryngeal Mask Airways and Endotracheal Tubes. PLoS One. 2016;11(7):e0158137. Published 2016 Jul 14. doi:10.1371/journal.pone.0158137 | No 2 by 2 table data |
| 1. Xu X, Zhu H, Lv H. Safety of Staphylococcus aureus four-antigen and three-antigen vaccines in healthy adults: A meta-analysis of randomized controlled trials. Hum Vaccin Immunother. 2018;14(2):314-321. doi:10.1080/21645515.2017.1395540 | Less than 5 studies |
| 1. Yamaguchi N, Fujii T, Aoi S, Kozuch PS, Hortobagyi GN, Blum RH. Comparison of cardiac events associated with liposomal doxorubicin, epirubicin and doxorubicin in breast cancer: a Bayesian network meta-analysis. Eur J Cancer. 2015;51(16):2314-2320. doi:10.1016/j.ejca.2015.07.031 | No 2 by 2 table data |
| 1. Yang M, Zhang Y, Chen H, Lin J, Zeng J, Xu Z. Inhaled corticosteroids and risk of upper respiratory tract infection in patients with asthma: a meta-analysis. Infection. 2019;47(3):377-385. doi:10.1007/s15010-018-1229-y | No reference for included studies |
| 1. Yang X, Pan X, Cheng X, Cheng Y, Kuang Y. Risk of treatment-related mortality with sorafenib in cancer patients: a meta-analysis of 20 randomly controlled trials: Risk of sorafenib-associated death. Int J Clin Pharm. 2015;37(6):1047-1056. doi:10.1007/s11096-015-0151-y | No 2 by 2 table data |
| 1. Yang X, Pan X, Cheng X, Kuang Y, Cheng Y. Risk of Gastrointestinal Events During Vandetanib Therapy in Patients With Cancer: A Systematic Review and Meta-analysis of Clinical Trials. Am J Ther. 2017;24(3):e351-e360. doi:10.1097/MJT.0000000000000306 | No 2 by 2 table data |
| 1. Yang X, Pan X, Cheng X, Kuang Y, Cheng Y. Risk of Gastrointestinal Events During Lapatinib Therapy: A Meta-Analysis From 12,402 Patients With Cancer. Am J Ther. 2018;25(4):e412-e422. doi:10.1097/MJT.0000000000000368 | No 2 by 2 table data |
| 1. Yoon BH, Seo JG, Koo KH. Comparison of Postoperative Infection-Related Complications between Cemented and Cementless Hemiarthroplasty in Elderly Patients: A Meta-Analysis. Clin Orthop Surg. 2017;9(2):145-152. doi:10.4055/cios.2017.9.2.145 | No 2 by 2 table data |
| 1. Yun S, Vincelette ND, Acharya U, Abraham I. Risk of Atrial Fibrillation and Bleeding Diathesis Associated With Ibrutinib Treatment: A Systematic Review and Pooled Analysis of Four Randomized Controlled Trials. Clin Lymphoma Myeloma Leuk. 2017;17(1):31-37.e13. doi:10.1016/j.clml.2016.09.010 | Less than 5 studies |
| 1. Zagmutt FJ, Carroll CA. Meta-analysis of adverse events in recent randomized clinical trials for dimethyl fumarate, glatiramer acetate and teriflunomide for the treatment of relapsing forms of multiple sclerosis. Int J Neurosci. 2015;125(11):798-807. doi:10.3109/00207454.2014.979982 | No 2 by 2 table data |
| 1. Zarrabi K, Wu S. Risk of Liver Toxicity with Nivolumab Immunotherapy in Cancer Patients. Oncology. 2018;94(5):259-273. doi:10.1159/000486679 | No 2 by 2 table data |
| 1. Zhang X, Shao Y, Wang K. Incidence and risk of hypertension associated with cabozantinib in cancer patients: a systematic review and meta-analysis. Expert Rev Clin Pharmacol. 2016;9(8):1109-1115. doi:10.1080/17512433.2016.1190269 | Less than 5 studies |
| 1. Zhang XH, Hao S, Gao B, et al. A network meta-analysis for toxicity of eight chemotherapy regimens in the treatment of metastatic/advanced breast cancer. Oncotarget. 2016;7(51):84533-84543. doi:10.18632/oncotarget.13023 | NMA, without pairwise MA |
| 1. Zhu J, Liao R, Su C, et al. Toxicity profile characteristics of novel androgen-deprivation therapy agents in patients with prostate cancer: a meta-analysis. Expert Rev Anticancer Ther. 2018;18(2):193-198. doi:10.1080/14737140.2018.1419871 | No 2 by 2 table data |
| 1. Zhuang XD, He X, Yang DY, et al. Comparative cardiovascular outcomes in the era of novel anti-diabetic agents: a comprehensive network meta-analysis of 166,371 participants from 170 randomized controlled trials. Cardiovasc Diabetol. 2018;17(1):79. Published 2018 Jun 5. doi:10.1186/s12933-018-0722-z | NMA, without pairwise MA |
| 1. Zis P, Hadjivassiliou M, Sarrigiannis PG, Jenkins TM, Mitsikostas DD. Nocebo in chronic inflammatory demyelinating polyneuropathy; a systematic review and meta-analysis of placebo-controlled clinical trials. J Neurol Sci. 2018;388:79-83. doi:10.1016/j.jns.2018.03.009 | Less than 5 studies |
| 1. 李志霞,武珊珊,杨智荣,詹思延,孙凤.胰高血糖素样肽1受体激动剂类降糖药致2型糖尿病患者鼻咽炎和上呼吸道感染的网状meta分析[J].北京大学学报(医学版),2016,48(03):454-459. | NMA, without pairwise MA |
| 1. Costa R, Zaman S, Sharpe S, Helenowski I, Shaw C, Han H, Soliman H, Czerniecki B. A brief report of toxicity end points of HER2 vaccines for the treatment of patients with HER2+ breast cancer. Drug Des Devel Ther. 2019 Jan 14;13:309-316. | Non-medication interventions |
| 1. Schiphorst AH, Verweij NM, Pronk A, Borel Rinkes IH, Hamaker ME. Non-surgical complications after laparoscopic and open surgery for colorectal cancer - A systematic review of randomised controlled trials. Eur J Surg Oncol. 2015;41(9):1118-1127. | Non-medication interventions |
| 1. Alexander PE, Barty R, Fei Y, et al. Transfusion of fresher vs older red blood cells in hospitalized patients: a systematic review and meta-analysis. Blood. 2016;127(4):400-410. doi:10.1182/blood-2015-09-670950 | Non-medication interventions |
| 1. Toner AJ, Ganeshanathan V, Chan MT, Ho KM, Corcoran TB. Safety of Perioperative Glucocorticoids in Elective Noncardiac Surgery: A Systematic Review and Meta-analysis. Anesthesiology. 2017;126(2):234-248. | Non-medication interventions |
| 1. Sathya C, Wayne C, Gotsch A, Vincent J, Sullivan KJ, Nasr A. Laparoscopic versus open pyloromyotomy in infants: a systematic review and meta-analysis. Pediatr Surg Int. 2017;33(3):325-333. | Non-medication interventions |
| 1. Biardeau X, Zanaty M, Aoun F, Benbouzid S, Peyronnet B. Voies d'abord et complications des bandelettes sous-urétrales synthétiques chez la femme: revue systématique de la littérature et méta-analyse [Approach and complications associated with suburethral synthetic slings in women: Systematic review and meta-analysis]. Prog Urol. 2016;26(4):254-269. | Non-medication interventions |
| 1. Yahav D, Green H, Eliakim-Raz N, Mor E, Husain S. Early double J stent removal in renal transplant patients to prevent urinary tract infection - systematic review and meta-analysis of randomized controlled trials. Eur J Clin Microbiol Infect Dis. 2018;37(4):773-778. doi:10.1007/s10096-017-3173-7 | Non-medication interventions |
| 1. Mohananey D, Sengodan P, Banerjee K, et al. Comparative analysis of cerebrovascular events in transcatheter and surgical aortic valve replacement: a systematic review and meta-analysis of randomised trials. EuroIntervention. 2018;14(1):69-77. doi:10.4244/EIJ-D-17-00732 | Non-medication interventions |
| 1. Elgendy IY, Mahmoud AN, Brilakis ES, Bavry AA. Drug-eluting stents versus bare metal stents for saphenous vein graft revascularisation: a meta-analysis of randomised trials. EuroIntervention. 2018;14(2):215-223. doi:10.4244/EIJ-D-17-00839 | Non-medication interventions |
| 1. Osland E, Yunus RM, Khan S, Memon B, Memon MA. Late Postoperative Complications in Laparoscopic Sleeve Gastrectomy (LVSG) Versus Laparoscopic Roux-en-y Gastric Bypass (LRYGB): Meta-analysis and Systematic Review. Surg Laparosc Endosc Percutan Tech. 2016;26(3):193-201. doi:10.1097/SLE.0000000000000279 | Non-medication interventions |
| 1. Zhao Y, Peng H, Li X, et al. Dual antiplatelet therapy after coronary artery bypass surgery: is there an increase in bleeding risk? A meta-analysis. Interact Cardiovasc Thorac Surg. 2018;26(4):573-582. doi:10.1093/icvts/ivx374 | Non-medication interventions |
| 1. Hua J, He Z, Qian D, Meng H, Zhou B, Song Z. Duct-to-Mucosa Versus Invagination Pancreaticojejunostomy Following Pancreaticoduodenectomy: a Systematic Review and Meta-Analysis. J Gastrointest Surg. 2015;19(10):1900-1909. doi:10.1007/s11605-015-2913-1 | Non-medication interventions |
| 1. Zhao F, Lei R, Yang SK, et al. Comparative effect of iso-osmolar versus low-osmolar contrast media on the incidence of contrast-induced acute kidney injury in diabetic patients: a systematic review and meta-analysis. Cancer Imaging. 2019;19(1):38. Published 2019 Jun 18. doi:10.1186/s40644-019-0224-6 | Non-medication interventions |
| 1. Katsanos K, Spiliopoulos S, Kitrou P, Krokidis M, Karnabatidis D. Risk of Death Following Application of Paclitaxel-Coated Balloons and Stents in the Femoropopliteal Artery of the Leg: A Systematic Review and Meta-Analysis of Randomized Controlled Trials. J Am Heart Assoc. 2018;7(24):e011245. doi:10.1161/JAHA.118.011245 | Non-medication interventions |
| 1. Dinh K, Limmer AM, Paravastu SCV, et al. Mortality After Paclitaxel-Coated Device Use in Dialysis Access: A Systematic Review and Meta-Analysis. J Endovasc Ther. 2019;26(5):600-612. doi:10.1177/1526602819872154 | Non-medication interventions |
| 1. Li DF, Yang MF, Chang X, et al. Endocut Versus Conventional Blended Electrosurgical Current for Endoscopic Biliary Sphincterotomy: A Meta-Analysis of Complications. Dig Dis Sci. 2019;64(8):2088-2094. doi:10.1007/s10620-019-05513-w | Non-medication interventions |
| 1. Lalu MM, Fayad A, Ahmed O, et al. Ultrasound-Guided Subclavian Vein Catheterization: A Systematic Review and Meta-Analysis. Crit Care Med. 2015;43(7):1498-1507. doi:10.1097/CCM.0000000000000973 | Non-medication interventions |
| 1. Parker M, Raval P, Gjertsen JE. Nail or plate fixation for A3 trochanteric hip fractures: A systematic review of randomised controlled trials. Injury. 2018;49(7):1319-1323. doi:10.1016/j.injury.2018.05.017 | Non-medication interventions |
| 1. Franchini M, Mengoli C, Marietta M, et al. Safety of intravenous tranexamic acid in patients undergoing majororthopaedic surgery: a meta-analysis of randomised controlled trials. Blood Transfus. 2018;16(1):36-43. doi:10.2450//2017.0219-17 | Non-medication interventions |
| 1. Mathew PJ, Mathew JL. Early versus late removal of the laryngeal mask airway (LMA) for general anaesthesia. Cochrane Database Syst Rev. 2015;(8):CD007082. Published 2015 Aug 10. doi:10.1002/14651858.CD007082.pub2 | Non-medication interventions |
| 1. Brener MI, Bush A, Miller JM, Hasan RK. Influence of radial versus femoral access site on coronary angiography and intervention outcomes: A systematic review and meta-analysis. Catheter Cardiovasc Interv. 2017;90(7):1093-1104. doi:10.1002/ccd.27043 | Non-medication interventions |
| 1. Henriksen NA, Deerenberg EB, Venclauskas L, et al. Triclosan-coated sutures and surgical site infection in abdominal surgery: the TRISTAN review, meta-analysis and trial sequential analysis. Hernia. 2017;21(6):833-841. doi:10.1007/s10029-017-1681-0 | Non-medication interventions |
| 1. Taglieri N, Bacchi Reggiani ML, Ghetti G, et al. Risk of Stroke in Patients with Stable Coronary Artery Disease Undergoing Percutaneous Coronary Intervention versus Optimal Medical Therapy: Systematic Review and Meta-Analysis of Randomized Controlled Trials. PLoS One. 2016;11(7):e0158769. Published 2016 Jul 8. doi:10.1371/journal.pone.0158769 | Non-medication interventions |
| 1. Riva N, Dentali F, Permunian ET, Ageno W. Major Bleeding and Case Fatality Rate with the Direct Oral Anticoagulants in Orthopedic Surgery: A Systematic Review and Meta-Analysis. Semin Thromb Hemost. 2016;42(1):42-54. doi:10.1055/s-0035-1568875 | Non-medication interventions |
| 1. Wusiman P, Tayie A, Ling W, Moming A. Management of Mandibular Fractures Using Locking and Nonlocking Miniplates [published correction appears in J Craniofac Surg. 2019 Jul;30(5):1593]. J Craniofac Surg. 2019;30(2):448-452. doi:10.1097/SCS.0000000000005128 | Non-medication interventions |
| 1. Scheiermann P, Herzog F, Siebenhofer A, Strametz R, Weberschock T. Intravenous versus inhalational anesthesia for pediatric inpatient surgery - A systematic review and meta-analysis. J Clin Anesth. 2018;49:19-25. doi:10.1016/j.jclinane.2018.05.014 | Non-medication interventions |
| 1. Polderman JA, Farhang-Razi V, Van Dieren S, et al. Adverse side effects of dexamethasone in surgical patients. Cochrane Database Syst Rev. 2018;8(8):CD011940. Published 2018 Aug 28. doi:10.1002/14651858.CD011940.pub2 | Non-medication interventions |
| 1. Bundhun PK, Janoo G, Chen MH. Bleeding events associated with fibrinolytic therapy and primary percutaneous coronary intervention in patients with STEMI: A systematic review and meta-analysis of randomized controlled trials [published correction appears in Medicine (Baltimore). 2016 Jul 18;95(28):e0916]. Medicine (Baltimore). 2016;95(23):e3877. doi:10.1097/MD.0000000000003877 | Non-medication interventions |
| 1. Chow R, Bruera E, Arends J, et al. Enteral and parenteral nutrition in cancer patients, a comparison of complication rates: an updated systematic review and (cumulative) meta-analysis [published correction appears in Support Care Cancer. 2019 Dec 31;:]. Support Care Cancer. 2020;28(3):979-1010. | Non-medication interventions |
| 1. Klotz R, Probst P, Deininger M, et al. Percutaneous versus surgical strategy for tracheostomy: a systematic review and meta-analysis of perioperative and postoperative complications. Langenbecks Arch Surg. 2018;403(2):137-149. doi:10.1007/s00423-017-1648-8 | Non-medication interventions |
| 1. Lewis SR, Butler AR, Parker J, Cook TM, Schofield-Robinson OJ, Smith AF. Videolaryngoscopy versus direct laryngoscopy for adult patients requiring tracheal intubation: a Cochrane Systematic Review. Br J Anaesth. 2017;119(3):369-383. doi:10.1093/bja/aex228 | Non-medication interventions |
| 1. Sun W, Li J. Skin Toxicities with Epidermal Growth Factor Receptor Tyrosine Kinase Inhibitors in Cancer Patients: A Meta-Analysis of Randomized Controlled Trials. Cancer Invest. 2019;37(6):253-264. doi:10.1080/07357907.2019.1634089 | Non-medication interventions |
| 1. Jin S, Zhou X. Influence of dexmedetomidine on cardiac complications in non-cardiac surgery: a meta-analysis of randomized trials. Int J Clin Pharm. 2017;39(4):629-640. doi:10.1007/s11096-017-0493-8 | Non-medication interventions |
| 1. Grajek S, Michalak M, Gwizdała A, et al. Patients treated with bivalirudin are still at higher risk of stent thrombosis: a comprehensive meta-analysis of randomised clinical trials of bivalirudin and heparin for percutaneous coronary interventions. Kardiol Pol. 2018;76(4):740-749. doi:10.5603/KP.a2018.0024 | Non-medication interventions |
| 1. Avni T, Bieber A, Grossman A, Green H, Leibovici L, Gafter-Gvili A. The safety of intravenous iron preparations: systematic review and meta-analysis. Mayo Clin Proc. 2015;90(1):12-23. doi:10.1016/j.mayocp.2014.10.007 | Non-medication interventions |
| 1. Wadhwa V, Issa D, Garg S, Lopez R, Sanaka MR, Vargo JJ. Similar Risk of Cardiopulmonary Adverse Events Between Propofol and Traditional Anesthesia for Gastrointestinal Endoscopy: A Systematic Review and Meta-analysis. Clin Gastroenterol Hepatol. 2017;15(2):194-206. doi:10.1016/j.cgh.2016.07.013 | Non-medication interventions |
| 1. Zhang W, Lu M, Zhang C, et al. Therapeutic hypothermia increases the risk of cardiac arrhythmia for perinatal hypoxic ischaemic encephalopathy: A meta-analysis. PLoS One. 2017;12(3):e0173006. Published 2017 Mar 8. doi:10.1371/journal.pone.0173006 | Non-medication interventions |
| 1. Yuan ZZ, Yang Z, Liu Q, Liu YM. Complications following open reduction and internal fixation versus external fixation in treating unstable distal radius fractures: Grading the evidence through a meta-analysis. Orthop Traumatol Surg Res. 2018;104(1):95-103. doi:10.1016/j.otsr.2017.08.020 | Non-medication interventions |
| 1. Malihi Z, Wu Z, Lawes CMM, Scragg R. Adverse events from large dose vitamin D supplementation taken for one year or longer. J Steroid Biochem Mol Biol. 2019;188:29-37. doi:10.1016/j.jsbmb.2018.12.002 | Non-medication interventions |
| 1. Malihi Z, Wu Z, Mm Lawes C, Scragg R. Noncalcemic adverse effects and withdrawals in randomized controlled trials of long-term vitamin D2 or D3 supplementation: a systematic review and meta-analysis. Nutr Rev. 2017;75(12):1007-1034. doi:10.1093/nutrit/nux059 | Non-medication interventions |
| 1. Malihi Z, Wu Z, Stewart AW, Lawes CM, Scragg R. Hypercalcemia, hypercalciuria, and kidney stones in long-term studies of vitamin D supplementation: a systematic review and meta-analysis. Am J Clin Nutr. 2016;104(4):1039-1051. doi:10.3945/ajcn.116.134981 | Non-medication interventions |
| 1. Tang Z, Yang Y, Yang Z, Meng W, Li X. Early precut sphincterotomy does not increase the risk of adverse events for patients with difficult biliary access: A systematic review of randomized clinical trials with meta-analysis and trial sequential analysis. Medicine (Baltimore). 2018;97(36):e12213. doi:10.1097/MD.0000000000012213 | Non-medication interventions |
| 1. Carvalho ÍT, Baccaglini W, Claros OR, et al. Genitourinary and gastrointestinal toxicity among patients with localized prostate cancer treated with conventional versus moderately hypofractionated radiation therapy: systematic review and meta-analysis. Acta Oncol. 2018;57(8):1003-1010. doi:10.1080/0284186X.2018.1478126 | Non-medication interventions |
| 1. Jiang J, Zou J, Ma H, et al. Network Meta-analysis of Randomized Trials on the Safety of Vascular Closure Devices for Femoral Arterial Puncture Site Haemostasis. Sci Rep. 2015;5:13761. Published 2015 Sep 8. doi:10.1038/srep13761 | Non-medication interventions |
| 1. Ruan SY, Huang TM, Wu HY, Wu HD, Yu CJ, Lai MS. Inhaled nitric oxide therapy and risk of renal dysfunction: a systematic review and meta-analysis of randomized trials. Crit Care. 2015;19(1):137. Published 2015 Apr 3. doi:10.1186/s13054-015-0880-2 | Non-medication interventions |
| 1. Thong KM, Chan TM. Infectious complications in lupus nephritis treatment: a systematic review and meta-analysis. Lupus. 2019;28(3):334-346. doi:10.1177/0961203319829817 | Non-medication interventions |
| 1. Uhlig C, Bluth T, Schwarz K, et al. Effects of Volatile Anesthetics on Mortality and Postoperative Pulmonary and Other Complications in Patients Undergoing Surgery: A Systematic Review and Meta-analysis. Anesthesiology. 2016;124(6):1230-1245. doi:10.1097/ALN.0000000000001120 | Non-medication interventions |
| 1. Zhang T, Pope JE. Cardiovascular effects of urate-lowering therapies in patients with chronic gout: a systematic review and meta-analysis. Rheumatology (Oxford). 2017;56(7):1144-1153. doi:10.1093/rheumatology/kex065 | Non-medication interventions |
| 1. Zhao J, Xia Y, Kaminski J, et al. Treatment-Related Death during Concurrent Chemoradiotherapy for Locally Advanced Non-Small Cell Lung Cancer: A Meta-Analysis of Randomized Studies. PLoS One. 2016;11(6):e0157455. | Non-medication interventions |
| 1. 薛晓静,何饶丽,李伟兴,辛佳蔚,叶钦勇,陈晓春,潘晓东.非典型抗精神病药治疗痴呆精神行为症状安全性的系统评价[J].中华医学杂志,2018,98(25):2030-2036. | Non-medication interventions |

**Fig.S1.** The process of moderator harmonization.

**
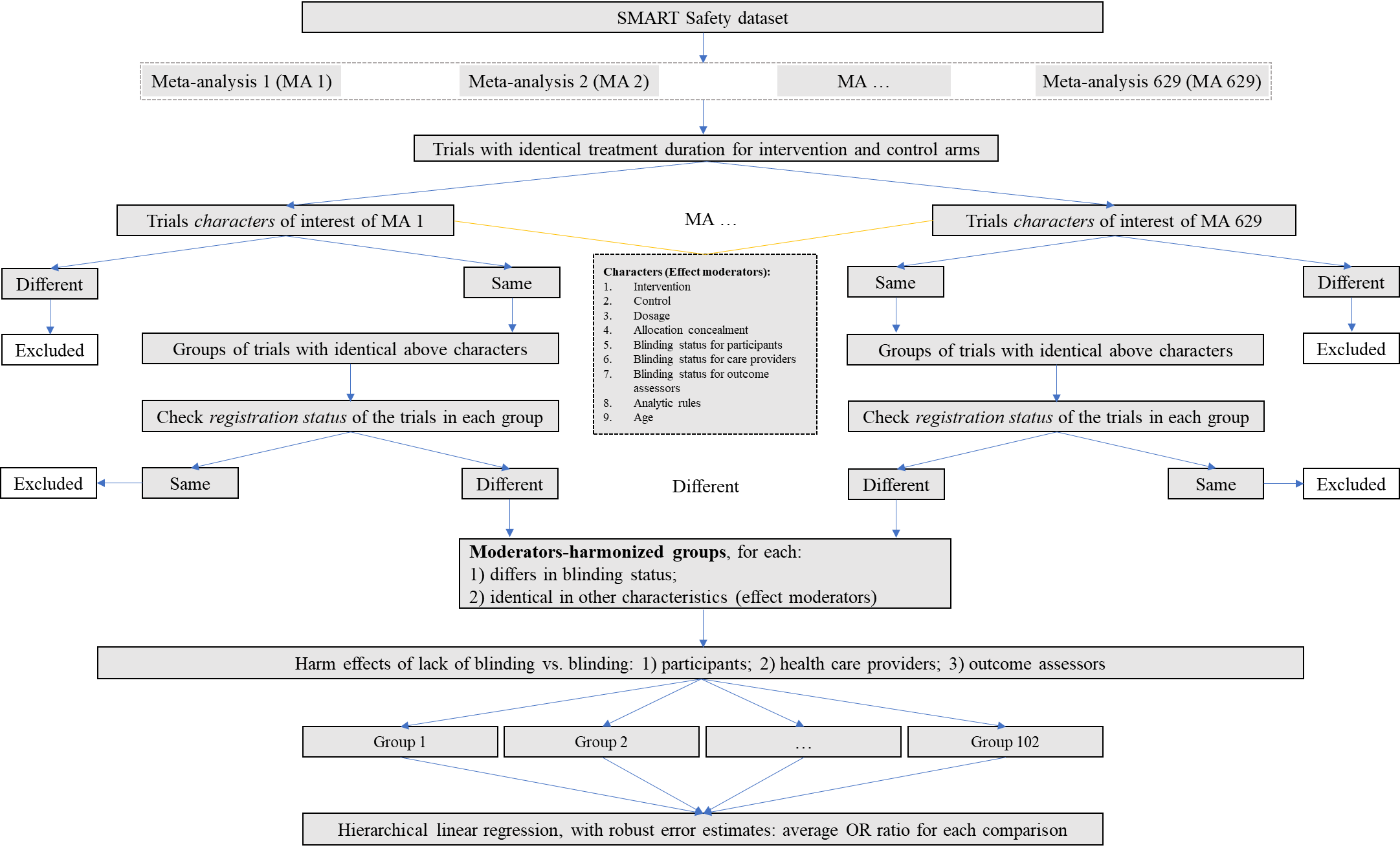
**

## Cause path analysis

We employed the by directed acyclic graph (DAG) to abtain the casual paths involved in the analysis, as an effort to detect the potential variables that need to be addressed. The following variables were identified through referring previous literatures that may impact the effects of the adverse events: 1) inconsistent interventions; 2) inconsistent controls; 3) variant variation in dosage of the intervention (mean dose per week); 4) imbalanced treatment duration; 5) population age (child, adult, elderly); 6) funding (source of funding, role of funding); 7) cluster effect of trials (multiple centers over single center); 8) registration or not; 9) analytic principle (Intention-to-treat (ITT), per-protocol (PP), and as-treated (AT)); 10) Diseases (type, severity, combined condition, et al). For the these variables, we had no sufficient information on role of funding and diseases due to the limited information reported in original trials. Figure S1 presents the DAG plot.

## Fig.S2. The DAG plot for identifying potential effect modifiers (Blind for participants and health care providers).


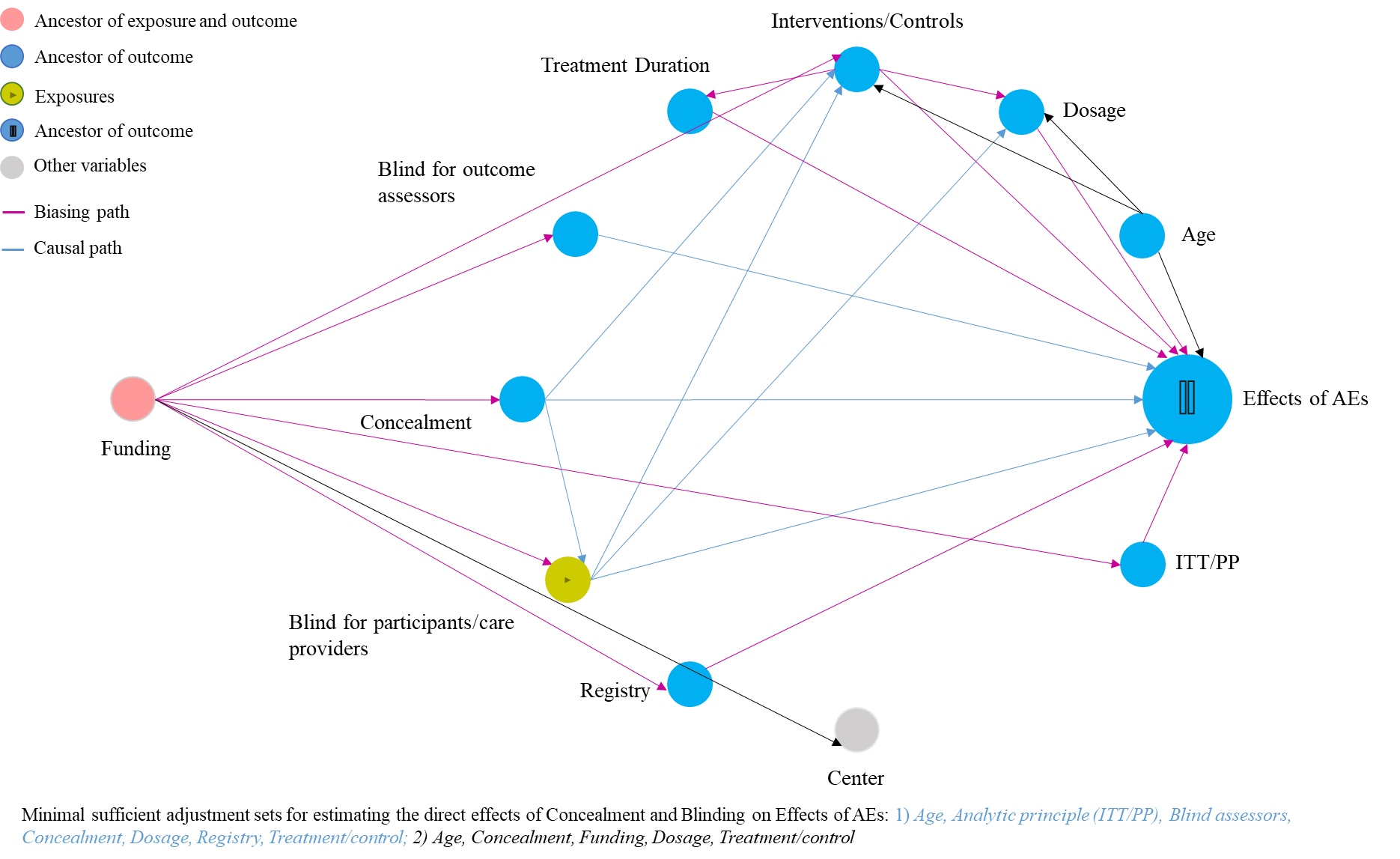


## Fig.S3. The DAG plot for identifying potential effect modifiers (Blind for outcome assessors).


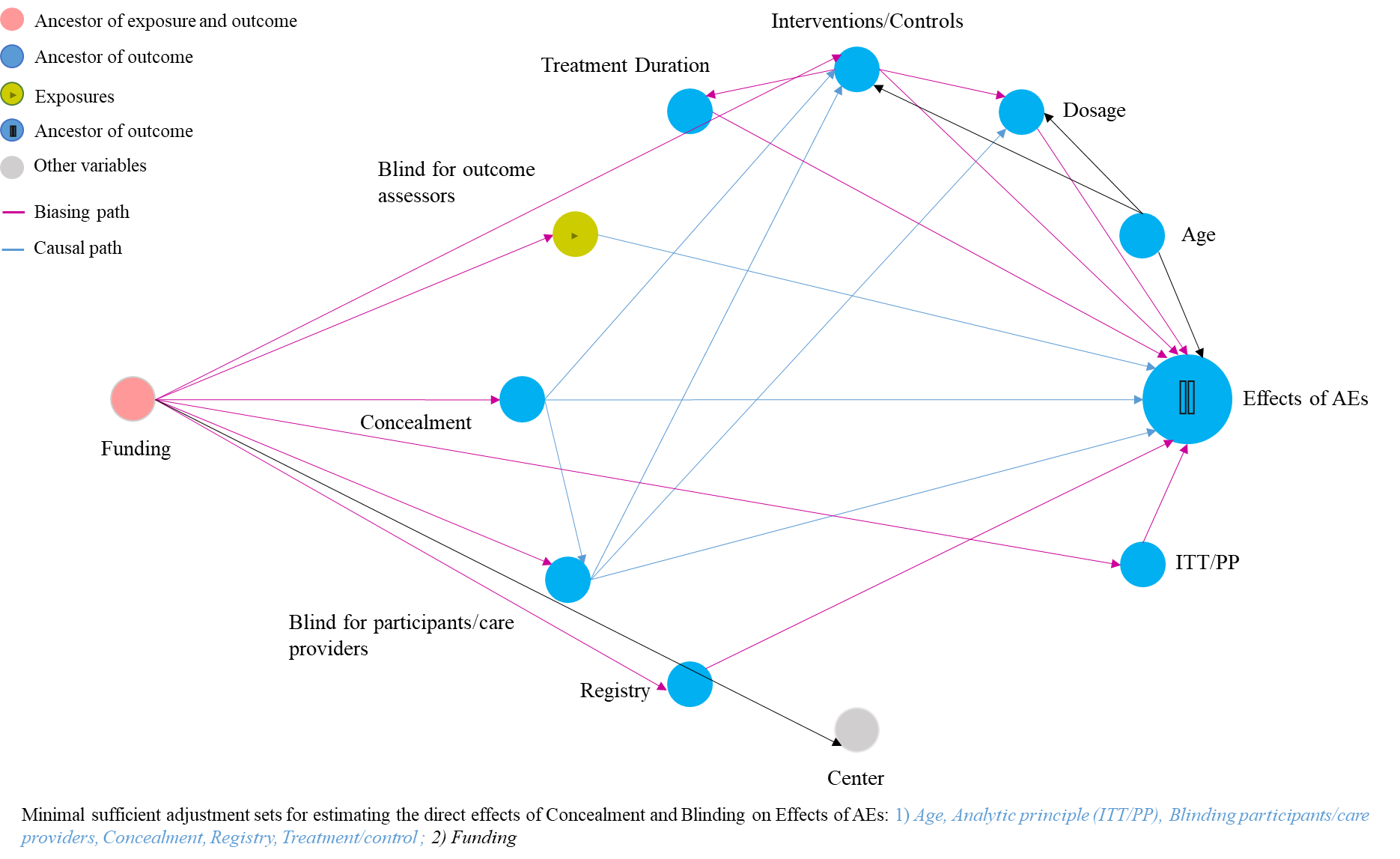


## The DAG model code (http://dagitty.net/).

*dag {*

*bb="0,0,1,1"*

*"Analytic principle (ITT/PP)" [pos="0.377,0.685"]*

*"Blind assessors" [exposure,pos="0.573,0.514"]*

*"Blinding participants/care providers" [pos="0.380,0.444"]*

*"Conflicts of interests" [pos="0.065,0.468"]*

*"Effects of AE" [outcome,pos="0.768,0.417"]*

*"Treatment/control" [pos="0.193,0.083"]*

*Age [pos="0.614,0.083"]*

*Concealment [pos="0.211,0.446"]*

*Dosage [pos="0.413,0.257"]*

*Duration [pos="0.480,0.365"]*

*Registry [pos="0.377,0.816"]*

*"Analytic principle (ITT/PP)" -> "Effects of AE"*

*"Blind assessors" -> "Effects of AE"*

*"Blinding participants/care providers" -> "Effects of AE"*

*"Blinding participants/care providers" -> "Treatment/control"*

*"Blinding participants/care providers" -> Dosage*

*"Conflicts of interests" -> "Analytic principle (ITT/PP)"*

*"Conflicts of interests" -> "Blind assessors" [pos="0.453,0.605"]*

*"Conflicts of interests" -> "Blinding participants/care providers" [pos="0.155,0.301"]*

*"Conflicts of interests" -> "Treatment/control"*

*"Conflicts of interests" -> Concealment*

*"Conflicts of interests" -> Registry*

*"Treatment/control" -> "Effects of AE"*

*"Treatment/control" -> Dosage*

*"Treatment/control" -> Duration*

*Age -> "Effects of AE"*

*Age -> "Treatment/control"*

*Age -> Dosage*

*Concealment -> "Blinding participants/care providers"*

*Concealment -> "Effects of AE" [pos="0.337,0.542"]*

*Concealment -> "Treatment/control"*

*Dosage -> "Effects of AE"*

*Duration -> "Effects of AE"*

*Registry -> "Effects of AE"*

*}*

## Code for data analysis (STATA)

****drop those with missing data in metadata (r1/n1/r2/n2/t1/t2)*

*drop if r1 ==. | r2 ==. | n1 ==. | n2 ==. | t1 ==. | t2 ==.*

*drop if r1 == 0 & r2 == 0 & n1 == 0 & n2 ==0*

*drop if t1 ==. | t2 == .*

*keep if fulltex =="Y"*

*replace ittpp = "ITT" if ittpp == "mITT"*

*replace ittpp = "PP" if ittpp == "AT"*

*replace age = "Adult" if age =="Old"*

*replace age = "NA" if age ==""*

**sensitivity analysis (removing zero-event studies)*

**drop if r1 == 0 | r2 ==0*

*gen conceal =.*

*replace conceal = 0 if concealment =="N"*

*replace conceal = 0 if concealment =="NI"*

*replace conceal = 1 if concealment =="PY"*

*replace conceal = 1 if concealment =="Y"*

*gen mask_care =.*

*replace mask_care = 0 if blind_care =="N"*

*replace mask_care = 0 if blind_care =="NI"*

*replace mask_care = 1 if blind_care =="PY"*

*replace mask_care = 1 if blind_care =="Y"*

*gen mask_assor =.*

*replace mask_assor = 0 if blind_out =="N"*

*replace mask_assor = 0 if blind_out =="NI"*

*replace mask_assor = 1 if blind_out =="PY"*

*replace mask_assor = 1 if blind_out =="Y"*

****Limited trials within a meta-analysis with identical intervention, control, and dosage*

*egen match = group(maid net_i net_c dose_indicator age ittpp registry conceal mask_assor)*

*bysort match: gen pair_id = _n*

*bysort match: egen maxpid = max(pair_id)*

*drop if match ==.*

*drop if maxpid == 1*

*egen pairs = group(match)*

*drop maxpid*

*sort maid pairs pair_id*

****Generate agents for concealment and blinding*

*gen mask_pat =.*

*replace mask_pat = 0 if blind_patient =="N"*

*replace mask_pat = 0 if blind_patient =="NI"*

*replace mask_pat = 1 if blind_patient =="PY"*

*replace mask_pat = 1 if blind_patient =="Y"*

****drop those with the same value*

*bysort pairs: egen maxv = max(mask_pat)*

*bysort pairs: egen minv = min(mask_pat)*

*gen d = maxv - minv*

*drop if d == 0*

*egen groups = group(pairs)*

****Get OR and se for each RCT*

*gen logor = log((r1/(n1-r1)) / (r2/(n2-r2)))*

*gen se = sqrt(1/r1 + 1/r2 + 1/(n1-r1) + 1/(n2-r2))*

*replace logor = log(((r1 + 0.5)/(n1-r1 + 0.5)) / ((r2 + 0.5)/(n2-r2 + 0.5))) if (r1 == 0 | r2 ==0)*

*replace se = sqrt(1/(r1 +0.5) + 1/(r2+0.5) + 1/(n1-r1 + 0.5) + 1/(n2-r2 + 0.5)) if (r1 == 0 | r2 ==0)*

*gen wt = 1/se^2*

****regression*

*meglm logor ib1.mask_pat [iw = wt], ||pairs:, vce(cluster maid) eform (exp beta)*

****subgroup analysis*

*bysort outcomeindicator: meglm logor ib1.mask_pat [iw = wt], ||pairs:, vce(cluster maid) eform (exp beta)*

**Fig.S4.** The word cloud of harm outcomes of the SMART Safety dataset.

**
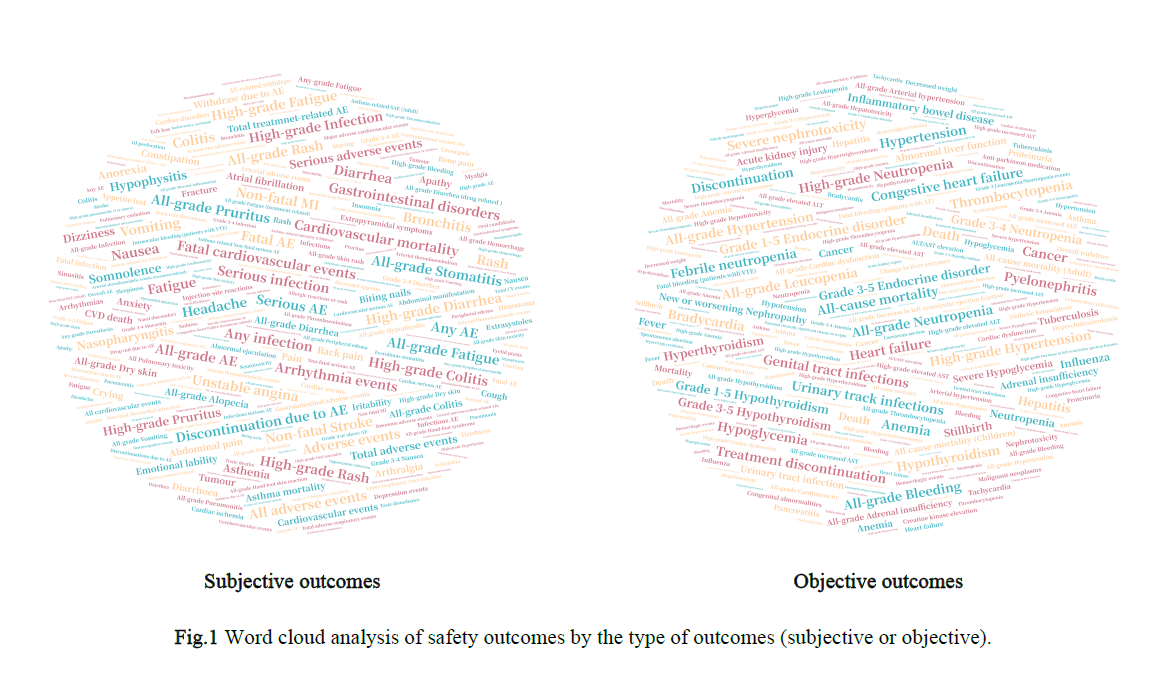
**

**(From:** Fan S, Yu T, Yang X, Zhang R, Furuya-Kanamori L, Xu C. The SMART Safety: An empirical dataset for evidence synthesis of adverse events. Data Brief. 2023 Oct 4;51:109639**.)**
